# Supplementary material for: Exon-intron boundary inhibits m6A deposition, enabling m6A distribution hallmark, longer mRNA half-life and flexible protein coding
Source: Nat Commun. 2023 Jul 13;14:4172. doi: 10.1038/s41467-023-39897-1 (PMC10345190; doi:10.1038/s41467-023-39897-1)
Supplement: Supplementary file 1 — Supplementary Information [file 41467_2023_39897_MOESM1_ESM.pdf]

Exon-intron boundary inhibits m^6A deposition, enabling m^6A distribution hallmark, longer mRNA half-life and flexible protein coding

Supplementary Information

This PDF includes:

Supplementary Figures S1 to S20

Supplementary Table1

Supplementary Fig1

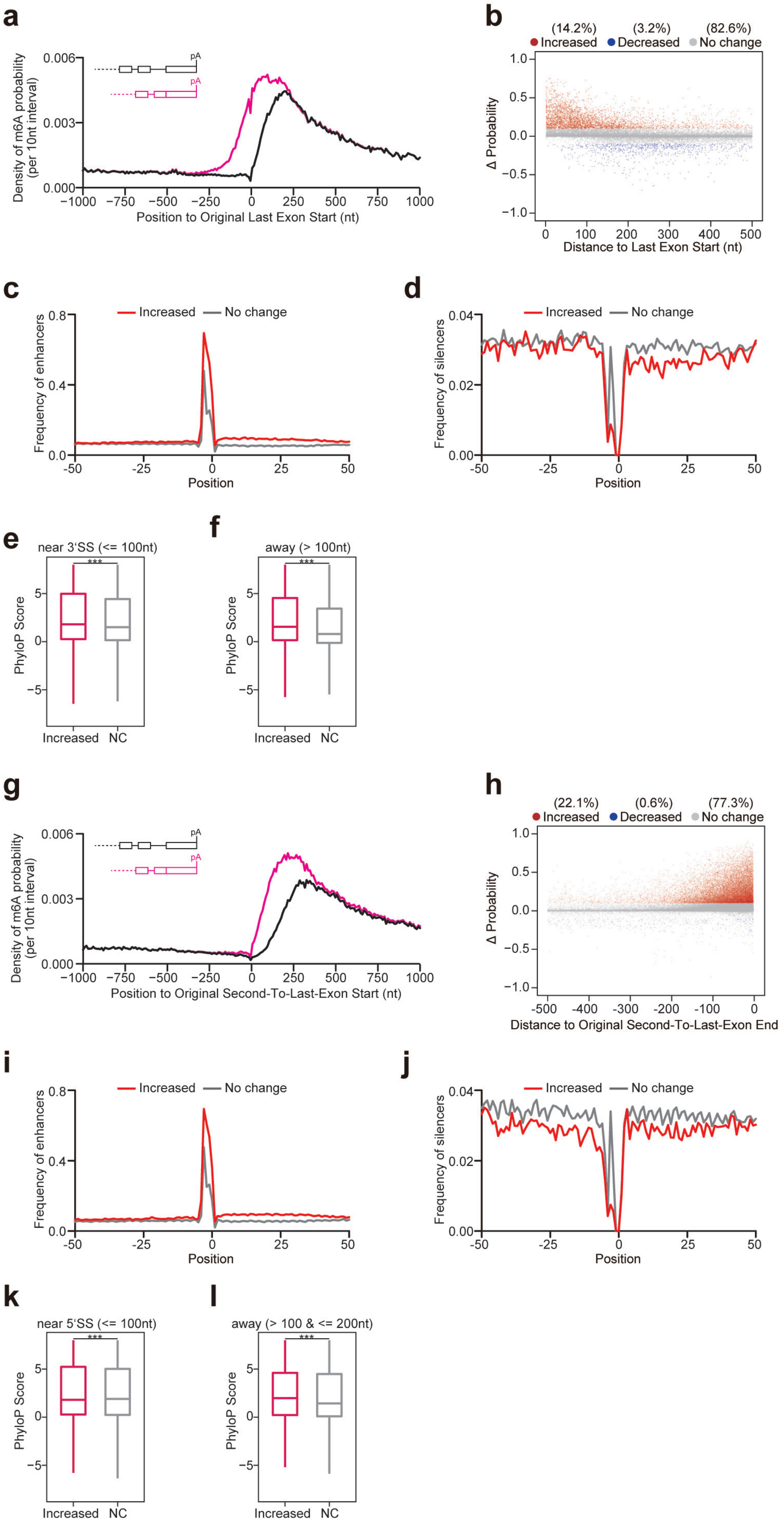

Supplementary Fig. 1: Deep learning modeling reveals last intron deletion promotes m^{6}A deposition at last exon and second-to-last exon (human data).

a, The m^{6}A density of transcripts around last exon start were compared between full length (black line) and last intron deletion (pink line). The value was calculated as the total probability value in a 10-nt interval divided by the total number of mRNAs in the interval.

b, Positional plot of ΔProbability for the RAC sites located in the first 500 nt region of last exons. Red dots (Increased: n = 3444 for 2000 genes), blue dots (Decreased: n = 773 for 2000 genes), and grey dots (no change: n = 19960 for 2000 genes) were those sites that had increased probability (> 0.1), decreased probability (< -0.1), or not change probability (|ΔProbability| <= 0.1) respectively by last intron deletion.

c-d, Positional plot for the frequency of top 50 m^{6}A enhancers (Supplementary Fig. 1c), m^{6}A silencers (Supplementary Fig. 1d) in mRNA sequences around the RAC sites. The sites were located in last exons, and the plots were compared between the increased sites (red line, ΔProbability > 0.1) and no change sites (grey line, |ΔProbability| <= 0.1).

e-f, Box plot of PhyloP score of latent m^{6}A sites or no change sites in near 3'SS (Supplementary Fig. 1e) or away (Supplementary Fig. 1f) from last exon start (n = 14256 or 29632 for Supplementary Fig. 1e, n = 4992 or 100007 for Supplementary Fig. 1f). The box represents the 1st to 3rd quartile with the median marked by a horizontal line. The P-values were calculated by two-sided Wilcoxon test ($p < 2.22e^{-16}$ for Supplementary Fig. 1e,f. Significance: *** for p < 0.001).

g, The m^{6}A density of transcripts around original second-to-last exon start were compared between full length (black line) and last intron deletion (pink line). The value was calculated as the total probability value in a 10-nt interval divided by the total number of mRNAs in the interval.

h, Positional plot of ΔProbability for the RAC sites located in the last 500 nt

region of original second-to-last exons. Red dots (Increased: n = 17521 for 17313 genes), blue dots (Decreased: n = 473 for 17313 genes), and grey dots (no change: n = 61423 for 17313 genes) were those sites that had increased probability (> 0.1), decreased probability (< -0.1), or not change probability (|ΔProbability| <= 0.1) respectively by last intron deletion.

i-j, Positional plot for the frequency of top 50 m^6A enhancers (Supplementary Fig. 1i), m^6A silencers (Supplementary Fig. 1j) in mRNA sequences around the RAC sites. The sites were located in second-to-last exon, and the plots were compared between the increase sites (red line, ΔProbability > 0.1) and no change sites (grey line, |ΔProbability| <= 0.1).

k-l, Box plot of PhyloP score of latent m^6A sites or no change sites in near 5'SS (Supplementary Fig. 1k) or away (Supplementary Fig. 1l) from second-to-last exon end (n = 11778 or 28995 for Supplementary Fig. 1k, n = 1144 or 3209 for Supplementary Fig. 1l). The box represents the 1st to 3rd quartile with the median marked by a horizontal line. The P-values were calculated by two-sided Wilcoxon test ($p = 4.4e^{-7}$ for Supplementary Fig. 1k, $p = 6.4e^{-5}$ for Supplementary Fig. 1l. Significance: *** for p < 0.001).

Supplementary Fig.2

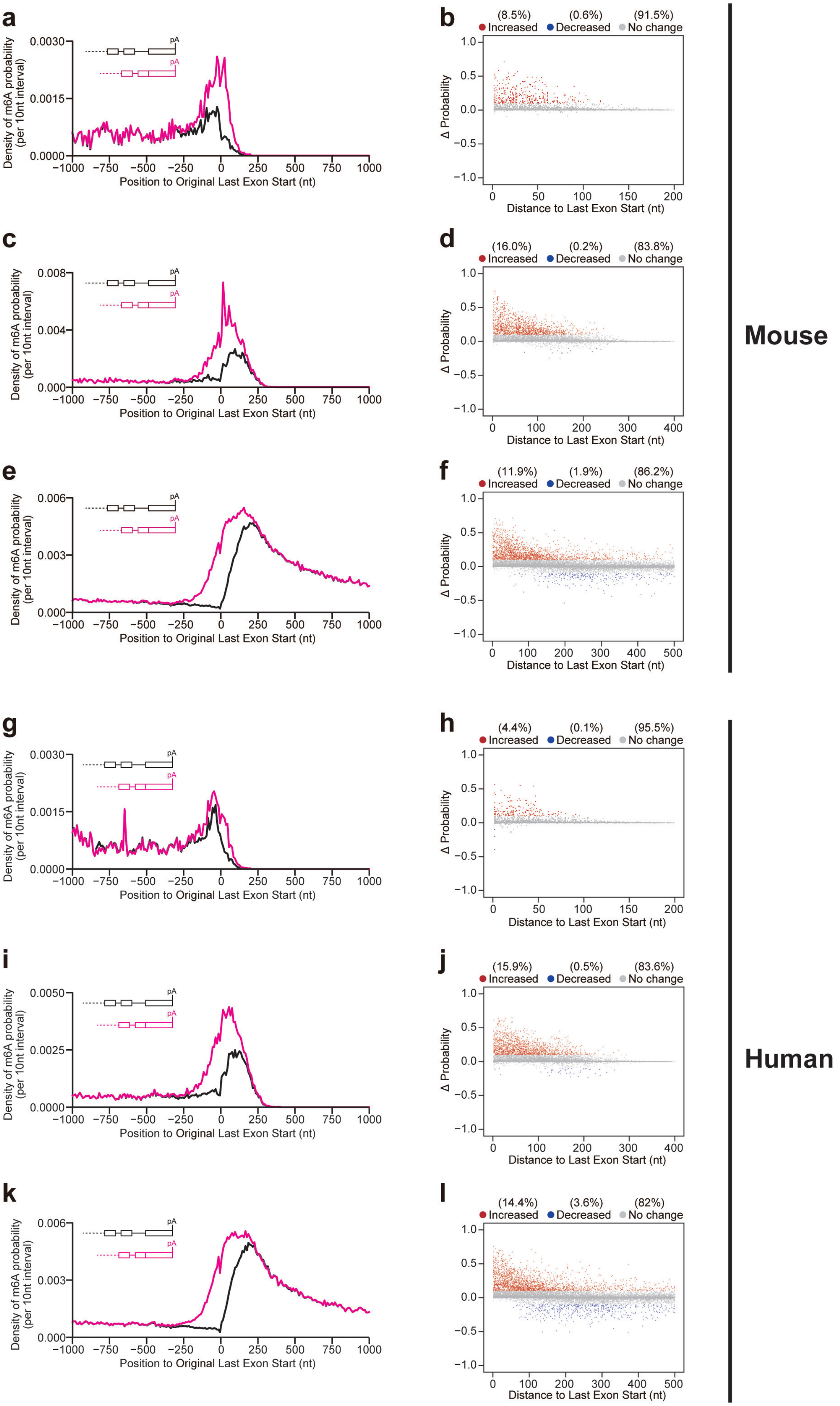

Supplementary Fig. 2: Deep learning modeling reveals last intron deletion promotes m^{6}A deposition at last exon

a, The m^{6}A density of transcripts (last exon length: <= 200 nt) around last exon start were compared between full length (black line) and last intron deletion (pink line). The value was calculated as the total probability value in a 10-nt interval divided by the total number of mRNAs in the interval.

b, Positional plot of ΔProbability for the RAC sites located in the first 200 nt region of last exons (length <= 200 nt). Red dots (Increased: n = 380 for 985 genes), blue dots (Decreased: n = 0 for 985 genes), and grey dots (no change: n = 4097 for 985 genes) were those sites that had increased probability (> 0.1), decreased probability (< -0.1), or not change probability (|ΔProbability| <= 0.1) respectively by last intron deletion.

c, The m^{6}A density of transcripts (last exon length: > 200 nt & < 400 nt) around last exon start were compared between full length (black line) and last intron deletion (pink line). The value was calculated as the total probability value in a 10-nt interval divided by the total number of mRNAs in the interval.

d, Positional plot of ΔProbability for the RAC sites located in the first 400 nt region of last exons (length: > 200 nt & < 400 nt). Red dots (Increased: n = 1326 for 1000 genes), blue dots (Decreased: n = 17 for 1000 genes), and grey dots (no change: n = 6967 for 1000 genes) were those sites that had increased probability (> 0.1), decreased probability (< -0.1), or not change probability (|ΔProbability| <= 0.1) respectively by last intron deletion.

e, The m^{6}A density of transcripts (last exon length: >= 400 nt) around last exon start were compared between full length (black line) and last intron deletion (pink line). The value was calculated as the total probability value in a 10-nt interval divided by the total number of mRNAs in the interval.

f, Positional plot of ΔProbability for the RAC sites located in the first 500 nt region of last exons (length: > 400 nt). Red dots (Increased: n = 1642 for 1000 genes), blue dots (Decreased: n = 261 for 1000 genes), and grey dots (no change: n = 11927 for 1000 genes) were those sites that had increased

probability (> 0.1), decreased probability (< -0.1), or not change probability (|ΔProbability| <= 0.1) respectively by last intron deletion.

g, The m^{6}A density of transcripts (last exon length: <= 200 nt) around last exon start were compared between full length (black line) and last intron deletion (pink line). The value was calculated as the total probability value in a 10-nt interval divided by the total number of mRNAs in the interval.

h, Positional plot of ΔProbability for the RAC sites located in the first 200 nt region of last exons (length <= 200 nt). Red dots (Increased: n = 161 for 843 genes), blue dots (Decreased: n = 4 for 843 genes), and grey dots (no change: n = 3489 for 843 genes) were those sites that had increased probability (> 0.1), decreased probability (< -0.1), or not change probability (|ΔProbability| <= 0.1) respectively by last intron deletion.

i, The m^{6}A density of transcripts (last exon length: > 200 nt & < 400 nt) around last exon start were compared between full length (black line) and last intron deletion (pink line). The value was calculated as the total probability value in a 10-nt interval divided by the total number of mRNAs in the interval.

j, Positional plot of ΔProbability for the RAC sites located in the first 400 nt region of last exons (length: > 200 nt & < 400 nt). Red dots (Increased: n = 1265 for 1000 genes), blue dots (Decreased: n = 41 for 1000 genes), and grey dots (no change: n = 6655 for 1000 genes) were those sites that had increased probability (> 0.1), decreased probability (< -0.1), or not change probability (|ΔProbability| <= 0.1) respectively by last intron deletion.

k, The m^{6}A density of transcripts (last exon length: > 400 nt) around last exon start were compared between full length (black line) and last intron deletion (pink line). The value was calculated as the total probability value in a 10-nt interval divided by the total number of mRNAs in the interval.

l, Positional plot of ΔProbability for the RAC sites located in the first 500 nt region of last exons (length: >= 400 nt). Red dots (Increased: n = 1881 for 1000 genes), blue dots (Decreased: n = 467 for 1000 genes), and grey dots (no change: n = 10678 for 1000 genes) were those sites that had increased

probability (> 0.1), decreased probability (< -0.1), or not change probability (|ΔProbability| <= 0.1) respectively by last intron deletion.

Supplementary Fig.3

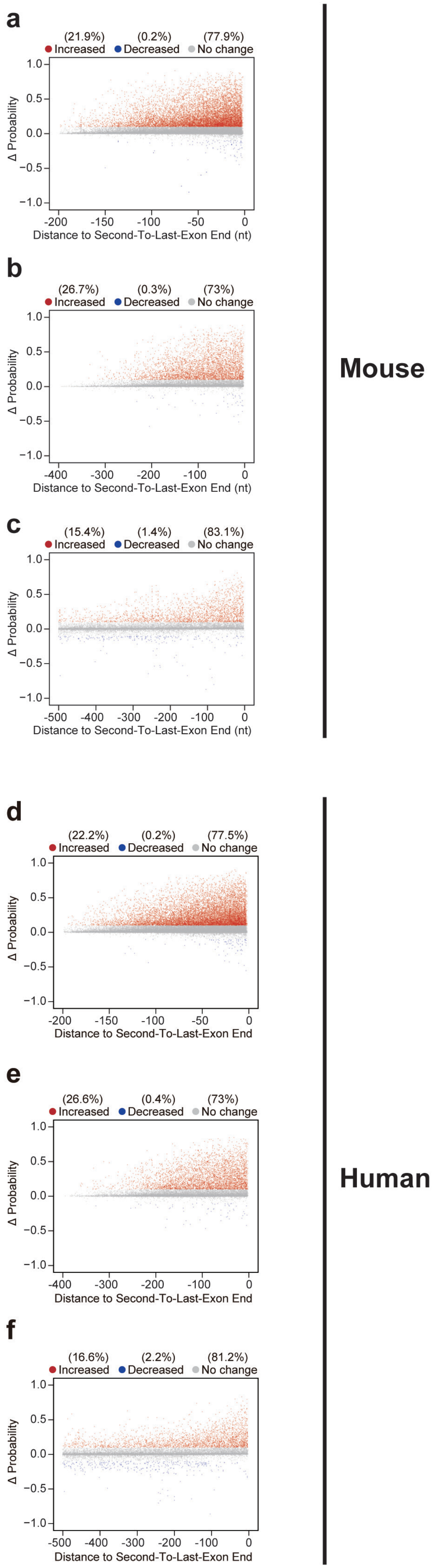

Supplementary Fig. 3: Deep learning modeling reveals last intron deletion promotes m^6A deposition at second-to-last exon

a, Positional plot of ΔProbability for the RAC sites located in the last 200 nt region of second-to-last exons (length: <= 200 nt). Red dots (Increased: n = 10196 for 13367 genes), blue dots (Decreased: n = 97 for 13367 genes), and grey dots (no change: n = 36368 for 13367 genes) were those sites that had increased probability (> 0.1), decreased probability (< -0.1), or not change probability (|ΔProbability| <= 0.1) respectively by last intron deletion.

b, Positional plot of ΔProbability for the RAC sites located in the last 400 nt region of l second-to-last exons (length: > 200 nt & < 400 nt). Red dots (Increased: n = 3962 for 2270 genes), blue dots (Decreased: n = 47 for 2270 genes), and grey dots (no change: n = 10839 for 2270 genes) were those sites that had increased probability (> 0.1), decreased probability (< -0.1), or not change probability (|ΔProbability| <= 0.1) respectively by last intron deletion.

c, Positional plot of ΔProbability for the RAC sites located in the last 500 nt region of second-to-last exons (length: >= 400 nt). Red dots (Increased: n = 2184 for 1126 genes), blue dots (Decreased: n = 203 for 1126 genes), and grey dots (no change: n = 11770 for 1126 genes) were those sites that had increased probability (> 0.1), decreased probability (< -0.1), or not change probability (|ΔProbability| <= 0.1) respectively by last intron deletion.

d, Positional plot of ΔProbability for the RAC sites located in the last 200 nt region of second-to-last exons (length: <= 200 nt). Red dots (Increased: n = 10964 for 13848 genes), blue dots (Decreased: n = 90 for 13848 genes), and grey dots (no change: n = 38152 for 13848 genes) were those sites that had increased probability (> 0.1), decreased probability (< -0.1), or not change probability (|ΔProbability| <= 0.1) respectively by last intron deletion.

e, Positional plot of ΔProbability for the RAC sites located in the last 400 nt region of second-to-last exons (length: > 200 nt & < 400 nt). Red dots (Increased: n = 4105 for 2270 genes), blue dots (Decreased: n = 59 for 2270 genes), and grey dots (no change: n = 11279 for 2270 genes) were those sites

that had increased probability (> 0.1), decreased probability (< -0.1), or not change probability (|ΔProbability| <= 0.1) respectively by last intron deletion.

f, Positional plot of ΔProbability for the RAC sites located in the last 500 nt region of second-to-last exons (length: >= 400 nt). Red dots (Increased: n = 2452 for 1195 genes), blue dots (Decreased: n = 324 for 1195 genes), and grey dots (no change: n = 11992 for 1195 genes) were those sites that had increased probability (> 0.1), decreased probability (< -0.1), or not change probability (|ΔProbability| <= 0.1) respectively by last intron deletion.

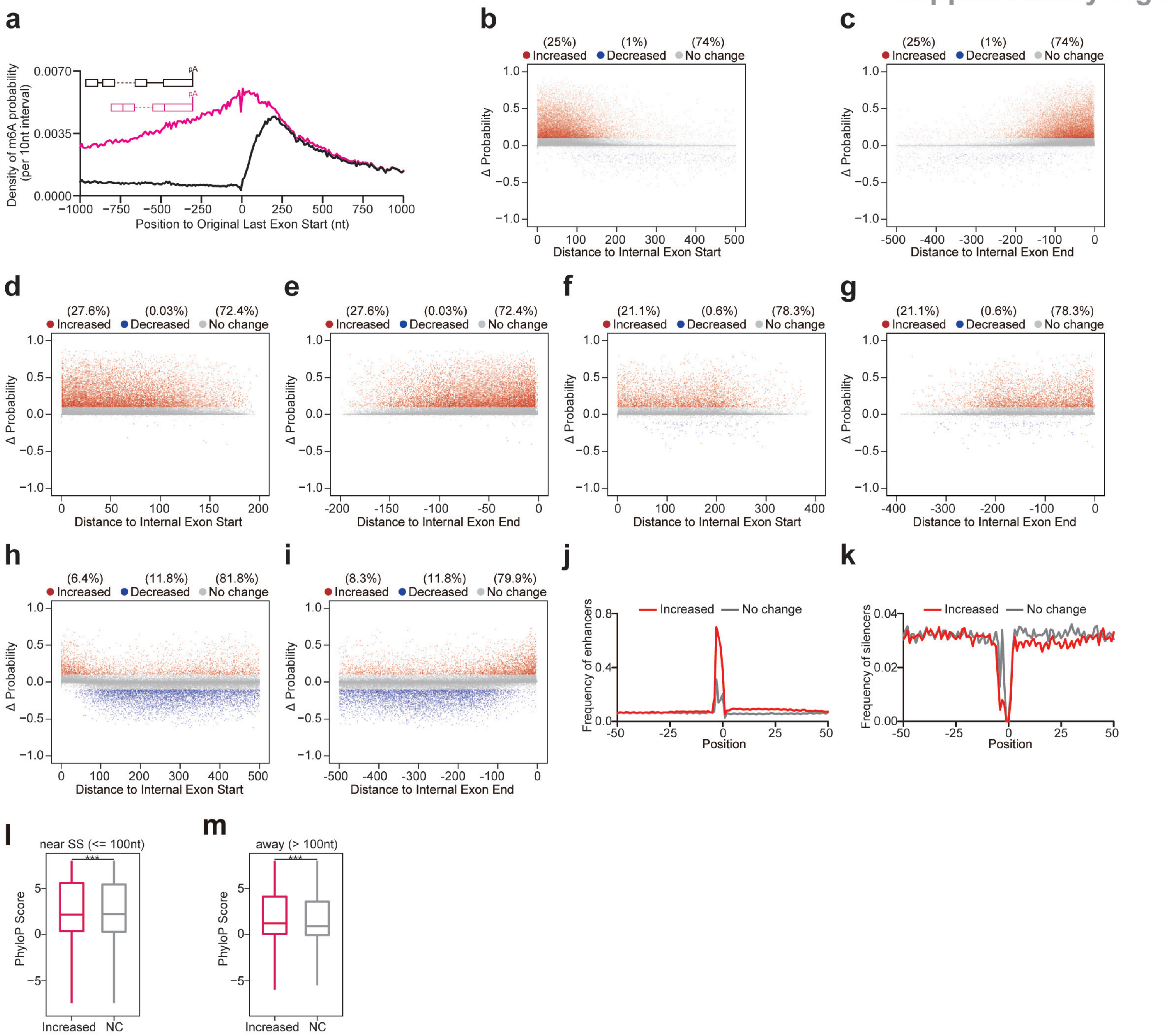
a
Density of m6A probability (per 10nt interval)
pA
Position to Original Last Exon Start (nt)
b
(25%) Increased (1%) Decreased (74%) No change
Δ Probability
Distance to Internal Exon Start
c
(25%) Increased (1%) Decreased (74%) No change
Distance to Internal Exon End
d
(27.6%) Increased (0.03%) Decreased (72.4%) No change
Distance to Internal Exon Start
e
(27.6%) Increased (0.03%) Decreased (72.4%) No change
Distance to Internal Exon End
f
(21.1%) Increased (0.6%) Decreased (78.3%) No change
Distance to Internal Exon Start
g
(21.1%) Increased (0.6%) Decreased (78.3%) No change
Distance to Internal Exon End
h
(6.4%) Increased (11.8%) Decreased (81.8%) No change
Distance to Internal Exon Start
i
(8.3%) Increased (11.8%) Decreased (79.9%) No change
Distance to Internal Exon End
j
Increased No change
Frequency of enhancers
Position
k
Increased No change
Frequency of silencers
Position
l
near SS (<= 100nt)
PhyloP Score
Increased NC
m
away (> 100nt)
PhyloP Score
Increased NC

Supplementary Fig. 4: Deep learning modeling reveals introns deletion revives m^{6}A deposition at internal exons (human data).

a, The m^{6}A density of transcripts around last exon start were compared between full length (black line) and all introns deletion (pink line).

b, Positional plot of ΔProbability for the RAC sites located in the first 500 nt region of internal exons. Red dots (Increased: n = 13606 for 1000 genes), blue dots (Decreased: n = 551 for 1000 genes), and grey dots (no change: n = 40175 for 1000 genes) were those sites that had increased probability (> 0.1), decreased probability (< -0.1), or not change probability (|ΔProbability| <= 0.1) respectively by introns deletion.

c, Positional plot of ΔProbability for the RAC sites located in the last 500 nt region of internal exons. Red dots (Increased: n = 13630 for 1000 genes), blue dots (Decreased: n = 559 for 1000 genes), and grey dots (no change: n = 40026 for 1000 genes) were those sites that had increased probability (> 0.1), decreased probability (< -0.1), or not change probability (|ΔProbability| <= 0.1) respectively by introns deletion.

d-e, Positional plot of ΔProbability for the RAC sites located in the first (Supplementary Fig. 4d) or last (Supplementary Fig. 4e) 200 nt region of internal exons (length: <= 200 nt). Red dots (Increased: n = 10827 for 1000 genes), blue dots (Decreased: n = 12 for 1000 genes), and grey dots (no change: n = 28366 for 1000 genes) were those sites that had increased probability (> 0.1), decreased probability (< -0.1), or not change probability (|ΔProbability| <= 0.1) respectively by introns deletion.

f-g, Positional plot of ΔProbability for the RAC sites located in the first (Supplementary Fig. 4f) or last (Supplementary Fig. 4g) 400 nt region of internal exons (length: > 200 nt & < 400 nt). Red dots (Increased: n = 4140 for 1000 genes), blue dots (Decreased: n = 123 for 1000 genes), and grey dots (no change: n = 15356 for 1000 genes) were those sites that had increased probability (> 0.1), decreased probability (< -0.1), or not change probability (|ΔProbability| <= 0.1) respectively by introns deletion.

h, Positional plot of ΔProbability for the RAC sites located in the first 500 nt region of internal exons (length: >= 400 nt). Red dots (Increased: n = 2840 for 2000 genes), blue dots (Decreased: n = 5263 for 2000 genes), and grey dots (no change: n = 36387 for 2000 genes) were those sites that had increased probability (> 0.1), decreased probability (< -0.1), or not change probability (|ΔProbability| <= 0.1) respectively by introns deletion.

i, Positional plot of ΔProbability for the RAC sites located in the last 500 nt region of internal exons (length: >= 400 nt). Red dots (Increased: n = 3599 for 2000 genes), blue dots (Decreased: n = 5128 for 2000 genes), and grey dots (no change: n = 34792 for 2000 genes) were those sites that had increased probability (> 0.1), decreased probability (< -0.1), or not change probability (|ΔProbability| <= 0.1) respectively by introns deletion.

j-k, Positional plot for the frequency of top 50 enhancers (Supplementary Fig. 4j), silencers (Supplementary Fig. 4k) in mRNA sequences around the RAC sites. The sites were located in internal exons, and the plots were compared between the increase sites (red line, ΔProbability > 0.1) and no change sites (grey line, |ΔProbability| <= 0.1).

l-m, Box plot of PhyloP score of latent m^6A sites or no change sites in near splice sites (Supplementary Fig. 4l) or away (Supplementary Fig. 4m) from internal exon ends (n = 185634 or 501206 for Supplementary Fig. 4l, n = 7196 or 103827 for Supplementary Fig. 4m). The box represents the 1st to 3rd quartile with the median marked by a horizontal line. The P-values were calculated by two-sided Wilcoxon test ($p < 2.22e^{-16}$ for Supplementary Fig. 4l,m. Significance: *** for p < 0.001).

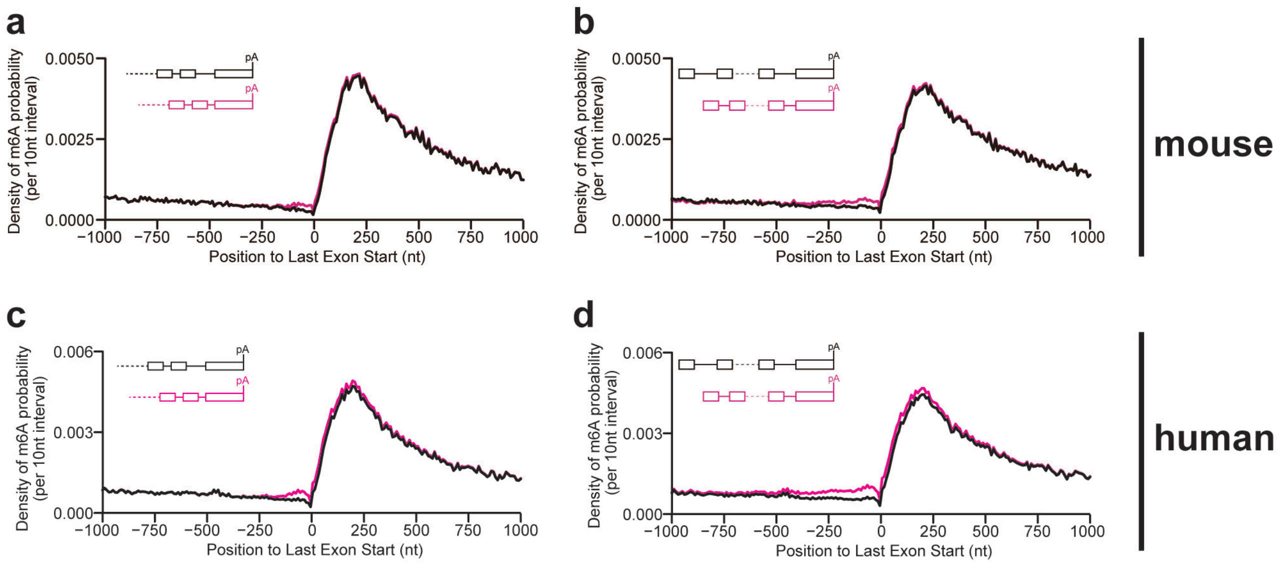
a
b
c
d
mouse
human
pA
Density of m6A probability (per 10nt interval)
Position to Last Exon Start (nt)
0.0000
0.0025
0.0050
0.000
0.003
0.006
−1000 −750 −500 −250 0 250 500 750 1000

Supplementary Fig. 5: Deep intronic sequence contributed little to the m^6A deposition inhibition

a-b, The m^6A density of mouse transcripts around last exon start were compared between full length (black line) and intron truncation (pink line) (Supplementary Fig. 5a for last intron truncation, Supplementary Fig. 5b for all introns truncation). The sequences of introns (> 400 nt) were truncated to 400 nucleotides by keeping 200 nucleotides of intron start and intron end.

c-d, The m^6A density of human transcripts around last exon start were compared between full length (black line) and intron truncation (pink line) (Supplementary Fig. 5c for last intron truncation, Supplementary Fig. 5d for all introns truncation). The sequences of introns (> 400 nt) were truncated to 400 nucleotides by keeping 200 nucleotides of intron start and intron end.

Supplementary Fig6

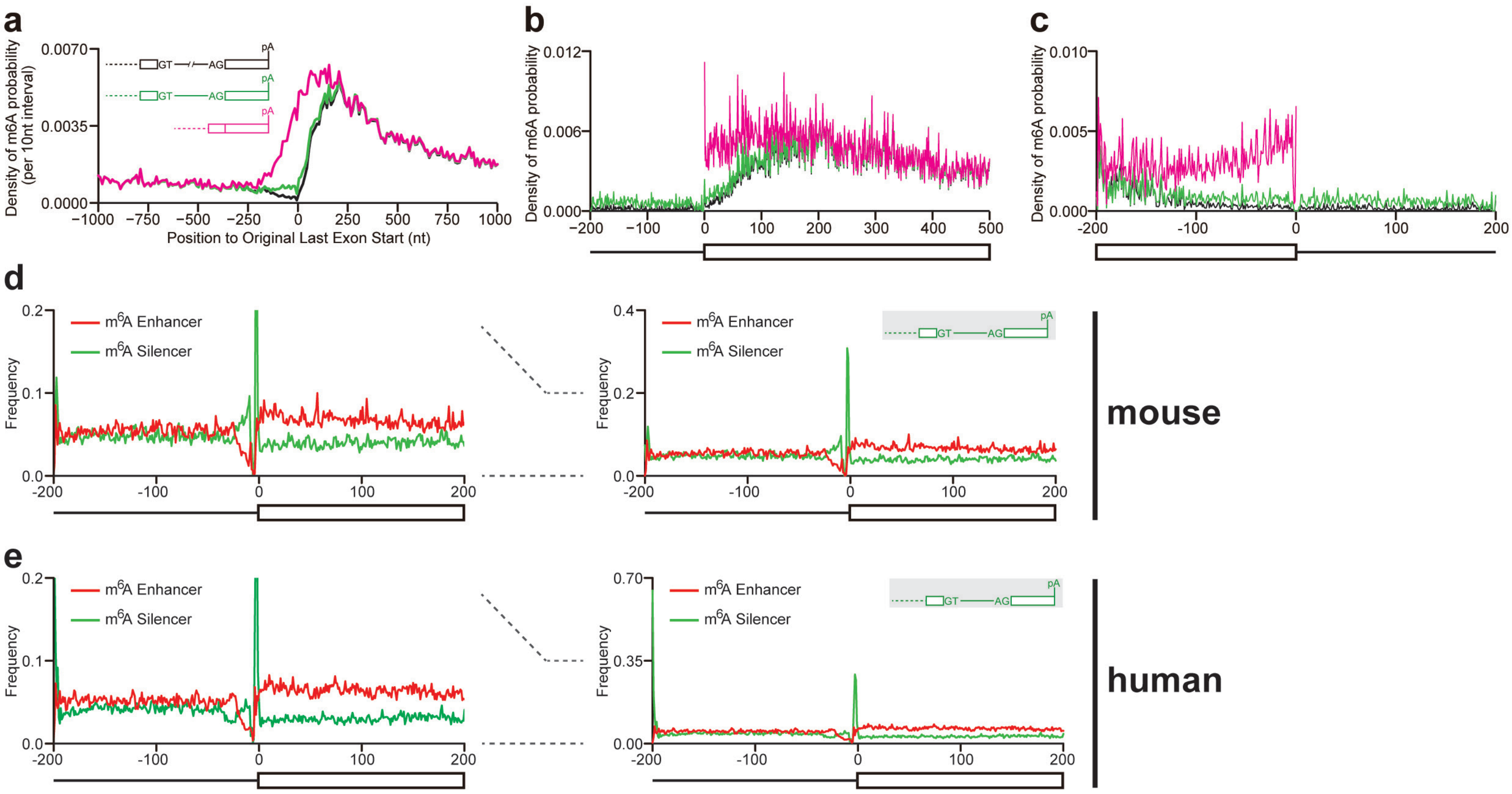

Supplementary Fig. 6: Modeling m^6A deposition in pre-mRNA with mini last intron

a, The m^6A density (10 nt interval) around last exon start were compared among the full length transcripts (black line) and their last intron truncated to 200 nt control (green line) and last intron deletion control (pink line).

b-c, The m^6A density around last exon start (Supplementary Fig. 6b) or second-to-last exon end (Supplementary Fig. 6c) were compared among the full length transcripts (black line) and their last intron truncated to 200 nt control (green line) and last intron deletion control (pink line).

d-e, Positional plot for the frequency of top 50 enhancers (red line) or silencers (green line) in the sequences of the mini-intron truncated to 200nt (by keeping 100 nucleotides of intron start and intron end) and last exon (Supplementary. Fig. 6d for mouse, and Supplementary. Fig. 6e for human).

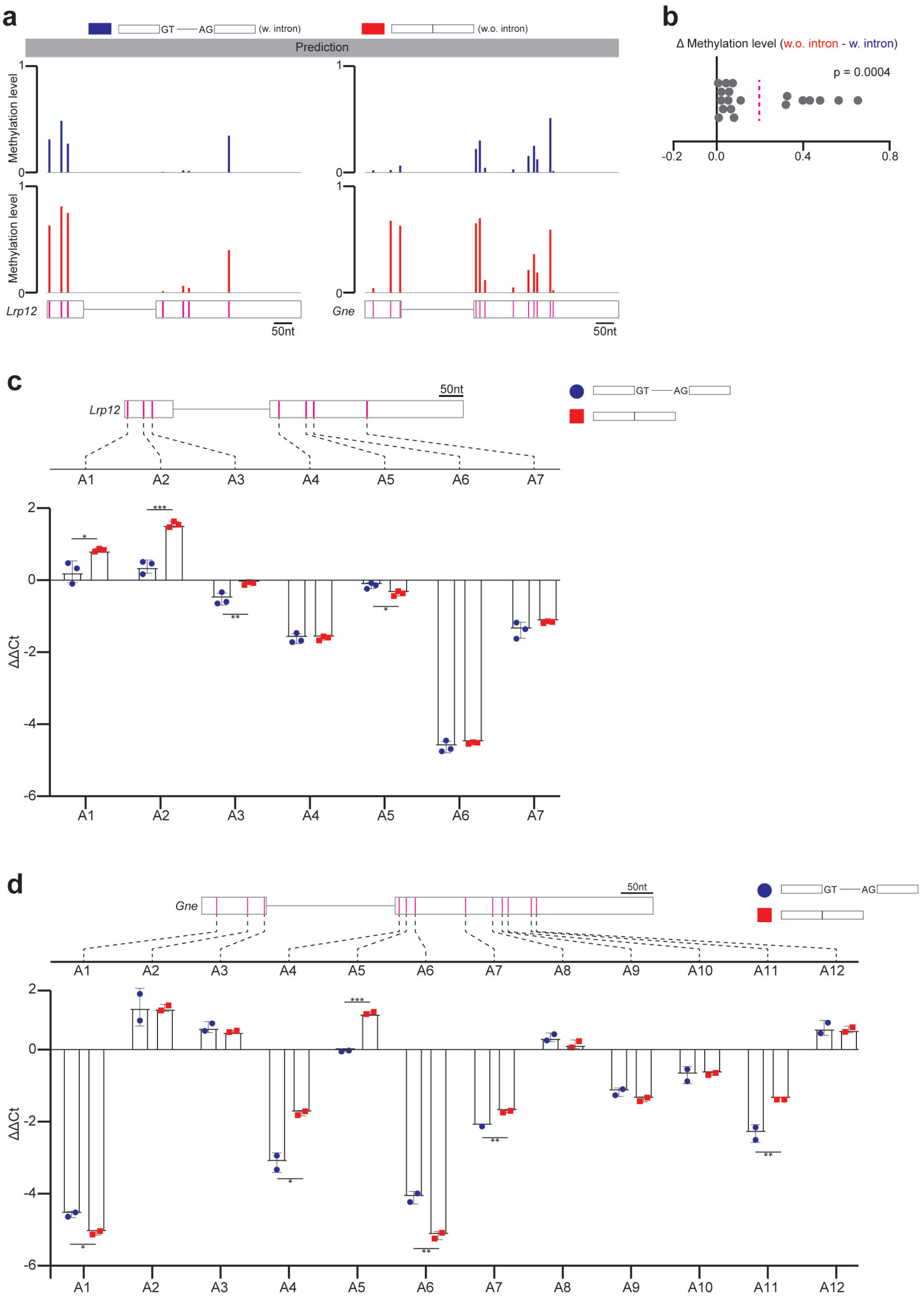
a
GT AG (w. intron)
(w.o. intron)
Prediction
Methylation level
Lrp12
Gne
50nt
b
Δ Methylation level (w.o. intron - w. intron)
p = 0.0004
-0.2
0.0
0.4
0.8
c
Lrp12
50nt
A1
A2
A3
A4
A5
A6
A7
ΔΔCt
d
Gne
50nt
A8
A9
A10
A11
A12

Supplementary Fig. 7: Experimental validation of intron repression on m^{6}A deposition

a, iM6A modeled methylation level (deposition probability) for each RAC site in minigene mRNA (left panel for *Lrp12*, and right panel for *Gne*). The m^{6}A level for constructs of intron-containing, and intron-deletion were marked by black and red colors. Each RAC site in mRNA was labeled as pink line.

b, The dot plot of the iM6A modeled ΔMethylation level for each RAC site in mRNA. ΔMethylation level was calculated for the m^{6}A signal difference of RAC sites between intron-containing and intron-deletion mRNAs. The mean value (0.197) was shown as the dotted pink line, and P-value was calculated by one-sample one-sided *t*-test for m^{6}A signal increase vs. no change.

c-d, The bar plot of qPCR ΔΔCt showing SELECT results for detecting the m^{6}A sites in mRNA. The constructs of minigenes were shown, and RAC sites in *Lrp12* (Supplementary Fig. 7c) or *Gne* (Supplementary Fig. 7d) were marked as pink line. Data were presented as mean ± SD, the P-values were calculated by two-sided Student's *t*-test ($p = 0.013, 0.00027, 0.006, 0.011$ for A1, A2, A3, A5 of *Lrp12*, $p = 0.011, 0.01, 0.0005, 0.009, 0.002, 0.02$ for A1, A4, A5, A6, A7, A11 of *Gne*. Significance: *** $p < 0.001$, ** $p < 0.01$, * $p < 0.05$. n = 3 or 2 biological independent samples for Supplementary Fig. 7c or Supplementary Fig. 7d).

Supplementary Fig8

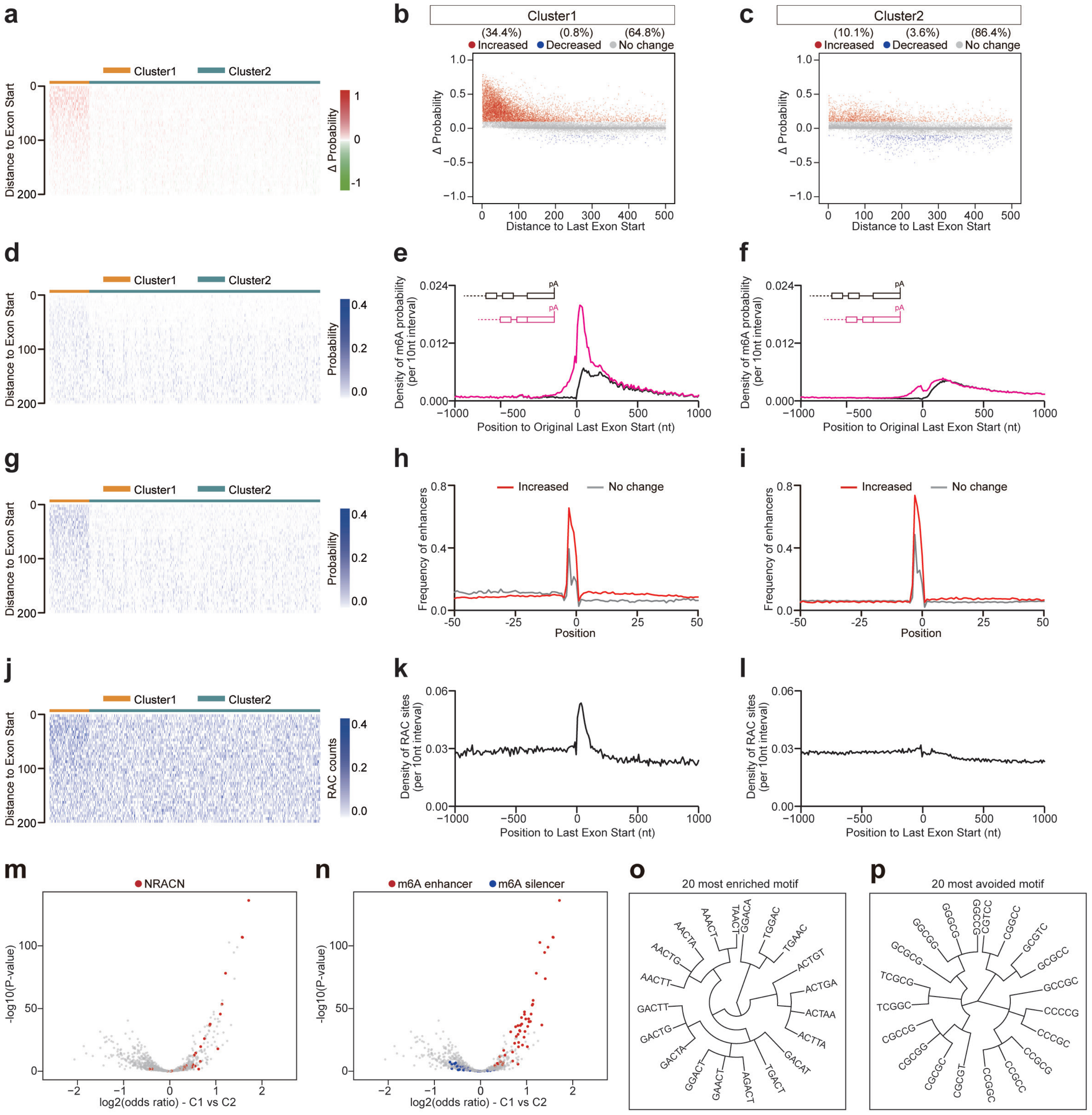

Supplementary Fig. 8: A proportion of last exons exhibit strong m^{6}A deposition inhibition by exon-intron boundary (human data).

a,d,g,j, The heatmap visualized ΔProbability (Supplementary Fig. 8a), m^{6}A Probability (Supplementary Fig. 8d), m^{6}A Probability by last intron deletion Supplementary Fig. 8g), and counts of RAC sites (Supplementary Fig. 8j) in the first 200 nt of last exon. The 200 nt was binned into 40 intervals (5 nt per interval). Genes were clustered (see details in Methods) into two clusters (Cluster1, Cluster2) based on ΔProbability.

b,c, Positional plot of ΔProbability (Supplementary Fig. 8b for Cluster1, Supplementary Fig. 8c for Cluster2) for the RAC sites located in the first 500 nt region of last exons. Red dots (Increased: n = 7378 for 1500 genes of Cluster1, n = 1766 for 1500 genes of Cluster2), blue dots (Decreased: n = 166 for 1500 genes of Cluster1, n = 601 for 1500 genes of Cluster2), and grey dots (no change: n = 13891 for 1500 genes of Cluster1, n = 15042 for 1500 genes of Cluster2) were those sites that had increased probability (> 0.1), decreased probability (< -0.1), or not change probability (|ΔProbability| <= 0.1) respectively by last intron deletion.

e,f, The m^{6}A density around original last exon start (Supplementary Fig. 8e for Cluster1, Supplementary Fig. 8f for Cluster2) were compared between the full length transcripts (black line) and the last intron deletion control (pink line). The value was calculated as the total probability value in a 10-nt interval divided by the total number of mRNAs in the interval.

h,i, Positional plot for the frequency of top 50 m^{6}A enhancers (Supplementary Fig. 8h for Cluster1, Supplementary Fig. 8i for Cluster2) in mRNA sequences around the RAC sites. The sites were in last exons, and the plots were compared between the increased sites (red line, ΔProbability > 0.1) and no change sites (grey line, |ΔProbability| <= 0.1).

k,l, The density of RAC sites around last exon start (Supplementary Fig. 8k for Cluster1, Supplementary Fig. 8l for Cluster2). The value was calculated as the total number of RAC sites in a 10-nt interval divided by the total number of

mRNAs in the interval.

m, Pentamer enrichment in different last exon start regions comparing Cluster1 vs. Cluster2. The y-axis showed the -log10(two-sided Fisher-exact test P-value), and the x-axis indicated the log2(odds ratio values). NRACN motifs were highlighted in red.

n, Pentamer enrichment in different last exon start regions comparing Cluster1 vs. Cluster2. The y-axis showed the -log10(two-sided Fisher-exact test P-value), and the x-axis indicated the log2(odds ratio values). Top 50 m^6A enhancers were highlighted in red, and top 50 m^6A silencers were highlighted in blue.

o,p, Dendrogram showed clustering of 20 most enriched (Supplementary Fig. 8o) or avoided (Supplementary Fig. 8p) motifs comparing Cluster1 vs. Cluster2.

Supplementary Fig9

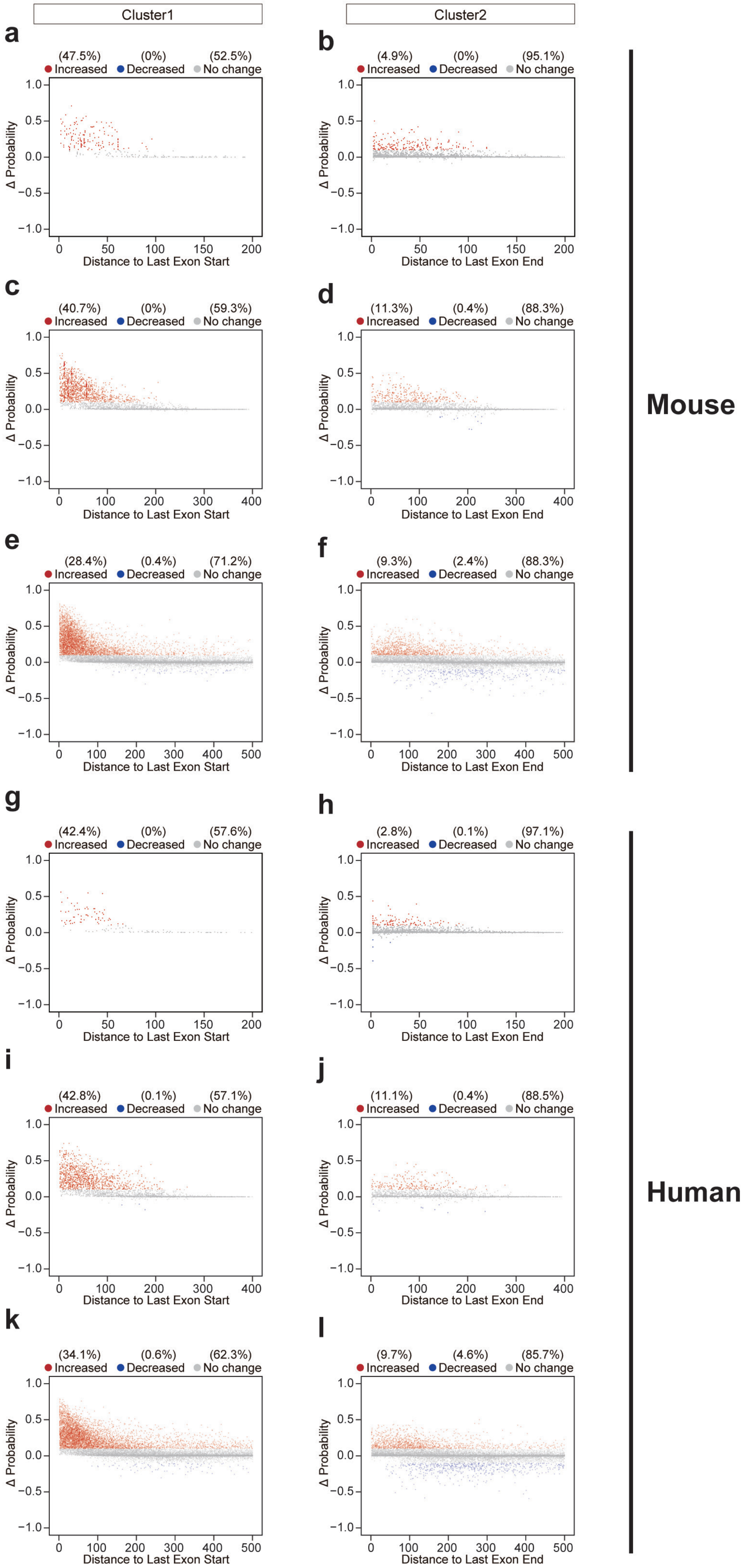

Supplementary Fig. 9: Positional plot of ΔProbability for RAC sites located in last exons

a-b, Positional plot of ΔProbability (Supplementary Fig. 9a for Cluster1, Supplementary Fig. 9b for Cluster2) for the RAC sites located in the first 200 nt region of mouse last exons (length <= 200 nt). Red dots (Increased: n = 181 for 52 genes of Cluster1, n = 199 for 933 genes of Cluster2), blue dots (Decreased: n = 0 for 52 genes of Cluster1, n = 0 for 933 genes of Cluster2), and grey dots (no change: n = 200 for 52 genes of Cluster1, n = 3897 for 933 genes of Cluster2) were those sites that had increased probability (> 0.1), decreased probability (< -0.1), or not change probability (|ΔProbability| <= 0.1) respectively by last intron deletion.

c-d, Positional plot of ΔProbability (Supplementary Fig. 9c for Cluster1, Supplementary Fig. 9d for Cluster2) for the RAC sites located in the first 400 nt region of mouse last exons (length: > 200 nt & < 400 nt). Red dots (Increased: n = 1326 for 344 genes of Cluster1, n = 306 for 344 genes of Cluster2), blue dots (Decreased: n = 0 for 344 genes of Cluster1, n = 11 for 344 genes of Cluster2), and grey dots (no change: n = 1929 for 344 genes of Cluster1, n = 2394 for 344 genes of Cluster2) were those sites that had increased probability (> 0.1), decreased probability (< -0.1), or not change probability (|ΔProbability| <= 0.1) respectively by last intron deletion.

e-f, Positional plot of ΔProbability (Supplementary Fig. 9e for Cluster1, Supplementary Fig. 9f for Cluster2) for the RAC sites located in the first 500 nt region of mouse last exons (length: >= 400 nt). Red dots (Increased: n = 4399 for 1000 genes of Cluster1, n = 1259 for 1000 genes of Cluster2), blue dots (Decreased: n = 61 for 1000 genes of Cluster1, n = 331 for 1000 genes of Cluster2), and grey dots (no change: n = 10890 for 1000 genes of Cluster1, n = 12012 for 1000 genes of Cluster2) were those sites that had increased probability (> 0.1), decreased probability (< -0.1), or not change probability (|ΔProbability| <= 0.1) respectively by last intron deletion.

g-h, Positional plot of ΔProbability (Supplementary Fig. 9g for Cluster1,

Supplementary Fig. 9h for Cluster2) for the RAC sites located in the first 200 nt region of human last exons (length <= 200 nt). Red dots (Increased: n = 61 for 19 genes of Cluster1, n = 100 for 824 genes of Cluster2), blue dots (Decreased: n = 0 for 19 genes of Cluster1, n = 4 for 824 genes of Cluster2), and grey dots (no change: n = 83 for 19 genes of Cluster1, n = 3406 for 824 genes of Cluster2) were those sites that had increased probability (> 0.1), decreased probability (< -0.1), or not change probability (|ΔProbability| <= 0.1) respectively by last intron deletion.

i-j, Positional plot of ΔProbability (Supplementary Fig. 9i for Cluster1, Supplementary Fig. 9j for Cluster2) for the RAC sites located in the first 400 nt region of human last exons (length: > 200 nt & < 400 nt). Red dots (Increased: n = 1138 for 265 genes of Cluster1, n = 223 for 265 genes of Cluster2), blue dots (Decreased: n = 3 for 265 genes of Cluster1, n = 8 for 265 genes of Cluster2), and grey dots (no change: n = 1520 for 265 genes of Cluster1, n = 1776 for 265 genes of Cluster2) were those sites that had increased probability (> 0.1), decreased probability (< -0.1), or not change probability (|ΔProbability| <= 0.1) respectively by last intron deletion.

k-l, Positional plot of ΔProbability (Supplementary Fig. 9k for Cluster1, Supplementary Fig. 9l for Cluster2) for the RAC sites located in the first 500 nt region of human last exons (length: >= 400 nt). Red dots (Increased: n = 5068 for 1000 genes of Cluster1, n = 1251 for 1000 genes of Cluster2), blue dots (Decreased: n = 92 for 1000 genes of Cluster1, n = 593 for 1000 genes of Cluster2), and grey dots (no change: n = 9708 for 1000 genes of Cluster1, n = 11038 for 1000 genes of Cluster2) were those sites that had increased probability (> 0.1), decreased probability (< -0.1), or not change probability (|ΔProbability| <= 0.1) respectively by last intron deletion.

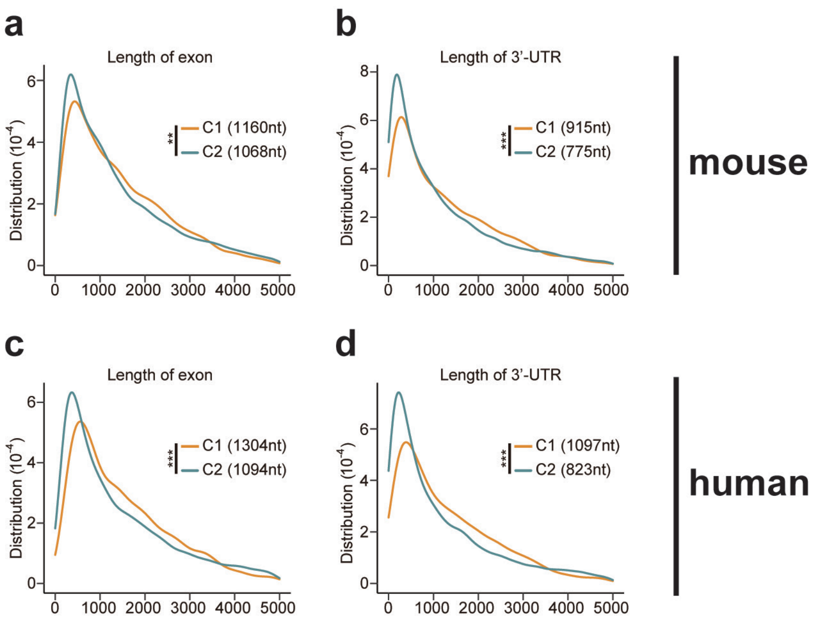
a
Length of exon
Distribution (10⁻⁴)
**
C1 (1160nt)
C2 (1068nt)
b
Length of 3'-UTR
Distribution (10⁻⁴)
***
C1 (915nt)
C2 (775nt)
mouse
c
Length of exon
Distribution (10⁻⁴)
***
C1 (1304nt)
C2 (1094nt)
d
Length of 3'-UTR
Distribution (10⁻⁴)
***
C1 (1097nt)
C2 (823nt)
human

Supplementary Fig. 10: Density plot of last exon length and 3'-UTR length

a-d, Density plot of exon length (Supplementary Fig. 10a for mouse, and Supplementary Fig. 10c for human), and 3'-UTR length (Supplementary Fig. 10b for mouse, and Supplementary Fig. 10d for human) of last exons. The density was compared between Cluster1 and Cluster2. The P-values were calculated by the two-sided Kolmogorov–Smirnov test ($p = 0.003$ for Supplementary Fig. 10a, $p = 1.8e^{-7}$ for Supplementary Fig. 10b, $p < 2.2e^{-16}$ for Supplementary Fig. 10c, $p < 2.2e^{-16}$ for Supplementary Fig. 10d. Significance: *** $p < 0.001$, ** $p < 0.01$). The median values were labeled.

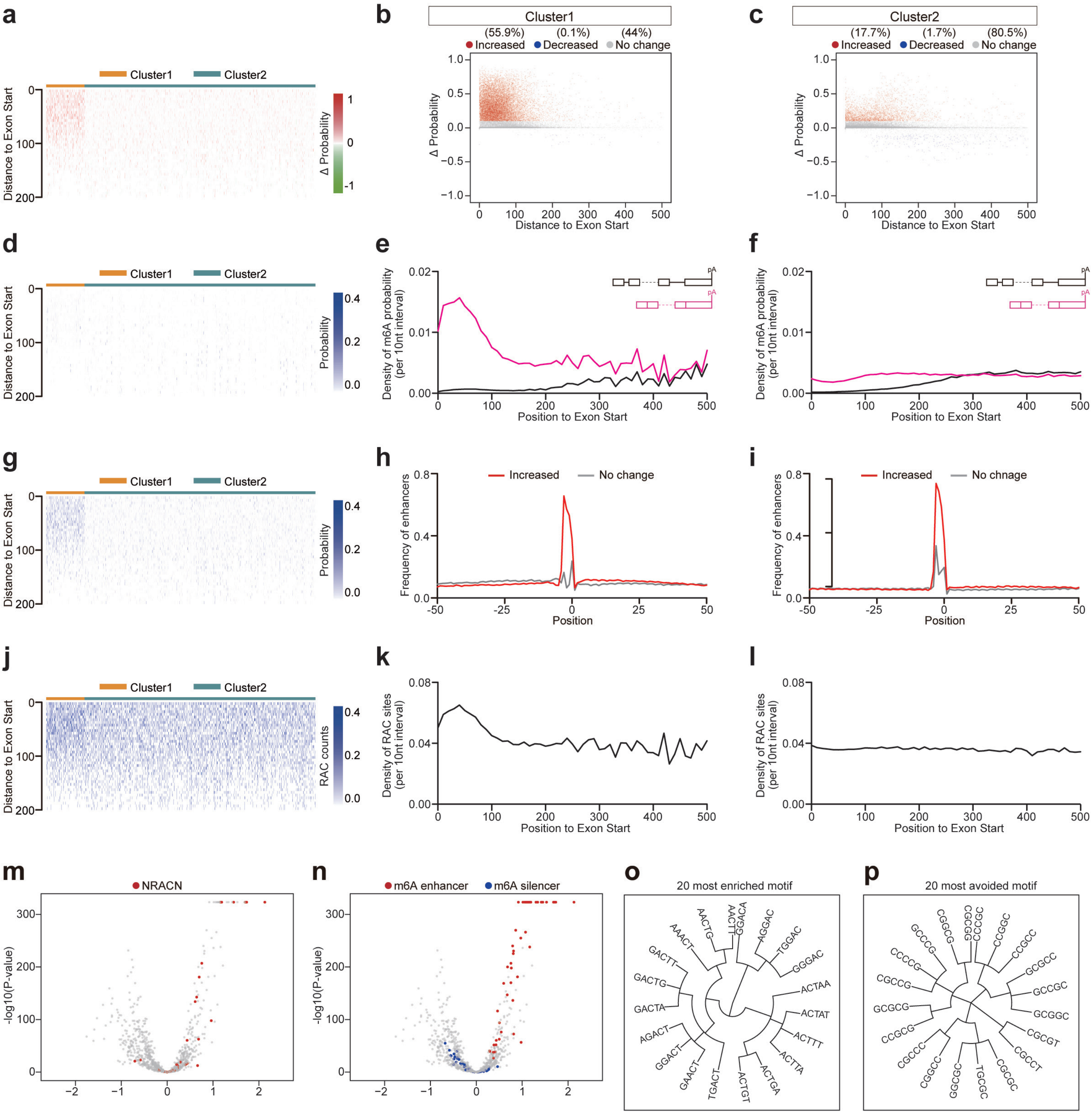
a
Cluster1
Cluster2
Distance to Exon Start
Δ Probability
b
Cluster1
(55.9%) Increased
(0.1%) Decreased
(44%) No change
Δ Probability
Distance to Exon Start
c
Cluster2
(17.7%) Increased
(1.7%) Decreased
(80.5%) No change
d
Probability
e
Density of m6A probability (per 10nt interval)
Position to Exon Start
pA
f
g
h
Increased
No change
Frequency of enhancers
Position
i
No chnage
j
RAC counts
k
Density of RAC sites (per 10nt interval)
l
m
NRACN
-log10(P-value)
n
m6A enhancer
m6A silencer
o
20 most enriched motif
p
20 most avoided motif

Supplementary Fig. 11: A proportion of internal exons exhibit strong m^{6}A deposition inhibition by exon-intron boundary (human data).

a,d,g,j, The heatmap visualized ΔProbability (Supplementary Fig. 11a), m^{6}A Probability (Supplementary Fig. 11d), m^{6}A Probability by introns deletion (Supplementary Fig. 11g), and counts of RAC sites (Supplementary Fig. 11j) in the first 200 nt of internal exon. The 200 nt was binned into 40 intervals (5 nt per interval). Exons were clustered (see details in Methods) into two clusters (Cluster1, Cluster2) based on ΔProbability.

b,c, Positional plot of ΔProbability (Supplementary Fig. 11b for Cluster1, Supplementary Fig. 11c for Cluster2) for the RAC sites located in the first 500 nt region of internal exons. Red dots (Increased: n = 11430 for 3000 exons of Cluster1, n = 2776 for 3000 exons of Cluster2), blue dots (Decreased: n = 26 for 3000 exons of Cluster1, n = 269 for 3000 exons of Cluster2), and grey dots (no change: n = 8995 for 3000 exons of Cluster1, n = 12606 for 3000 exons of Cluster2) were those sites that had increased probability (> 0.1), decreased probability (< -0.1), or not change probability (|ΔProbability| <= 0.1) respectively by introns deletion.

e,f, The m^{6}A density at internal exon start (Supplementary Fig. 11e for Cluster1, Supplementary Fig. 11f for Cluster2) were compared between the full length transcripts (black line) and the introns deletion control (pink line). The value was calculated as the total probability value in a 10-nt interval divided by the total number of mRNAs in the interval.

h,i, Positional plot for the frequency of top 50 m^{6}A enhancers (Supplementary Fig. 11h for Cluster1, Supplementary Fig. 11i for Cluster2) in mRNA sequences around the RAC sites. The sites were in internal exons, and the plots were compared between the increased sites (red line, ΔProbability > 0.1) and no change sites (grey line, |ΔProbability| <= 0.1).

k,l, The density of RAC sites at internal exon start (Supplementary Fig. 11k for Cluster1, Supplementary Fig. 11l for Cluster2). The value was calculated as the total number of RAC sites in a 10-nt interval divided by the total number of

mRNAs in the interval.

m, Pentamer enrichment in different last exon start regions comparing Cluster1 vs. Cluster2. The y-axis showed the -log10(two-sided Fisher-exact test P-value), and the x-axis indicated the log2(odds ratio values). NRACN motifs were highlighted in red.

n, Pentamer enrichment in different internal exon start regions comparing Cluster1 vs. Cluster2. The y-axis showed the -log10(two-sided Fisher-exact test P-value), and the x-axis indicated the log2(odds ratio values). Top 50 m^6A enhancers were highlighted in red, and top 50 m^6A silencers were highlighted in blue.

o,p, Dendrogram showed clustering of 20 most enriched (Supplementary Fig. 11o) or avoided (Supplementary Fig. 11p) motifs comparing Cluster1 vs. Cluster2.

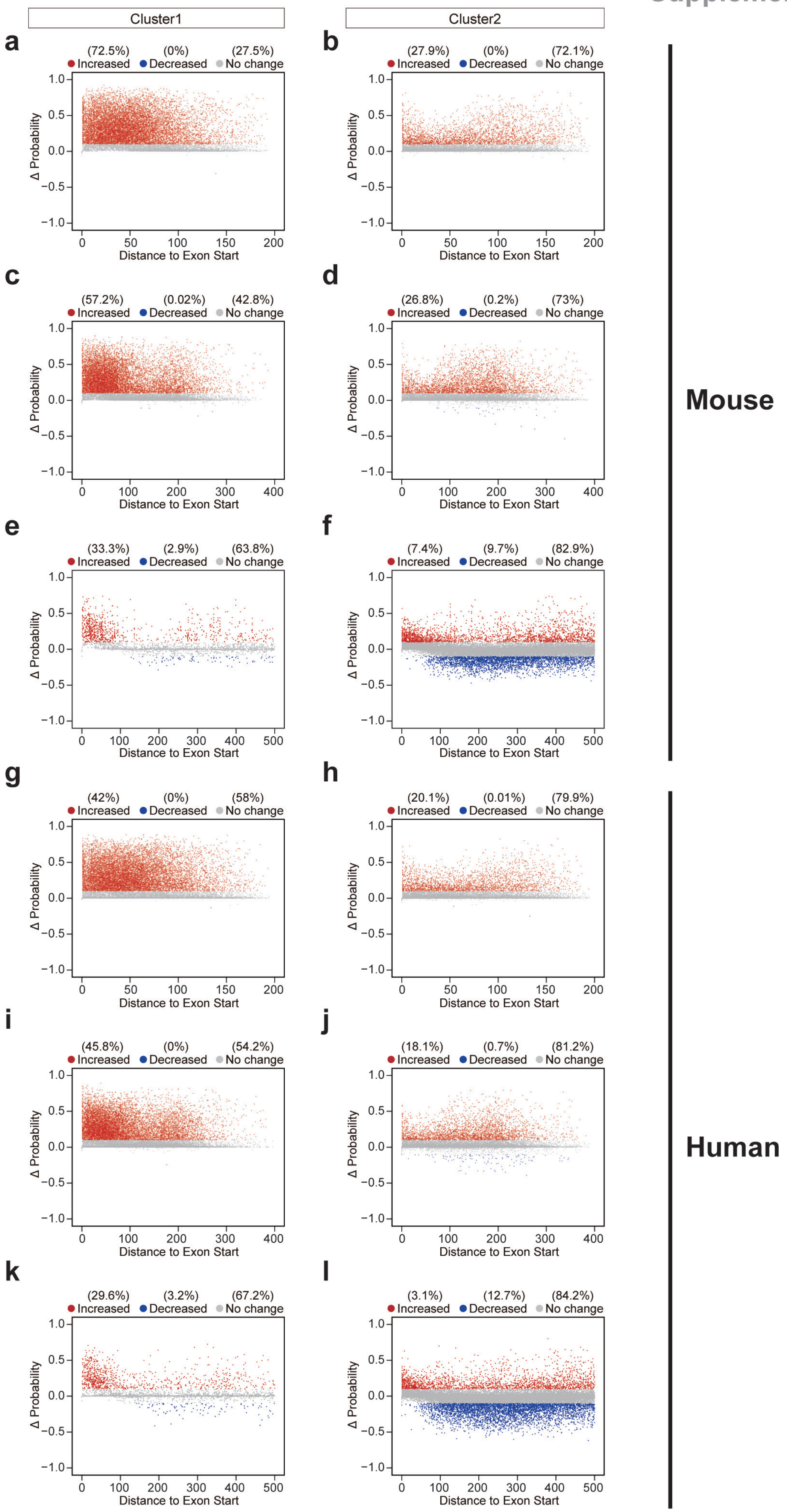
Cluster1
Cluster2
a
b
c
d
e
f
g
h
i
j
k
l
(72.5%) Increased (0%) Decreased (27.5%) No change
(27.9%) Increased (0%) Decreased (72.1%) No change
(57.2%) Increased (0.02%) Decreased (42.8%) No change
(26.8%) Increased (0.2%) Decreased (73%) No change
(33.3%) Increased (2.9%) Decreased (63.8%) No change
(7.4%) Increased (9.7%) Decreased (82.9%) No change
(42%) Increased (0%) Decreased (58%) No change
(20.1%) Increased (0.01%) Decreased (79.9%) No change
(45.8%) Increased (0%) Decreased (54.2%) No change
(18.1%) Increased (0.7%) Decreased (81.2%) No change
(29.6%) Increased (3.2%) Decreased (67.2%) No change
(3.1%) Increased (12.7%) Decreased (84.2%) No change
Δ Probability
Distance to Exon Start
Mouse
Human

Supplementary Fig. 12: Positional plot of ΔProbability for RAC sites located in internal exons

a-b, Positional plot of ΔProbability (Supplementary Fig. 12a for Cluster1, Supplementary Fig. 12b for Cluster2) for the RAC sites located in the first 200 nt region of mouse internal exons (length <= 200 nt). Red dots (Increased: n = 11190 for 3000 exons of Cluster1, n = 2874 for 3000 exons of Cluster2), blue dots (Decreased: n = 1 for 3000 exons of Cluster1, n = 1 for 3000 exons of Cluster2), and grey dots (no change: n = 4234 for 3000 exons of Cluster1, n = 7432 for 3000 exons of Cluster2) were those sites that had increased probability (> 0.1), decreased probability (< -0.1), or not change probability (|ΔProbability| <= 0.1) respectively by introns deletion.

c-d, Positional plot of ΔProbability (Supplementary Fig. 12c for Cluster1, Supplementary Fig. 12d for Cluster2) for the RAC sites located in the first 400 nt region of mouse internal exons (length: > 200 nt & < 400 nt). Red dots (Increased: n = 9235 for 1800 exons of Cluster1, n = 3545 for 1800 exons of Cluster2), blue dots (Decreased: n = 4 for 1800 exons of Cluster1, n = 30 for 1800 exons of Cluster2), and grey dots (no change: n = 6905 for 1800 exons of Cluster1, n = 9651 for 1800 exons of Cluster2) were those sites that had increased probability (> 0.1), decreased probability (< -0.1), or not change probability (|ΔProbability| <= 0.1) respectively by introns deletion.

e-f, Positional plot of ΔProbability (Supplementary Fig. 12e for Cluster1, Supplementary Fig. 12f for Cluster2) for the RAC sites located in the first 500 nt region of mouse internal exons (length: >= 400 nt). Red dots (Increased: n = 876 for 165 exons of Cluster1, n = 2137 for 2000 exons of Cluster2), blue dots (Decreased: n = 77 for 165 exons of Cluster1, n = 2810 for 2000 exons of Cluster2), and grey dots (no change: n = 1676 for 165 exons of Cluster1, n = 24063 for 2000 exons of Cluster2) were those sites that had increased probability (> 0.1), decreased probability (< -0.1), or not change probability (|ΔProbability| <= 0.1) respectively by introns deletion.

g-h, Positional plot of ΔProbability (Supplementary Fig. 12g for Cluster1,

Supplementary Fig. 12h for Cluster2) for the RAC sites located in the first 200 nt region of human internal exons (length <= 200 nt). Red dots (Increased: n = 11109 for 3000 exons of Cluster1, n = 2590 for 3000 exons of Cluster2), blue dots (Decreased: n = 1 for 3000 exons of Cluster1, n = 2 for 3000 exons of Cluster2), and grey dots (no change: n = 8067 for 3000 exons of Cluster1, n = 10301 for 3000 exons of Cluster2) were those sites that had increased probability (> 0.1), decreased probability (< -0.1), or not change probability (|ΔProbability| <= 0.1) respectively by introns deletion.

i-j, Positional plot of ΔProbability (Supplementary Fig. 12i for Cluster1, Supplementary Fig. 12j for Cluster2) for the RAC sites located in the first 400 nt region of human internal exons (length: > 200 nt & < 400 nt). Red dots (Increased: n = 9335 for 1800 exons of Cluster1, n = 2963 for 1800 exons of Cluster2), blue dots (Decreased: n = 1 for 1800 exons of Cluster1, n = 119 for 1800 exons of Cluster2), and grey dots (no change: n = 11050 for 1800 exons of Cluster1, n = 13292 for 1800 exons of Cluster2) were those sites that had increased probability (> 0.1), decreased probability (< -0.1), or not change probability (|ΔProbability| <= 0.1) respectively by introns deletion.

k-l, Positional plot of ΔProbability (Supplementary Fig. 12k for Cluster1, Supplementary Fig. 12l for Cluster2) for the RAC sites located in the first 500 nt region of human internal exons (length: >= 400 nt). Red dots (Increased: n = 900 for 150 exons of Cluster1, n = 1063 for 2000 exons of Cluster2), blue dots (Decreased: n = 98 for 150 exons of Cluster1, n = 4347 for 2000 exons of Cluster2), and grey dots (no change: n = 2044 for 150 exons of Cluster1, n = 28936 for 2000 exons of Cluster2) were those sites that had increased probability (> 0.1), decreased probability (< -0.1), or not change probability (|ΔProbability| <= 0.1) respectively by introns deletion.

Supplementary Fig13

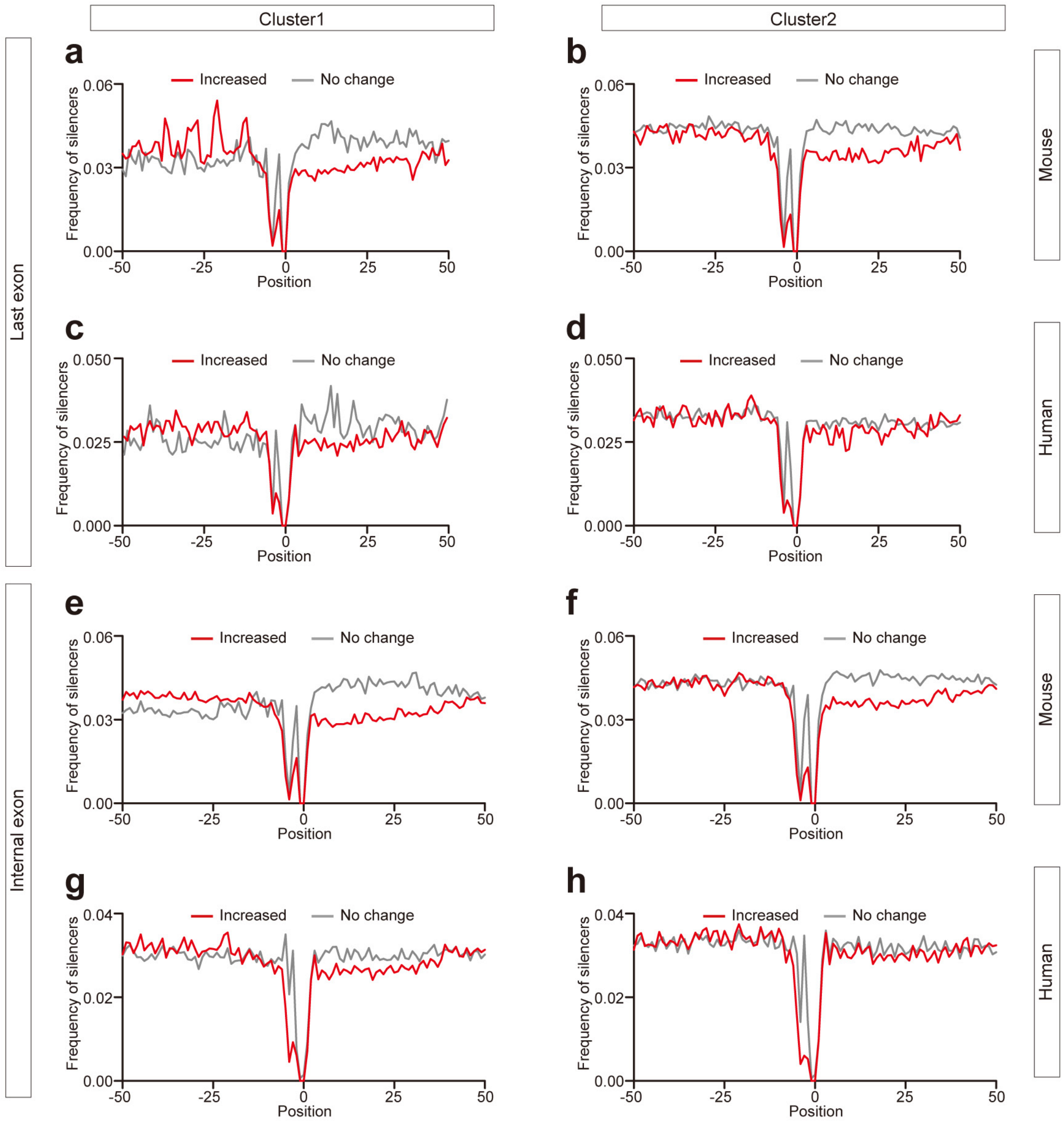

Supplementary Fig. 13: m^{6}A silencers avoids 50 nt downstream of latent sites.

a,b,c,d, Positional plot for the frequency of top 50 m^{6}A silencers (Supplementary Fig. 13a,c for Cluster1 in mouse or human, Supplementary Fig. 13b,d for Cluster2 in mouse or human) in mRNA sequences around the RAC sites. These sites were in last exons, and the plots were compared between the increased sites (red line, ΔProbability > 0.1) and no change sites (grey line, |ΔProbability| <= 0.1).

e,f,g,h, Positional plot for the frequency of top 50 m^{6}A silencers (Supplementary Fig. 13e,g for Cluster1 in mouse or human, Supplementary Fig. 13f,h for Cluster2 in mouse or human) in mRNA sequences around the RAC sites. These sites were located in internal exons, and the plots were compared between the increased sites (red line, ΔProbability > 0.1) and no change sites (grey line, |ΔProbability| <= 0.1).

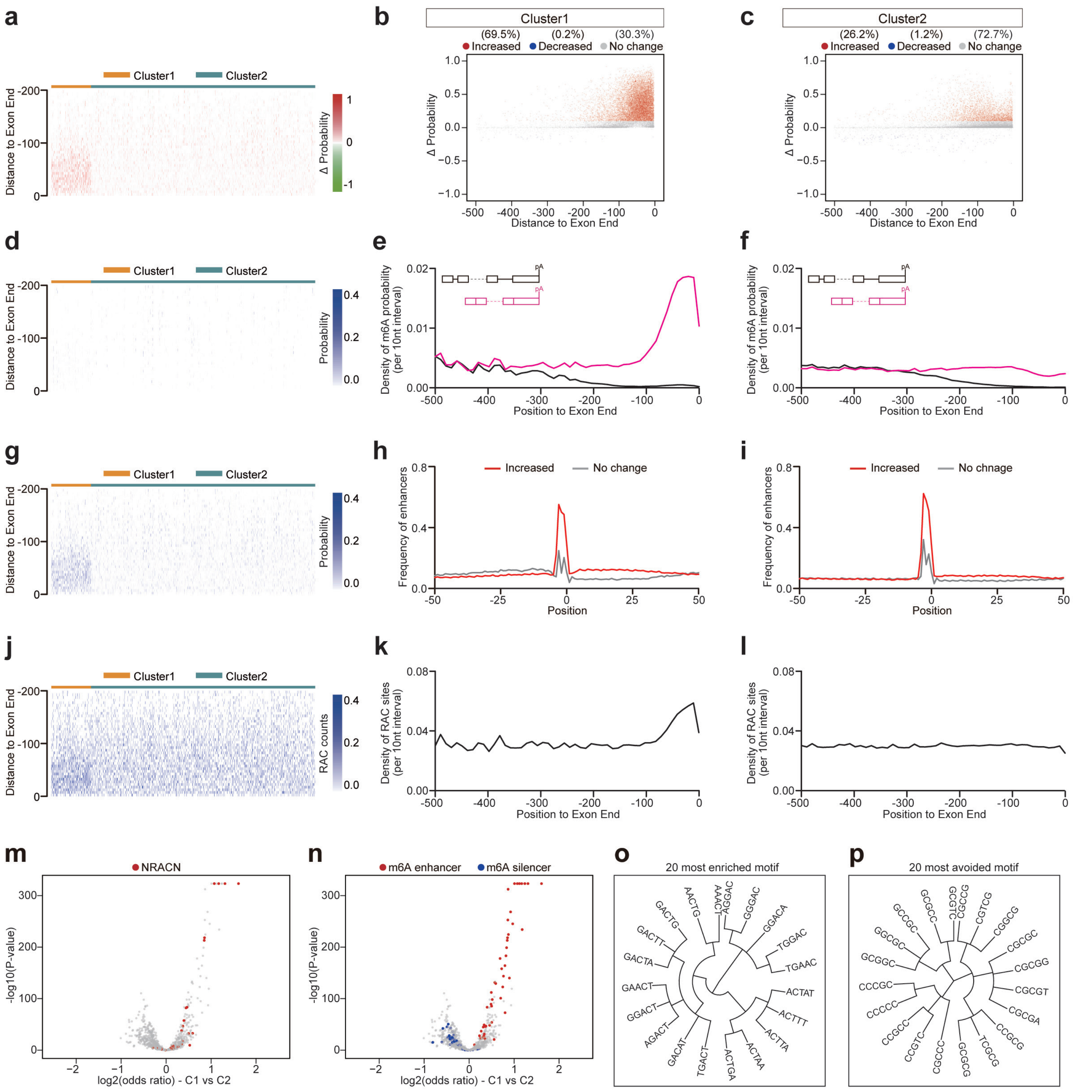
a
Cluster1
Cluster2
Distance to Exon End
Δ Probability
b
Cluster1
(69.5%) Increased
(0.2%) Decreased
(30.3%) No change
Δ Probability
Distance to Exon End
c
Cluster2
(26.2%) Increased
(1.2%) Decreased
(72.7%) No change
Δ Probability
Distance to Exon End
d
Cluster1
Cluster2
Distance to Exon End
Probability
e
Density of m6A probability (per 10nt interval)
Position to Exon End
f
Density of m6A probability (per 10nt interval)
Position to Exon End
g
Cluster1
Cluster2
Distance to Exon End
Probability
h
Increased
No change
Frequency of enhancers
Position
i
Increased
No chnage
Frequency of enhancers
Position
j
Cluster1
Cluster2
Distance to Exon End
RAC counts
k
Density of RAC sites (per 10nt interval)
Position to Exon End
l
Density of RAC sites (per 10nt interval)
Position to Exon End
m
NRACN
-log10(P-value)
log2(odds ratio) - C1 vs C2
n
m6A enhancer
m6A silencer
-log10(P-value)
log2(odds ratio) - C1 vs C2
o
20 most enriched motif
p
20 most avoided motif

Supplementary Fig. 14: A proportion of internal exons exhibit strong m^{6}A deposition inhibition by exon-intron boundary (mouse data).

a,d,g,j, The heatmap visualized ΔProbability (Supplementary Fig. 14a), m^{6}A Probability (Supplementary Fig. 14d), m^{6}A Probability by introns deletion (Supplementary Fig. 14g), and counts of RAC sites (Supplementary Fig. 14j) in the last 200 nt of internal exon. The 200 nt was binned into 40 intervals (5 nt per interval). Exons were clustered (see details in Methods) into two clusters (Cluster1, Cluster2) based on ΔProbability.

b,c, Positional plot of ΔProbability (Supplementary Fig. 14b for Cluster1, Supplementary Fig. 14c for Cluster2) for the RAC sites located in the last 500 nt region of internal exons. Red dots (Increased: n = 11538 for 3000 exons of Cluster1, n = 3299 for 3000 exons of Cluster2), blue dots (Decreased: n = 32 for 3000 exons of Cluster1, n = 147 for 3000 exons of Cluster2), and grey dots (no change: n = 5020 for 3000 exons of Cluster1, n = 9155 for 3000 exons of Cluster2) were those sites that had increased probability (> 0.1), decreased probability (< -0.1), or not change probability (|ΔProbability| <= 0.1) respectively by introns deletion.

e,f, The m^{6}A density at internal exon end (Supplementary Fig. 14e for Cluster1, Supplementary Fig. 14f for Cluster2) were compared between the full length transcripts (black line) and the introns deletion control (pink line). The value was calculated as the total probability value in a 10-nt interval divided by the total number of mRNAs in the interval.

h,i, Positional plot for the frequency of top 50 m^{6}A enhancers (Supplementary Fig. 14h for Cluster1, Supplementary Fig. 14i for Cluster2) in mRNA sequences around the RAC sites. The sites were in internal exons, and the plots were compared between the increased sites (red line, ΔProbability > 0.1) and no change sites (grey line, |ΔProbability| <= 0.1).

k,l, The density of RAC sites at internal exon end (Supplementary Fig. 14k for Cluster1, Supplementary Fig. 14l for Cluster2). The value was calculated as the total number of RAC sites in a 10-nt interval divided by the total number of

mRNAs in the interval.

m, Pentamer enrichment in different last exon start regions comparing Cluster1 vs. Cluster2. The y-axis showed the -log10(two-sided Fisher-exact test P-value), and the x-axis indicated the log2(odds ratio values). NRACN motifs were highlighted in red.

n, Pentamer enrichment in different internal exon end regions comparing Cluster1 vs. Cluster2. The y-axis showed the -log10(two-sided Fisher-exact test P-value), and the x-axis indicated the log2(odds ratio values). Top 50 m^6A enhancers were highlighted in red, and top 50 m^6A silencers were highlighted in blue.

o,p, Dendrogram showed clustering of 20 most enriched (Supplementary Fig. 14o) or avoided (Supplementary Fig. 14p) motifs comparing Cluster1 vs. Cluster2.

Supplementary Fig15

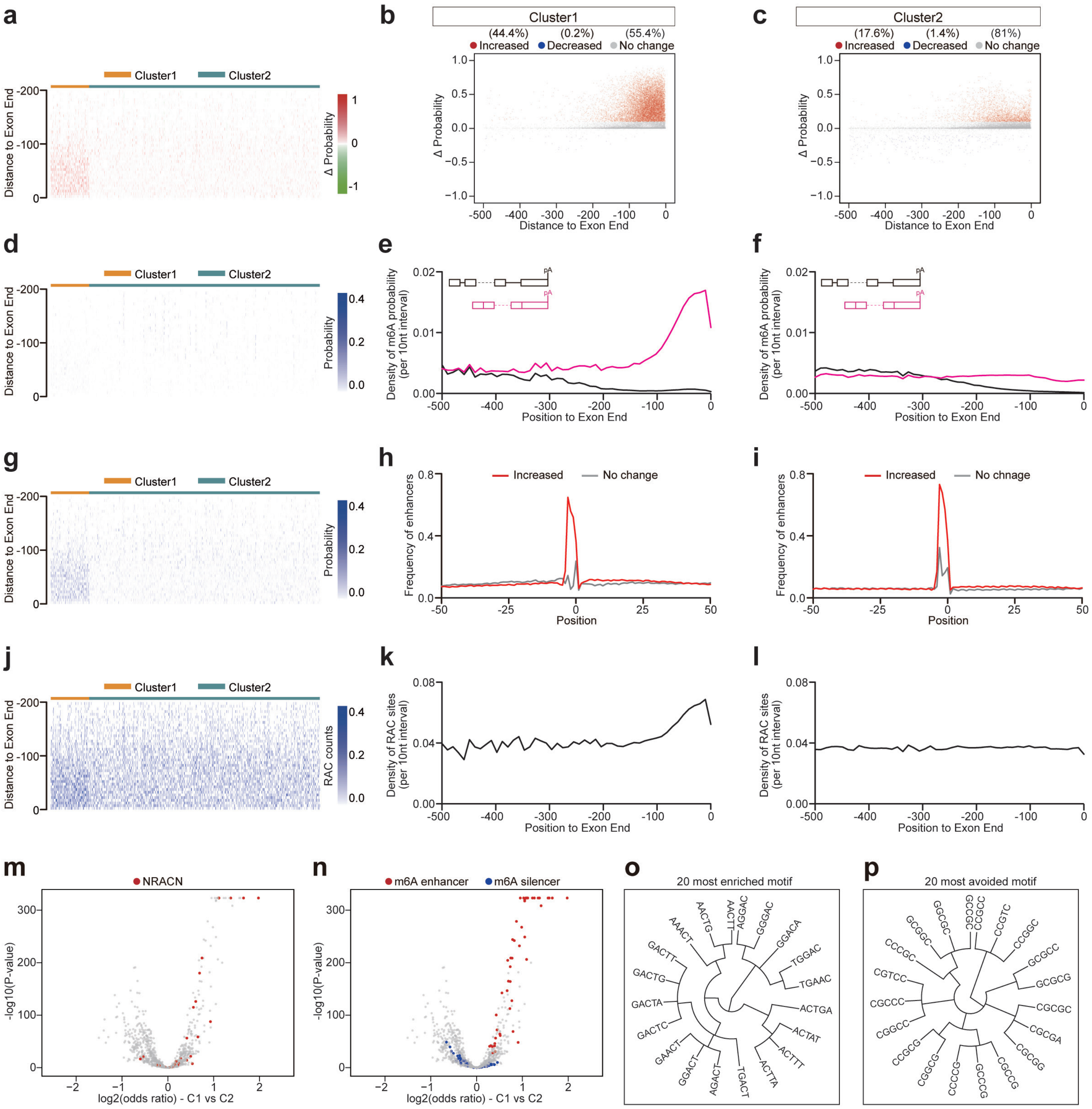

Supplementary Fig. 15: A proportion of internal exons exhibit strong m^{6}A deposition inhibition by exon-intron boundary (human data).

a,d,g,j, The heatmap visualized ΔProbability (Supplementary Fig. 15a), m^{6}A Probability (Supplementary Fig. 15d), m^{6}A Probability by introns deletion (Supplementary Fig. 15g), and counts of RAC sites (Supplementary Fig. 15j) in the last 200 nt of internal exon. The 200 nt was binned into 40 intervals (5 nt per interval). Exons were clustered (see details in Methods) into two clusters (Cluster1, Cluster2) based on ΔProbability.

b,c, Positional plot of ΔProbability (Supplementary Fig. 15b for Cluster1, Supplementary Fig. 15c for Cluster2) for the RAC sites located in the last 500 nt region of internal exons. Red dots (Increased: n = 11587 for 3000 exons of Cluster1, n = 2806 for 3000 exons of Cluster2), blue dots (Decreased: n = 34 for 3000 exons of Cluster1, n = 225 for 3000 exons of Cluster2), and grey dots (no change: n = 9258 for 3000 exons of Cluster1, n = 12902 for 3000 exons of Cluster2) were those sites that had increased probability (> 0.1), decreased probability (< -0.1), or not change probability (|ΔProbability| <= 0.1) respectively by introns deletion.

e,f, The m^{6}A density at internal exon end (Supplementary Fig. 15e for Cluster1, Supplementary Fig. 15f for Cluster2) were compared between the full length transcripts (black line) and the introns deletion control (pink line). The value was calculated as the total probability value in a 10-nt interval divided by the total number of mRNAs in the interval.

h,i, Positional plot for the frequency of top 50 m^{6}A enhancers (Supplementary Fig. 15h for Cluster1, Supplementary Fig. 15i for Cluster2) in mRNA sequences around the RAC sites. The sites were in internal exons, and the plots were compared between the increased sites (red line, ΔProbability > 0.1) and no change sites (grey line, |ΔProbability| <= 0.1).

k,l, The density of RAC sites at internal exon end (Supplementary Fig. 15k for Cluster1, Supplementary Fig. 15l for Cluster2). The value was calculated as the total number of RAC sites in a 10-nt interval divided by the total number of

mRNAs in the interval.

m, Pentamer enrichment in different last exon start regions comparing Cluster1 vs. Cluster2. The y-axis showed the -log10(two-sided Fisher-exact test P-value), and the x-axis indicated the log2(odds ratio values). NRACN motifs were highlighted in red.

n, Pentamer enrichment in different internal exon end regions comparing Cluster1 vs. Cluster2. The y-axis showed the -log10(two-sided Fisher-exact test P-value), and the x-axis indicated the log2(odds ratio values). Top 50 m^6A enhancers were highlighted in red, and top 50 m^6A silencers were highlighted in blue.

o,p, Dendrogram showed clustering of 20 most enriched (Supplementary Fig. 15o) or avoided (Supplementary Fig. 15p) motifs comparing Cluster1 vs. Cluster2.

Supplementary Fig16

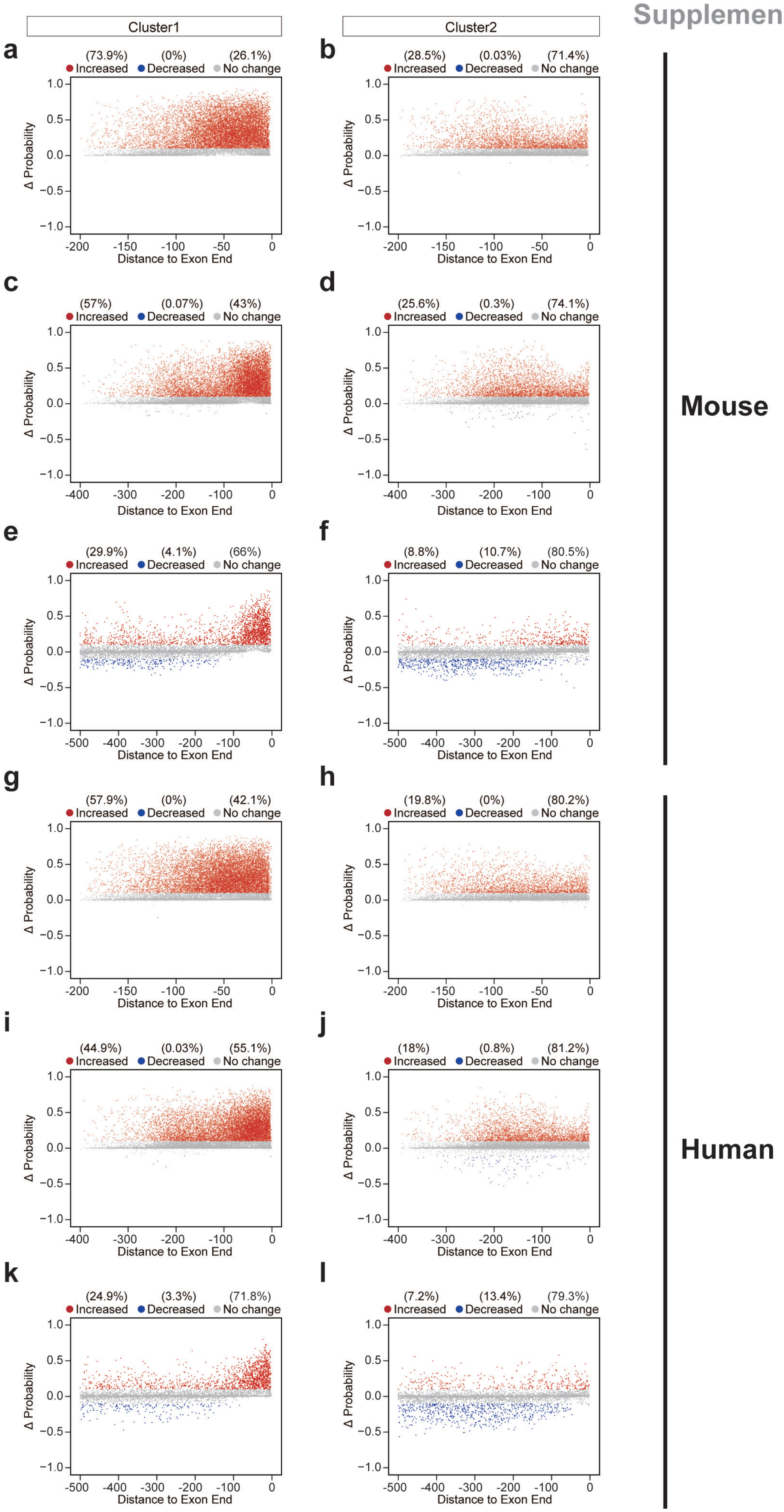

Supplementary Fig.16: Positional plot of ΔProbability for RAC sites located in internal exons.

a-b, Positional plot of ΔProbability (Supplementary Fig.16a for Cluster1, Supplementary Fig.16b for Cluster2) for the RAC sites located in the last 200 nt region of mouse internal exons (length <= 200 nt). Red dots (Increased: n = 11304 for 3000 exons of Cluster1, n = 3010 for 3000 exons of Cluster2), blue dots (Decreased: n = 0 for 3000 exons of Cluster1, n = 3 for 3000 exons of Cluster2), and grey dots (no change: n = 3907 for 3000 exons of Cluster1, n = 7537 for 3000 exons of Cluster2) were those sites that had increased probability (> 0.1), decreased probability (< -0.1), or not change probability (|ΔProbability| <= 0.1) respectively by introns deletion.

c-d, Positional plot of ΔProbability (Supplementary Fig.16c for Cluster1, Supplementary Fig.16d for Cluster2) for the RAC sites located in the last 400 nt region of mouse internal exons (length: > 200 nt & < 400 nt). Red dots (Increased: n = 9000 for 1800 exons of Cluster1, n = 3347 for 1800 exons of Cluster2), blue dots (Decreased: n = 11 for 1800 exons of Cluster1, n = 40 for 1800 exons of Cluster2), and grey dots (no change: n = 6789 for 1800 exons of Cluster1, n = 9700 for 1800 exons of Cluster2) were those sites that had increased probability (> 0.1), decreased probability (< -0.1), or not change probability (|ΔProbability| <= 0.1) respectively by introns deletion.

e-f, Positional plot of ΔProbability (Supplementary Fig.16e for Cluster1, Supplementary Fig.16f for Cluster2) for the RAC sites located in the last 500 nt region of mouse internal exons (length: >= 400 nt). Red dots (Increased: n = 1929 for 400 exons of Cluster1, n = 485 for 400 exons of Cluster2), blue dots (Decreased: n = 265 for 400 exons of Cluster1, n = 595 for 400 exons of Cluster2), and grey dots (no change: n = 4257 for 400 exons of Cluster1, n = 4457 for 400 exons of Cluster2) were those sites that had increased probability (> 0.1), decreased probability (< -0.1), or not change probability (|ΔProbability| <= 0.1) respectively by introns deletion.

g-h, Positional plot of ΔProbability (Supplementary Fig.16g for Cluster1,

Supplementary Fig.16h for Cluster2) for the RAC sites located in the last 200 nt region of human internal exons (length <= 200 nt). Red dots (Increased: n = 11067 for 3000 exons of Cluster1, n = 2550 for 3000 exons of Cluster2), blue dots (Decreased: n = 1 for 3000 exons of Cluster1, n = 1 for 3000 exons of Cluster2), and grey dots (no change: n = 8039 for 3000 exons of Cluster1, n = 10310 for 3000 exons of Cluster2) were those sites that had increased probability (> 0.1), decreased probability (< -0.1), or not change probability (|ΔProbability| <= 0.1) respectively by introns deletion.

i-j, Positional plot of ΔProbability (Supplementary Fig.16i for Cluster1, Supplementary Fig.16j for Cluster2) for the RAC sites located in the last 400 nt region of human internal exons (length: > 200 nt & < 400 nt). Red dots (Increased: n = 9249 for 1800 exons of Cluster1, n = 2905 for 1800 exons of Cluster2), blue dots (Decreased: n = 6 for 1800 exons of Cluster1, n = 130 for 1800 exons of Cluster2), and grey dots (no change: n = 11336 for 1800 exons of Cluster1, n = 13096 for 1800 exons of Cluster2) were those sites that had increased probability (> 0.1), decreased probability (< -0.1), or not change probability (|ΔProbability| <= 0.1) respectively by introns deletion.

k-l, Positional plot of ΔProbability (Supplementary Fig.16k for Cluster1, Supplementary Fig.16l for Cluster2) for the RAC sites located in the last 500 nt region of human internal exons (length: >= 400 nt). Red dots (Increased: n = 1494 for 300 exons of Cluster1, n = 377 for 300 exons of Cluster2), blue dots (Decreased: n = 196 for 300 exons of Cluster1, n = 377 for 300 exons of Cluster2), and grey dots (no change: n = 4308 for 300 exons of Cluster1, n = 4144 for 300 exons of Cluster2) were those sites that had increased probability (> 0.1), decreased probability (< -0.1), or not change probability (|ΔProbability| <= 0.1) respectively by introns deletion.

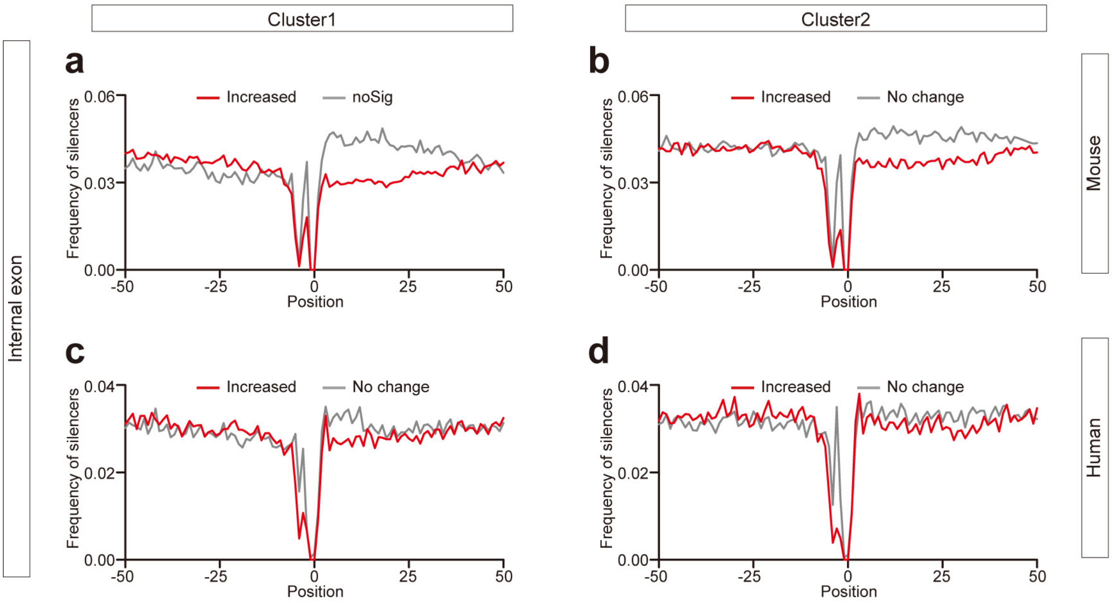
Cluster1
Cluster2
Internal exon
Mouse
Human
a
b
c
d
Increased
noSig
No change
Frequency of silencers
Position
0.06
0.03
0.00
0.04
0.02
-50
-25
0
25
50

Supplementary Fig.17: m^{6}A silencers avoids 50 nt downstream of latent sites.

a,b,c,d, Positional plot for the frequency of top 50 m^{6}A silencers (Supplementary Fig.17a,c for Cluster1 in mouse or human, Supplementary Fig.17b,d for Cluster2 in mouse or human) in mRNA sequences around the RAC sites. These sites were in internal exons, and the plots were compared between the increased sites (red line, ΔProbability > 0.1) and no change sites (grey line, |ΔProbability| <= 0.1).

Supplementary Fig18

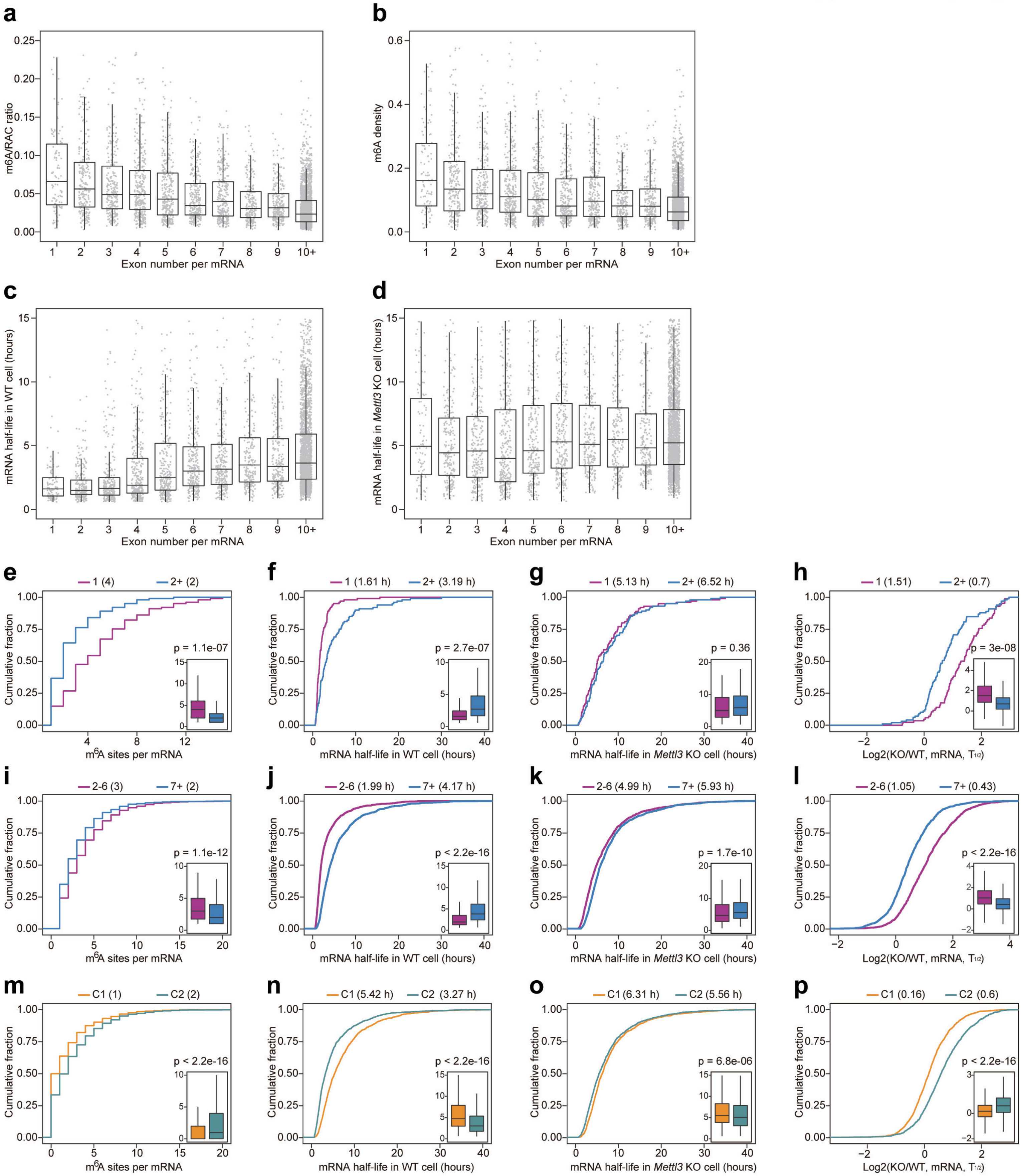

Supplementary Fig.18: The m^{6}A deposition inhibition by exon-intron boundary allows longer mRNA half-lives.

a,b,c,d, Boxplots demonstrating the correlation between exon number per mRNA and m^{6}A/RAC ratio (Supplementary Fig.18a), m^{6}A density per 100 nt (Supplementary Fig.18b), mRNA $T_{1/2}$s in *Mettl3* WT mouse ES cells (Supplementary Fig.18c), mRNA $T_{1/2}$s in *Mettl3* knockout mouse ES cells (Supplementary Fig.18d). Median and interquartile ranges were presented for the box plot, and each dot represented a unique mRNA.

e,f,g,h, Cumulative distribution and boxplots (inset) showing m^{6}A sites number (Supplementary Fig.18e), mRNA $T_{1/2}$s in *Mettl3* WT mouse ES cells (Supplementary Fig.18f), mRNA $T_{1/2}$s in *Mettl3* knockout cells (Supplementary Fig.18g), and mRNA $T_{1/2}$s changes upon global m^{6}A loss (Supplementary Fig.18h) in single (n = 101) and multiple exon genes (n = 101). The box represents the 1st to 3rd quartile with the median marked by a horizontal line. The P-values were calculated by two-sided Wilcoxon test.

i,j,k,l, Cumulative distribution and boxplots (inset) showing m^{6}A sites number (Supplementary Fig.18i), mRNA $T_{1/2}$s in *Mettl3* WT mouse ES cells (Supplementary Fig.18j), mRNA $T_{1/2}$s in *Mettl3* knockout cells (Supplementary Fig.18k), and mRNA $T_{1/2}$s changes upon global m^{6}A loss (Supplementary Fig.18l) in genes with 2-6 exons (n = 1210) and genes with > 6 exons (n = 1210). The box represents the 1st to 3rd quartile with the median marked by a horizontal line. The P-values were calculated by two-sided Wilcoxon test.

m,n,o,p, Cumulative distribution and boxplots (inset) showing m^{6}A sites number (Supplementary Fig.18m), mRNA $T_{1/2}$s in *Mettl3* WT mouse ES cells (Supplementary Fig.18n), mRNA $T_{1/2}$s in *Mettl3* knockout cells (Supplementary Fig.18o), and mRNA $T_{1/2}$s changes upon global m^{6}A loss (Supplementary Fig.18p) in genes of Cluster1 (n = 1330) and genes of Cluster2 (n = 1330). The box represents the 1st to 3rd quartile with the median marked by a horizontal line. The P-values were calculated by two-sided Wilcoxon test.

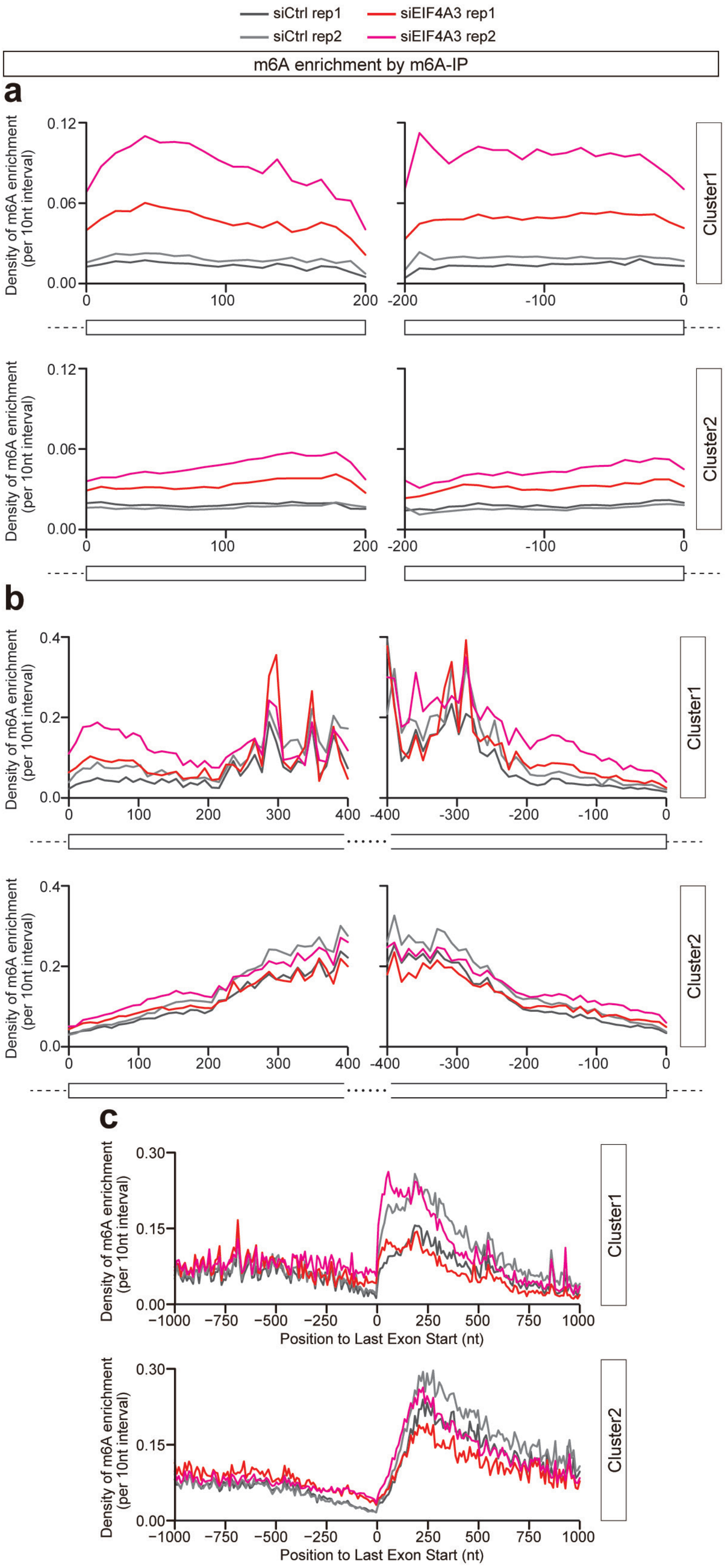
siCtrl rep1
siCtrl rep2
siEIF4A3 rep1
siEIF4A3 rep2
m6A enrichment by m6A-IP
a
Density of m6A enrichment (per 10nt interval)
0.12
0.06
0.00
0
100
200
-200
-100
0
Cluster1
Cluster2
b
0.4
0.2
0.0
400
-400
-300
-200
-100
300
c
0.30
0.15
0.00
−1000
−750
−500
−250
250
500
750
1000
Position to Last Exon Start (nt)

Supplementary Fig.19: Exon junction complex partially contributes to m^6A deposition inhibition by exon-intron boundary.

a, The m^6A enrichment density at short internal exons (<= 200 nt) were compared between siCtrl (grey lines) and siEIF4A3 (red lines). These internal exons were divided into two clusters (Cluster1 vs. Cluster2) based on clustering result of Supplementary Fig. 11.

b, The m^6A enrichment density at long internal exons (> 200 nt) were compared between siCtrl (grey lines) and siEIF4A3 (red lines). These internal exons were divided into two clusters (Cluster1 vs. Cluster2) based on clustering result of Supplementary Fig. 11.

c, The m^6A enrichment density around last exon start were compared between siCtrl (grey lines) and siEIF4A3 (red lines). These last exons were divided into two clusters (Cluster1 vs. Cluster2) based on clustering result of Supplementary Fig. 8.

Uncropped gel for Fig. 3b

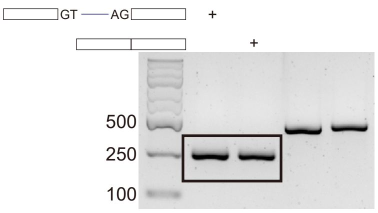

Lrp12

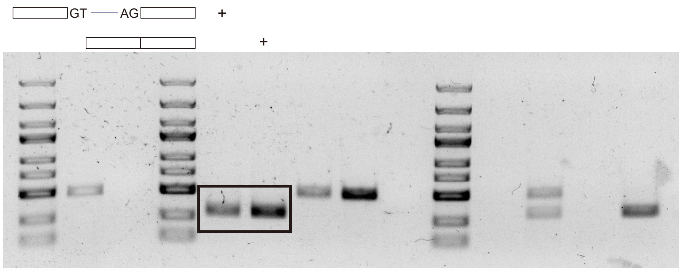

Gne

Supplementary Fig.20: Uncropped gel for Fig.3b

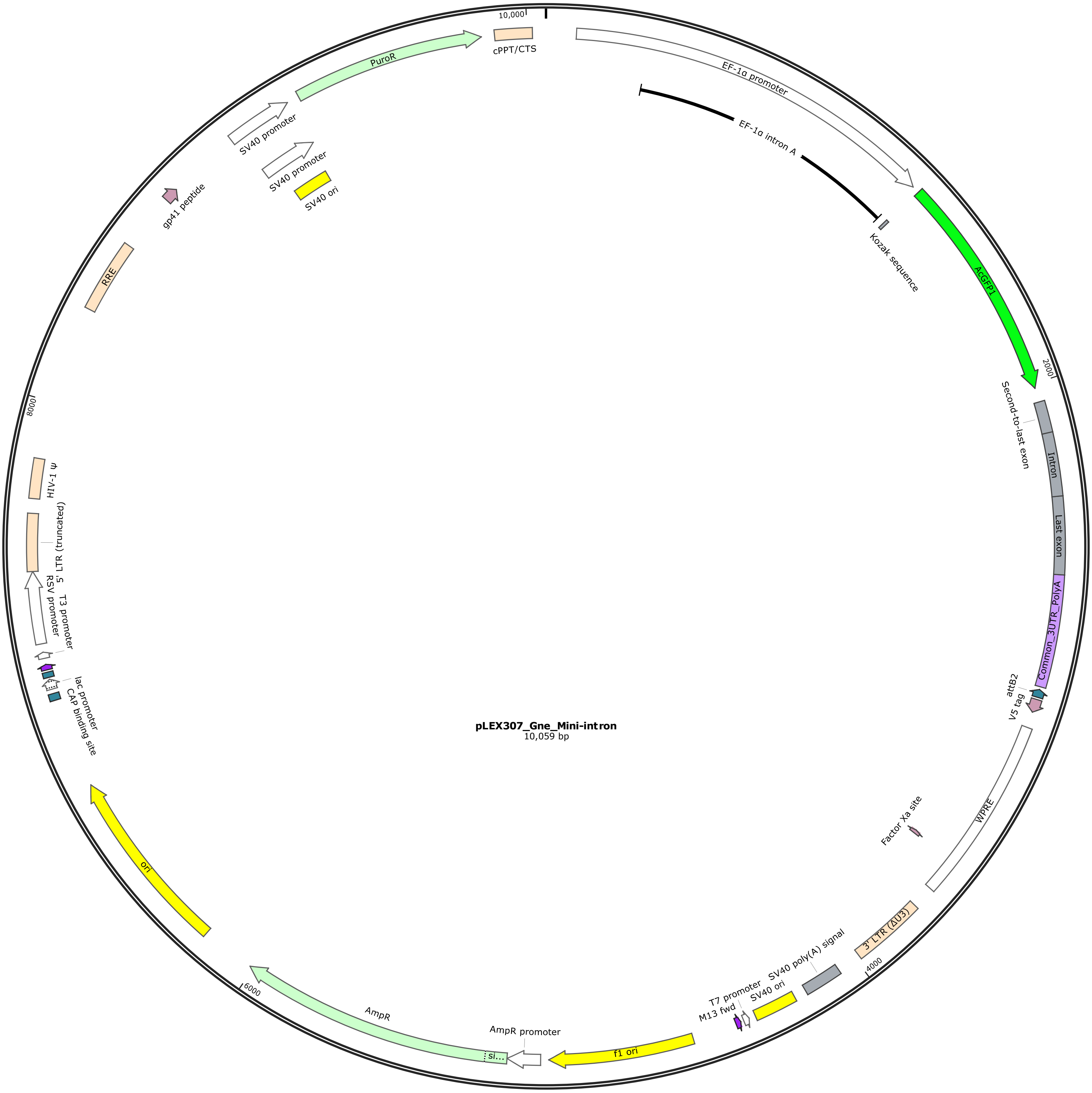
pLEX307_Gne_Mini-intron
10,059 bp
10,000
2000
4000
6000
8000
cPPT/CTS
EF-1α promoter
EF-1α intron A
Kozak sequence
AcGFP1
Second-to-last exon
Intron
Last exon
Common_3UTR_PolyA
attB2
V5 tag
WPRE
Factor Xa site
3' LTR (ΔU3)
SV40 poly(A) signal
SV40 ori
T7 promoter
M13 fwd
f1 ori
AmpR promoter
AmpR
ori
lac promoter
CAP binding site
T3 promoter
RSV promoter
5' LTR (truncated)
HIV-1 Ψ
RRE
gp41 peptide
SV40 ori
SV40 promoter
SV40 promoter
PuroR

CTCGAGGCCTGCAGGTGCAAAGATGGATAAAGTTTTAAACAGAGAGGAATCTTTGCAGCTAATGGACCTTC
TAGGTCTTGAAAGGAGTGGGAATTGGCTCCGGTGCCCGTCAGTGGGCAGAGCGCACATCGCCCACAGTCC
CCGAGAAGTTGGGGGGAGGGGTCGGCAATTGAACCGGTGCCTAGAGAAGGTGGCGCGGGGTAAACTGG
GAAAGTGATGTCGTGTACTGGCTCCGCCTTTTTCCCGAGGGTGGGGGAGAACCGTATATAAGTGCAGTAGT
CGCCGTGAACGTTCTTTTTCGCAACGGGTTTGCCGCCAGAACACAGGTAAGTGCCGTGTGTGGTTCCCGCG
GGCCTGGCCTCTTTACGGGTTATGGCCCTTGCGTGCCTTGAATTACTTCCACCTGGCTGCAGTACGTGATTCT
TGATCCCGAGCTTCGGGTTGGAAGTGGGTGGGAGAGTTCGAGGCCTTGCGCTTAAGGAGCCCCTTCGCCT
CGTGCTTGAGTTGAGGCCTGGCCTGGGCGCTGGGGCCGCCGCGTGCGAATCTGGTGGCACCTTCGCGCCT
GTCTCGCTGCTTTCGATAAGTCTCTAGCCATTTAAAATTTTTGATGACCTGCTGCGACGCTTTTTTTCTGGCAA
GATAGTCTTGTAAATGCGGGCCAAGATCTGCACACTGGTATTTCGGTTTTTGGGGCCGCGGGCGGCGACGG
GGCCCGTGCGTCCCAGCGCACATGTTCGGCGAGGCGGGGCCTGCGAGCGCGGCCACCGAGAATCGGACG
GGGGTAGTCTCAAGCTGGCCGGCCTGCTCTGGTGCCTGGCCTCGCGCCGCCGTGTATCGCCCCGCCCTGGG
CGGCAAGGCTGGCCCGGTCGGCACCAGTTGCGTGAGCGGAAAGATGGCCGCTTCCCGGCCCTGCTGCAG
GGAGCTCAAAATGGAGGACGCGGCGCTCGGGAGAGCGGGCGGGTGAGTCACCCACACAAAGGAAAAGG
GCCTTTCCGTCCTCAGCCGTCGCTTCATGTGACTCCACGGAGTACCGGGCGCCGTCCAGGCACCTCGATTAG
TTCTCGAGCTTTTGGAGTACGTCGTCTTTAGGTTGGGGGGAGGGGTTTTATGCGATGGAGTTTCCCCACACT
GAGTGGGTGGAGACTGAAGTTAGGCCAGCTTGGCACTTGATGTAATTCTCCTTGGAATTTGCCCTTTTTGAG
TTTGGATCTTGGTTCATTCTCAAGCCTCAGACAGTGGTTCAAAGTTTTTTTCTTCCATTTCAGGTGTCGTGAG
GCTAGCgctaccggtcgccaccatggtgagcaagggcgccgagctgttcaccggcatcgtgcccatcctgatcgagctgaatggcgatgt
gaatggccacaagttcagcgtgagcggcgagggcgagggcgatgccacctacggcaagcttaccctgaagttcatctgcaccaccggcaa
gctgcctgtgccctggcccaccctggttaccaccctgagctacggcgtgcagtgcttctcacgctaccccgatcacatgaagcagcacgatttc
ttcaagagcgccatgcctgagggctacatccaggagcgcaccatcttcttcgaggatgatggcaattacaagtcgcgcgccgaggtgaagtt
cgagggcgataccctggtgaatcgcatcgagcttaccggcaccgatttcaaggaggatggcaatatcctgggcaataagatggagtacaatt
acaatgcccacaatgtgtacatcatgacggataaggccaagaatggcatcaaggtgaatttcaagatccgccacaatatcgaggatggcag
cgtgcagctggccgatcactaccagcagaatacccccatcggcgatggccctgtgctgctgcccgataatcactacctgtccacccagagcg
ccctgtccaaggatcccaatgagaagcgcgatcacatgatctacttcggcttcgttaccgccgccgccatcacccacggcatggatgagctgt
acaagtccgggctcagatctcgagctcaagcttcgaattctgcagtcgatGAAGGGATGTCAGTACCAAAAGACGAAGCTGT
GGGTGCCCTCCATCTCATCCAGGCTGCCAAGCTGGGCAACGTGAAGGCCCAGAGCATCTTACGAACAGGTG
AGCCTGCGGTCCCAGATGCCCAGGGCTGCTGGAGAGCCTCAGTTTCCCTCAGGCGCAGACACTTTTCCTGC
CGCCGAGTGGGGAAGTGTTGATGCTTctatctcacctggccttcacaaacagcttttaAATGTAGCACTTCCTGGAGTT
TGATGTAAAGTTTTTTTTAAATGTCTCTGCTTTTACCCTCCTGCAGCTGGAACTGCTTTGGGACTTGGGGTTG
TGAACATCCTCCACACTATGAATCCTTCCCTGGTGATCCTGTCTGGAGTCCTGGCCAGTCACTACATCCACATC
GTGAAGGACGTCATCCGCCAGCAAGCCTTGTCCTCCGTGCAGGATGTGGACGTGGTGGTCTCAGACTTGGT
GGACCCGGCCCTGCTTGGCGCAGCCAGCATGGTTCTGGACTACACAACGCGCAGGATCCACTAGGTCTCCC
Ggtaccgcgggcccgggatccaccggatctagatatctgatcataatcagccataccacatttgtagaggttttacttgctttaaaaatcctcc
cacacctccccctgatcctgaatcataaaatgaatgcaattgttgttgttatcttgtttattgcagcttataatggttacaaataaagcaatagc
atcacaaatttcacaaataaagcatttttttcactgcattctagttgtggtttgtccaatctcatcaatgtatcttatcgcgtaaattgtaagcgtt
aatattttgttaaaattcgcgttaaatttttgttaaatcagctcattttttatccaataggccgaaatcgggcgcgccgacccagctttCTTGTA
CAAAGTGGTTGATATCGGTAAGCCTATCCCTAACCCTCTCCTCGGTCTCGATTCTACGTAGTAATGAACTAGTA
CCGGTTAAGTCGACAATCAACGCGTTAAGTCGACAATCAACCTCTGGATTACAAAATTTGTGAAAGATTGAC
TGGTATTCTTAACTATGTTGCTCCTTTTACGCTATGTGGATACGCTGCTTTAATGCCTTTGTATCATGCTATTGCT
TCCCGTATGGCTTTCATTTTCTCCTCCTTGTATAAATCCTGGTTGCTGTCTCTTTATGAGGAGTTGTGGCCCGTT
GTCAGGCAACGTGGCGTGGTGTGCACTGTGTTTGCTGACGCAACCCCCACTGGTTGGGGCATTGCCACCAC
CTGTCAGCTCCTTTCCGGGACTTTCGCTTTCCCCCTCCCTATTGCCACGGCGGAACTCATCGCCGCCTGCCTT

GCCCGCTGCTGGACAGGGGCTCGGCTGTTGGGCACTGACAATTCCGTGGTGTTGTCGGGGAAATCATCGTC
CTTTCCTTGGCTGCTCGCCTGTGTTGCCACCTGGATTCTGCGCGGGACGTCCTTCTGCTACGTCCCTTCGGCC
CTCAATCCAGCGGACCTTCCTTCCCGCGGCCTGCTGCCGGCTCTGCGGCCTCTTCCGCGTCTTCGCCTTCGC
CCTCAGACGAGTCGGATCTCCCTTTGGGCCGCCTCCCCGCGTCGACTTTAAGACCAATGACTTACAAGGCAG
CTGTAGATCTTAGCCACTTTTTAAAAGAAAAGGGGGGACTGGAAGGGCTAATTCACTCCCAACGAAGACAA
GATCTGCTTTTTGCTTGTACTGGGTCTCTCTGGTTAGACCAGATCTGAGCCTGGGAGCTCTCTGGCTAACTAG
GGAACCCACTGCTTAAGCCTCAATAAAGCTTGCCTTGAGTGCTTCAAGTAGTGTGTGCCCGTCTGTTGTGTG
ACTCTGGTAACTAGAGATCCCTCAGACCCTTTTAGTCAGTGTGGAAAATCTCTAGCAGTACGTATAGTAGTTC
ATGTCATCTTATTATTCAGTATTTATAACTTGCAAAGAAATGAATATCAGAGAGTGAGAGGAACTTGTTTATTG
CAGCTTATAATGGTTACAAATAAAGCAATAGCATCACAAATTTCACAAATAAAGCATTTTTTTCACTGCATTCT
AGTTGTGGTTTGTCCAAACTCATCAATGTATCTTATCATGTCTGGCTCTAGCTATCCCGCCCCTAACTCCGCCCA
TCCCGCCCCTAACTCCGCCCAGTTCCGCCCATTCTCCGCCCCATGGCTGACTAATTTTTTTTATTTATGCAGAG
GCCGAGGCCGCCTCGGCCTCTGAGCTATTCCAGAAGTAGTGAGGAGGCTTTTTTGGAGGCCTAGGGACGT
ACCCAATTCGCCCTATAGTGAGTCGTATTACGCGCGCTCACTGGCCGTCGTTTTACAACGTCGTGACTGGGA
AAACCCTGGCGTTACCCAACTTAATCGCCTTGCAGCACATCCCCCTTTCGCCAGCTGGCGTAATAGCGAAGA
GGCCCGCACCGATCGCCCTTCCCAACAGTTGCGCAGCCTGAATGGCGAATGGGACGCGCCCTGTAGCGGC
GCATTAAGCGCGGCGGGTGTGGTGGTTACGCGCAGCGTGACCGCTACACTTGCCAGCGCCCTAGCGCCCGC
TCCTTTCGCTTTCTTCCCTTCCTTTCTCGCCACGTTCGCCGGCTTTCCCCGTCAAGCTCTAAATCGGGGGCTCC
CTTTAGGGTTCCGATTTAGTGCTTTACGGCACCTCGACCCCAAAAAACTTGATTAGGGTGATGGTTCACGTA
GTGGGCCATCGCCCTGATAGACGGTTTTTCGCCCTTTGACGTTGGAGTCCACGTTCTTTAATAGTGGACTCTT
GTTCCAAACTGGAACAACACTCAACCCTATCTCGGTCTATTCTTTTGATTTATAAGGGATTTTGCCGATTTCGG
CCTATTGGTTAAAAAATGAGCTGATTTAACAAAAATTTAACGCGAATTTTAACAAAATATTAACGCTTACAATT
TAGGTGGCACTTTTCGGGGAAATGTGCGCGGAACCCCTATTTGTTTATTTTTCTAAATACATTCAAATATGTAT
CCGCTCATGAGACAATAACCCTGATAAATGCTTCAATAATATTGAAAAAGGAAGAGTATGAGTATTCAACATT
TCCGTGTCGCCCTTATTCCCTTTTTTGCGGCATTTTGCCTTCCTGTTTTTGCTCACCCAGAAACGCTGGTGAA
AGTAAAAGATGCTGAAGATCAGTTGGGTGCACGAGTGGGTTACATCGAACTGGATCTCAACAGCGGTAAGA
TCCTTGAGAGTTTTCGCCCCGAAGAACGTTTTCCAATGATGAGCACTTTTAAAGTTCTGCTATGTGGCGCGG
TATTATCCCGTATTGACGCCGGGCAAGAGCAACTCGGTCGCCGCATACACTATTCTCAGAATGACTTGGTTGA
GTACTCACCAGTCACAGAAAAGCATCTTACGGATGGCATGACAGTAAGAGAATTATGCAGTGCTGCCATAAC
CATGAGTGATAACACTGCGGCCAACTTACTTCTGACAACGATCGGAGGACCGAAGGAGCTAACCGCTTTTTT
GCACAACATGGGGGATCATGTAACTCGCCTTGATCGTTGGGAACCGGAGCTGAATGAAGCCATACCAAACG
ACGAGCGTGACACCACGATGCCTGTAGCAATGGCAACAACGTTGCGCAAACTATTAACTGGCGAACTACTTA
CTCTAGCTTCCCGGCAACAATTAATAGACTGGATGGAGGCGGATAAAGTTGCAGGACCACTTCTGCGCTCG
GCCCTTCCGGCTGGCTGGTTTATTGCTGATAAATCTGGAGCCGGTGAGCGTGGGTCTCGCGGTATCATTGCA
GCACTGGGGCCAGATGGTAAGCCCTCCCGTATCGTAGTTATCTACACGACGGGGAGTCAGGCAACTATGGAT
GAACGAAATAGACAGATCGCTGAGATAGGTGCCTCACTGATTAAGCATTGGTAACTGTCAGACCAAGTTTAC
TCATATATACTTTAGATTGATTTAAAACTTCATTTTTAATTTAAAAGGATCTAGGTGAAGATCCTTTTTGATAATC
TCATGACCAAAATCCCTTAACGTGAGTTTTCGTTCCACTGAGCGTCAGACCCCGTAGAAAAGATCAAAGGAT
CTTCTTGAGATCCTTTTTTTCTGCGCGTAATCTGCTGCTTGCAAACAAAAAAACCACCGCTACCAGCGGTGG
TTTGTTTGCCGGATCAAGAGCTACCAACTCTTTTTCCGAAGGTAACTGGCTTCAGCAGAGCGCAGATACCAA
ATACTGTTCTTCTAGTGTAGCCGTAGTTAGGCCACCACTTCAAGAACTCTGTAGCACCGCCTACATACCTCGCT
CTGCTAATCCTGTTACCAGTGGCTGCTGCCAGTGGCGATAAGTCGTGTCTTACCGGGTTGGACTCAAGACGA
TAGTTACCGGATAAGGCGCAGCGGTCGGGCTGAACGGGGGGTTCGTGCACACAGCCCAGCTTGGAGCGA
ACGACCTACACCGAACTGAGATACCTACAGCGTGAGCTATGAGAAAGCGCCACGCTTCCCGAAGGGAGAA

AGGCGGACAGGTATCCGGTAAGCGGCAGGGTCGGAACAGGAGAGCGCACGAGGGAGCTTCCAGGGGGA
AACGCCTGGTATCTTTATAGTCCTGTCGGGTTTCGCCACCTCTGACTTGAGCGTCGATTTTTGTGATGCTCGTC
AGGGGGGCGGAGCCTATGGAAAAACGCCAGCAACGCGGCCTTTTTACGGTTCCTGGCCTTTTGCTGGCCT
TTTGCTCACATGTTCTTTCCTGCGTTATCCCCTGATTCTGTGGATAACCGTATTACCGCCTTTGAGTGAGCTGA
TACCGCTCGCCGCAGCCGAACGACCGAGCGCAGCGAGTCAGTGAGCGAGGAAGCGGAAGAGCGCCCAAT
ACGCAAACCGCCTCTCCCCGCGCGTTGGCCGATTCATTAATGCAGCTGGCACGACAGGTTTCCCGACTGGA
AAGCGGGCAGTGAGCGCAACGCAATTAATGTGAGTTAGCTCACTCATTAGGCACCCCAGGCTTTACACTTTA
TGCTTCCGGCTCGTATGTTGTGTGGAATTGTGAGCGGATAACAATTTCACACAGGAAACAGCTATGACCATG
ATTACGCCAAGCGCGCAATTAACCCTCACTAAAGGGAACAAAAGCTGGAGCTGCAAGCTTAATGTAGTCTTA
TGCAATACTCTTGTAGTCTTGCAACATGGTAACGATGAGTTAGCAACATGCCTTACAAGGAGAGAAAAAGCA
CCGTGCATGCCGATTGGTGGAAGTAAGGTGGTACGATCGTGCCTTATTAGGAAGGCAACAGACGGGTCTGA
CATGGATTGGACGAACCACTGAATTGCCGCATTGCAGAGATATTGTATTTAAGTGCCTAGCTCGATACATAAA
CGGGTCTCTCTGGTTAGACCAGATCTGAGCCTGGGAGCTCTCTGGCTAACTAGGGAACCCACTGCTTAAGCC
TCAATAAAGCTTGCCTTGAGTGCTTCAAGTAGTGTGTGCCCGTCTGTTGTGTGACTCTGGTAACTAGAGATCC
CTCAGACCCTTTTAGTCAGTGTGGAAAATCTCTAGCAGTGGCGCCCGAACAGGGACTTGAAAGCGAAAGG
GAAACCAGAGGAGCTCTCTCGACGCAGGACTCGGCTTGCTGAAGCGCGCACGGCAAGAGGCGAGGGGC
GGCGACTGGTGAGTACGCCAAAAATTTTGACTAGCGGAGGCTAGAAGGAGAGAGATGGGTGCGAGAGCG
TCAGTATTAAGCGGGGGAGAATTAGATCGCGATGGGAAAAAATTCGGTTAAGGCCAGGGGGAAAGAAAAA
ATATAAATTAAAACATATAGTATGGGCAAGCAGGGAGCTAGAACGATTCGCAGTTAATCCTGGCCTGTTAGAA
ACATCAGAAGGCTGTAGACAAATACTGGGACAGCTACAACCATCCCTTCAGACAGGATCAGAAGAACTTAG
ATCATTATATAATACAGTAGCAACCCTCTATTGTGTGCATCAAAGGATAGAGATAAAAGACACCAAGGAAGCT
TTAGACAAGATAGAGGAAGAGCAAAACAAAAGTAAGACCACCGCACAGCAAGCGGCCGCTGATCTTCAGA
CCTGGAGGAGGAGATATGAGGGACAATTGGAGAAGTGAATTATATAAATATAAAGTAGTAAAAATTGAACCA
TTAGGAGTAGCACCCACCAAGGCAAAGAGAAGAGTGGTGCAGAGAGAAAAAAGAGCAGTGGGAATAGG
AGCTTTGTTCCTTGGGTTCTTGGGAGCAGCAGGAAGCACTATGGGCGCAGCGTCAATGACGCTGACGGTAC
AGGCCAGACAATTATTGTCTGGTATAGTGCAGCAGCAGAACAATTTGCTGAGGGCTATTGAGGCGCAACAG
CATCTGTTGCAACTCACAGTCTGGGGCATCAAGCAGCTCCAGGCAAGAATCCTGGCTGTGGAAAGATACCT
AAAGGATCAACAGCTCCTGGGGATTTGGGGTTGCTCTGGAAAACTCATTTGCACCACTGCTGTGCCTTGGA
ATGCTAGTTGGAGTAATAAATCTCTGGAACAGATTTGGAATCACACGACCTGGATGGAGTGGGACAGAGAA
ATTAACAATTACACAAGCTTAATACACTCCTTAATTGAAGAATCGCAAAACCAGCAAGAAAAGAATGAACAA
GAATTATTGGAATTAGATAAATGGGCAAGTTTGTGGAATTGGTTTAACATAACAAATTGGCTGTGGTATATAA
AATTATTCATAATGATAGTAGGAGGCTTGGTAGGTTTAAGAATAGTTTTTGCTGTACTTTCTATAGTGAATAGA
GTTAGGCAGGGATATTCACCATTATCGTTTCAGACCCACCTCCCAACCCCGAGGGGACCCATGCATTGCATCT
CAATTAGTCAGCAACCAGGTGTGGAAAGTCCCCAGGCTCCCCAGCAGGCAGAAGTATGCAAAGCATGCGTC
TCAATTAGTCAGCAACCATAGTCCCGCCCCTAACTCCGCCCATCCCGCCCCTAACTCCGCCCAGTTCCGCCCAT
TCTCCGCCCCATGGCTGACTAATTTTTTTTATTTATGCAGAGGCCGAGGCCGCCTCGGCCTCTGAGCTATTCC
AGAAGTAGTGAGGAGGCTTTTTTGGAGGCCTAGGCTTTTGCAAAAAGCTTTCTAGAGGTACCACCGGAGC
TTACCATGACCGAGTACAAGCCCACGGTGCGCCTCGCCACCCGCGACGACGTCCCCAGGGCCGTACGCACC
CTCGCCGCCGCGTTCGCCGACTACCCCGCCACGCGCCACACCGTCGATCCGGACCGCCACATCGAGCGGGT
CACCGAGCTGCAAGAACTCTTCCTCACGCGCGTCGGGCTCGACATCGGCAAGGTGTGGGTCGCGGACGAC
GGCGCCGCCGTGGCGGTCTGGACCACGCCGGAGAGCGTCGAAGCGGGGGCGGTGTTCGCCGAGATCGGC
CCGCGCATGGCCGAGTTGAGCGGTTCCCGGCTGGCCGCGCAGCAACAGATGGAAGGCCTCCTGGCGCCGC
ACCGGCCCAAGGAGCCCGCGTGGTTCCTGGCCACCGTCGGCGTCTCGCCCGACCACCAGGGCAAGGGTCT
GGGCAGCGCCGTCGTGCTCCCCGGAGTGGAGGCGGCCGAGCGCGCCGGGGTGCCCGCCTTCCTGGAGAC

CTCCGCGCCCCGCAACCTCCCCTTCTACGAGCGGCTCGGCTTCACCGTCACCGCCGACGTCGAGGTGCCCG
AAGGACCGCGCACCTGGTGCATGACCCGCAAGCCCGGTGCCAGTTAACGAATTCTAGATCTTGAGACAAAT
GGCAGTATTCATCCACAATTTTAAAAGAAAAGGGGGGATTGGGGGGTACAGTGCAGGGGAAAGAATAGTA
GACATAATAGCAACAGACATACAAACTAAAGAATTACAAAAACAAATTACAAAAATTCAAAATTTTCGGGTTT
ATTACAGGGACAGCAGAGATCCACTTTGGCGCCGG

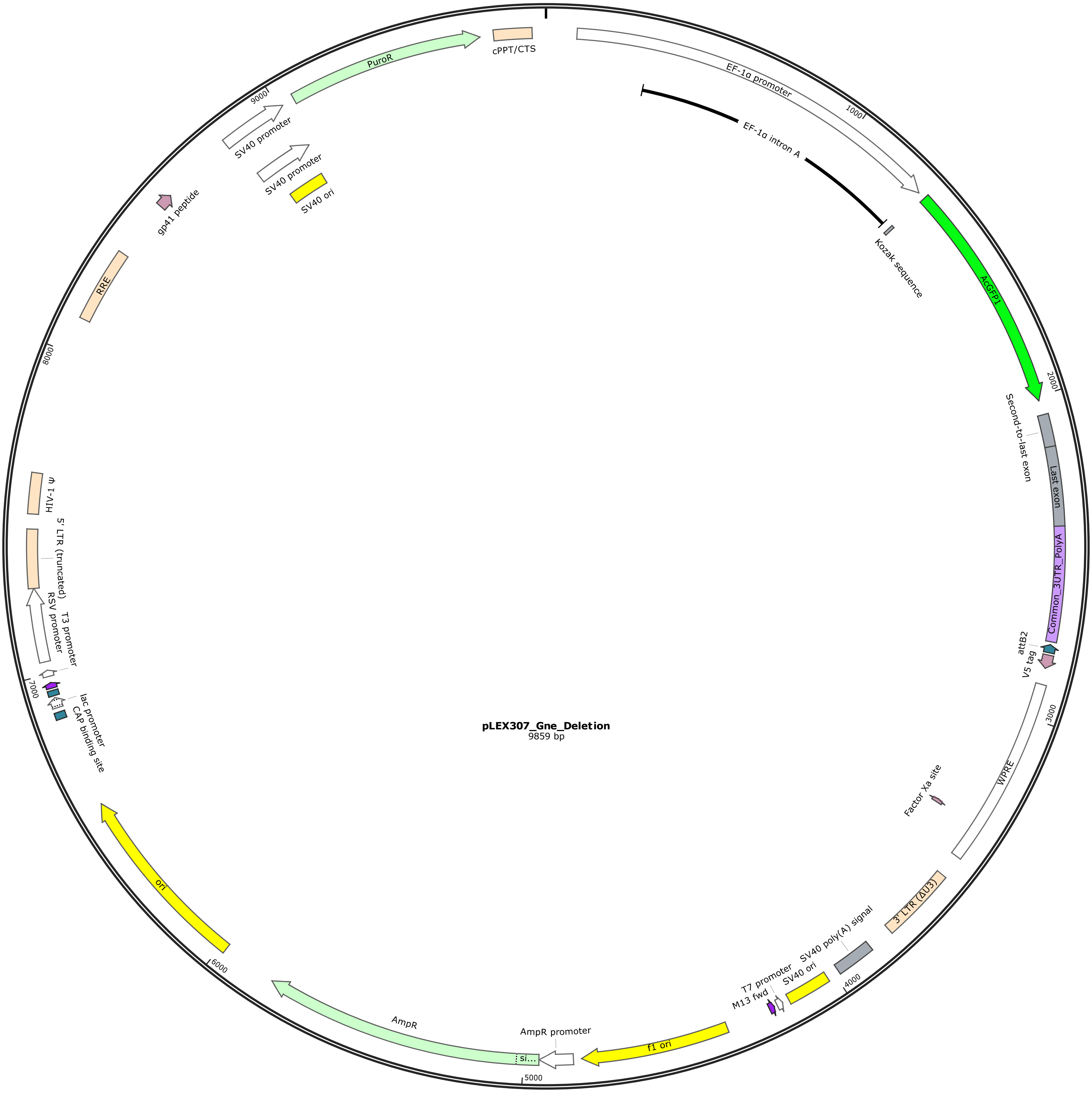
pLEX307_Gne_Deletion
9859 bp
EF-1α promoter
EF-1α intron A
Kozak sequence
AcGFP1
Second-to-last exon
Last exon
Common_3UTR_PolyA
attB2
V5 tag
WPRE
Factor Xa site
3' LTR (ΔU3)
SV40 poly(A) signal
SV40 ori
T7 promoter
M13 fwd
f1 ori
AmpR promoter
AmpR
si...
ori
lac promoter
CAP binding site
T3 promoter
RSV promoter
5' LTR (truncated)
HIV-1 Ψ
RRE
gp41 peptide
SV40 promoter
SV40 promoter
SV40 ori
PuroR
cPPT/CTS
1000
2000
3000
4000
5000
6000
7000
8000
9000

CTCGAGGCCTGCAGGTGCAAAGATGGATAAAGTTTTAAACAGAGAGGAATCTTTGCAGCTAATGGACCTTC
TAGGTCTTGAAAGGAGTGGGAATTGGCTCCGGTGCCCGTCAGTGGGCAGAGCGCACATCGCCCACAGTCC
CCGAGAAGTTGGGGGGAGGGGTCGGCAATTGAACCGGTGCCTAGAGAAGGTGGCGCGGGGTAAACTGG
GAAAGTGATGTCGTGTACTGGCTCCGCCTTTTTCCCGAGGGTGGGGGAGAACCGTATATAAGTGCAGTAGT
CGCCGTGAACGTTCTTTTTCGCAACGGGTTTGCCGCCAGAACACAGGTAAGTGCCGTGTGTGGTTCCCGCG
GGCCTGGCCTCTTTACGGGTTATGGCCCTTGCGTGCCTTGAATTACTTCCACCTGGCTGCAGTACGTGATTCT
TGATCCCGAGCTTCGGGTTGGAAGTGGGTGGGAGAGTTCGAGGCCTTGCGCTTAAGGAGCCCCTTCGCCT
CGTGCTTGAGTTGAGGCCTGGCCTGGGCGCTGGGGCCGCCGCGTGCGAATCTGGTGGCACCTTCGCGCCT
GTCTCGCTGCTTTCGATAAGTCTCTAGCCATTTAAAATTTTTGATGACCTGCTGCGACGCTTTTTTTCTGGCAA
GATAGTCTTGTAAATGCGGGCCAAGATCTGCACACTGGTATTTCGGTTTTTGGGGCCGCGGGCGGCGACGG
GGCCCGTGCGTCCCAGCGCACATGTTCGGCGAGGCGGGGCCTGCGAGCGCGGCCACCGAGAATCGGACG
GGGGTAGTCTCAAGCTGGCCGGCCTGCTCTGGTGCCTGGCCTCGCGCCGCCGTGTATCGCCCCGCCCTGGG
CGGCAAGGCTGGCCCGGTCGGCACCAGTTGCGTGAGCGGAAAGATGGCCGCTTCCCGGCCCTGCTGCAG
GGAGCTCAAAATGGAGGACGCGGCGCTCGGGAGAGCGGGCGGGTGAGTCACCCACACAAAGGAAAAGG
GCCTTTCCGTCCTCAGCCGTCGCTTCATGTGACTCCACGGAGTACCGGGCGCCGTCCAGGCACCTCGATTAG
TTCTCGAGCTTTTGGAGTACGTCGTCTTTAGGTTGGGGGGAGGGGTTTTATGCGATGGAGTTTCCCCACACT
GAGTGGGTGGAGACTGAAGTTAGGCCAGCTTGGCACTTGATGTAATTCTCCTTGGAATTTGCCCTTTTTGAG
TTTGGATCTTGGTTCATTCTCAAGCCTCAGACAGTGGTTCAAAGTTTTTTTCTTCCATTTCAGGTGTCGTGAG
GCTAGCgctaccggtcgccaccatggtgagcaagggcgccgagctgttcaccggcatcgtgcccatcctgatcgagctgaatggcgatgt
gaatggccacaagttcagcgtgagcggcgagggcgagggcgatgccacctacggcaagcttaccctgaagttcatctgcaccaccggcaa
gctgcctgtgccctggcccaccctggttaccaccctgagctacggcgtgcagtgcttctcacgctaccccgatcacatgaagcagcacgatttc
ttcaagagcgccatgcctgagggctacatccaggagcgcaccatcttcttcgaggatgatggcaattacaagtcgcgcgccgaggtgaagtt
cgagggcgataccctggtgaatcgcatcgagcttaccggcaccgatttcaaggaggatggcaatatcctgggcaataagatggagtacaatt
acaatgcccacaatgtgtacatcatgacggataaggccaagaatggcatcaaggtgaatttcaagatccgccacaatatcgaggatggcag
cgtgcagctggccgatcactaccagcagaatacccccatcggcgatggccctgtgctgctgcccgataatcactacctgtccacccagagcg
ccctgtccaaggatcccaatgagaagcgcgatcacatgatctacttcggcttcgttaccgccgccgccatcacccacggcatggatgagctgt
acaagtccgggctcagatctcgagctcaagcttcgaattctgcagtcgatGAAGGGATGTCAGTACCAAAAGACGAAGCTGT
GGGTGCCCTCCATCTCATCCAGGCTGCCAAGCTGGGCAACGTGAAGGCCCAGAGCATCTTACGAACAGCTG
GAACTGCTTTGGGACTTGGGGTTGTGAACATCCTCCACACTATGAATCCTTCCCTGGTGATCCTGTCTGGAG
TCCTGGCCAGTCACTACATCCACATCGTGAAGGACGTCATCCGCCAGCAAGCCTTGTCCTCCGTGCAGGATG
TGGACGTGGTGGTCTCAGACTTGGTGGACCCGGCCCTGCTTGGCGCAGCCAGCATGGTTCTGGACTACACA
ACGCGCAGGATCCACTAGGTCTCCCGgtaccgcgggcccgggatccaccggatctagatatctgatcataatcagccataccaca
tttgtagaggttttacttgctttaaaaatcctcccacacctccccctgatcctgaatcataaaatgaatgcaattgttgttgttatcttgtttattgc
agcttataatggttacaaataaagcaatagcatcacaaatttcacaaataaagcatttttttcactgcattctagttgtggtttgtccaatctcat
caatgtatcttatcgcgtaaattgtaagcgttaatattttgttaaaattcgcgttaaatttttgttaaatcagctcattttttatccaataggccgaa
atcgggcgcgccgacccagctttCTTGTACAAAGTGGTTGATATCGGTAAGCCTATCCCTAACCCTCTCCTCGGTCTC
GATTCTACGTAGTAATGAACTAGTACCGGTTAAGTCGACAATCAACGCGTTAAGTCGACAATCAACCTCTGGA
TTACAAAATTTGTGAAAGATTGACTGGTATTCTTAACTATGTTGCTCCTTTTACGCTATGTGGATACGCTGCTT
TAATGCCTTTGTATCATGCTATTGCTTCCCGTATGGCTTTCATTTTCTCCTCCTTGTATAAATCCTGGTTGCTGTC
TCTTTATGAGGAGTTGTGGCCCGTTGTCAGGCAACGTGGCGTGGTGTGCACTGTGTTTGCTGACGCAACCC
CCACTGGTTGGGGCATTGCCACCACCTGTCAGCTCCTTTCCGGGACTTTCGCTTTCCCCCTCCCTATTGCCAC
GGCGGAACTCATCGCCGCCTGCCTTGCCCGCTGCTGGACAGGGGCTCGGCTGTTGGGCACTGACAATTCCG
TGGTGTTGTCGGGGAAATCATCGTCCTTTCCTTGGCTGCTCGCCTGTGTTGCCACCTGGATTCTGCGCGGGA
CGTCCTTCTGCTACGTCCCTTCGGCCCTCAATCCAGCGGACCTTCCTTCCCGCGGCCTGCTGCCGGCTCTGCG

GCCTCTTCCGCGTCTTCGCCTTCGCCCTCAGACGAGTCGGATCTCCCTTTGGGCCGCCTCCCCGCGTCGACT
TTAAGACCAATGACTTACAAGGCAGCTGTAGATCTTAGCCACTTTTTAAAAGAAAAGGGGGGACTGGAAGG
GCTAATTCACTCCCAACGAAGACAAGATCTGCTTTTTGCTTGTACTGGGTCTCTCTGGTTAGACCAGATCTGA
GCCTGGGAGCTCTCTGGCTAACTAGGGAACCCACTGCTTAAGCCTCAATAAAGCTTGCCTTGAGTGCTTCAA
GTAGTGTGTGCCCGTCTGTTGTGTGACTCTGGTAACTAGAGATCCCTCAGACCCTTTTAGTCAGTGTGGAAA
ATCTCTAGCAGTACGTATAGTAGTTCATGTCATCTTATTATTCAGTATTTATAACTTGCAAAGAAATGAATATCA
GAGAGTGAGAGGAACTTGTTTATTGCAGCTTATAATGGTTACAAATAAAGCAATAGCATCACAAATTTCACAA
ATAAAGCATTTTTTTCACTGCATTCTAGTTGTGGTTTGTCCAAACTCATCAATGTATCTTATCATGTCTGGCTCT
AGCTATCCCGCCCCTAACTCCGCCCATCCCGCCCCTAACTCCGCCCAGTTCCGCCCATTCTCCGCCCCATGGCT
GACTAATTTTTTTTATTTATGCAGAGGCCGAGGCCGCCTCGGCCTCTGAGCTATTCCAGAAGTAGTGAGGAG
GCTTTTTTGGAGGCCTAGGGACGTACCCAATTCGCCCTATAGTGAGTCGTATTACGCGCGCTCACTGGCCGT
CGTTTTACAACGTCGTGACTGGGAAAACCCTGGCGTTACCCAACTTAATCGCCTTGCAGCACATCCCCCTTTC
GCCAGCTGGCGTAATAGCGAAGAGGCCCGCACCGATCGCCCTTCCCAACAGTTGCGCAGCCTGAATGGCGA
ATGGGACGCGCCCTGTAGCGGCGCATTAAGCGCGGCGGGTGTGGTGGTTACGCGCAGCGTGACCGCTACA
CTTGCCAGCGCCCTAGCGCCCGCTCCTTTCGCTTTCTTCCCTTCCTTTCTCGCCACGTTCGCCGGCTTTCCCC
GTCAAGCTCTAAATCGGGGGCTCCCTTTAGGGTTCCGATTTAGTGCTTTACGGCACCTCGACCCCAAAAAAC
TTGATTAGGGTGATGGTTCACGTAGTGGGCCATCGCCCTGATAGACGGTTTTTCGCCCTTTGACGTTGGAGT
CCACGTTCTTTAATAGTGGACTCTTGTTCCAAACTGGAACAACACTCAACCCTATCTCGGTCTATTCTTTTGAT
TTATAAGGGATTTTGCCGATTTCGGCCTATTGGTTAAAAAATGAGCTGATTTAACAAAAATTTAACGCGAATT
TTAACAAAATATTAACGCTTACAATTTAGGTGGCACTTTTCGGGGAAATGTGCGCGGAACCCCTATTTGTTTA
TTTTTCTAAATACATTCAAATATGTATCCGCTCATGAGACAATAACCCTGATAAATGCTTCAATAATATTGAAAA
AGGAAGAGTATGAGTATTCAACATTTCCGTGTCGCCCTTATTCCCTTTTTTGCGGCATTTTGCCTTCCTGTTTT
TGCTCACCCAGAAACGCTGGTGAAAGTAAAAGATGCTGAAGATCAGTTGGGTGCACGAGTGGGTTACATCG
AACTGGATCTCAACAGCGGTAAGATCCTTGAGAGTTTTCGCCCCGAAGAACGTTTTCCAATGATGAGCACTT
TTAAAGTTCTGCTATGTGGCGCGGTATTATCCCGTATTGACGCCGGGCAAGAGCAACTCGGTCGCCGCATAC
ACTATTCTCAGAATGACTTGGTTGAGTACTCACCAGTCACAGAAAAGCATCTTACGGATGGCATGACAGTAA
GAGAATTATGCAGTGCTGCCATAACCATGAGTGATAACACTGCGGCCAACTTACTTCTGACAACGATCGGAG
GACCGAAGGAGCTAACCGCTTTTTTGCACAACATGGGGGATCATGTAACTCGCCTTGATCGTTGGGAACCG
GAGCTGAATGAAGCCATACCAAACGACGAGCGTGACACCACGATGCCTGTAGCAATGGCAACAACGTTGCG
CAAACTATTAACTGGCGAACTACTTACTCTAGCTTCCCGGCAACAATTAATAGACTGGATGGAGGCGGATAA
AGTTGCAGGACCACTTCTGCGCTCGGCCCTTCCGGCTGGCTGGTTTATTGCTGATAAATCTGGAGCCGGTGA
GCGTGGGTCTCGCGGTATCATTGCAGCACTGGGGCCAGATGGTAAGCCCTCCCGTATCGTAGTTATCTACAC
GACGGGGAGTCAGGCAACTATGGATGAACGAAATAGACAGATCGCTGAGATAGGTGCCTCACTGATTAAGC
ATTGGTAACTGTCAGACCAAGTTTACTCATATATACTTTAGATTGATTTAAAACTTCATTTTTAATTTAAAAGGA
TCTAGGTGAAGATCCTTTTTGATAATCTCATGACCAAAATCCCTTAACGTGAGTTTTCGTTCCACTGAGCGTC
AGACCCCGTAGAAAAGATCAAAGGATCTTCTTGAGATCCTTTTTTTCTGCGCGTAATCTGCTGCTTGCAAACA
AAAAAACCACCGCTACCAGCGGTGGTTTGTTTGCCGGATCAAGAGCTACCAACTCTTTTTCCGAAGGTAACT
GGCTTCAGCAGAGCGCAGATACCAAATACTGTTCTTCTAGTGTAGCCGTAGTTAGGCCACCACTTCAAGAAC
TCTGTAGCACCGCCTACATACCTCGCTCTGCTAATCCTGTTACCAGTGGCTGCTGCCAGTGGCGATAAGTCGT
GTCTTACCGGGTTGGACTCAAGACGATAGTTACCGGATAAGGCGCAGCGGTCGGGCTGAACGGGGGGTTC
GTGCACACAGCCCAGCTTGGAGCGAACGACCTACACCGAACTGAGATACCTACAGCGTGAGCTATGAGAAA
GCGCCACGCTTCCCGAAGGGAGAAAGGCGGACAGGTATCCGGTAAGCGGCAGGGTCGGAACAGGAGAG
CGCACGAGGGAGCTTCCAGGGGGAAACGCCTGGTATCTTTATAGTCCTGTCGGGTTTCGCCACCTCTGACTT
GAGCGTCGATTTTTGTGATGCTCGTCAGGGGGGCGGAGCCTATGGAAAAACGCCAGCAACGCGGCCTTTT

TACGGTTCCTGGCCTTTTGCTGGCCTTTTGCTCACATGTTCTTTCCTGCGTTATCCCCTGATTCTGTGGATAAC
CGTATTACCGCCTTTGAGTGAGCTGATACCGCTCGCCGCAGCCGAACGACCGAGCGCAGCGAGTCAGTGAG
CGAGGAAGCGGAAGAGCGCCCAATACGCAAACCGCCTCTCCCCGCGCGTTGGCCGATTCATTAATGCAGCT
GGCACGACAGGTTTCCCGACTGGAAAGCGGGCAGTGAGCGCAACGCAATTAATGTGAGTTAGCTCACTCAT
TAGGCACCCCAGGCTTTACACTTTATGCTTCCGGCTCGTATGTTGTGTGGAATTGTGAGCGGATAACAATTTC
ACACAGGAAACAGCTATGACCATGATTACGCCAAGCGCGCAATTAACCCTCACTAAAGGGAACAAAAGCTG
GAGCTGCAAGCTTAATGTAGTCTTATGCAATACTCTTGTAGTCTTGCAACATGGTAACGATGAGTTAGCAACA
TGCCTTACAAGGAGAGAAAAAGCACCGTGCATGCCGATTGGTGGAAGTAAGGTGGTACGATCGTGCCTTAT
TAGGAAGGCAACAGACGGGTCTGACATGGATTGGACGAACCACTGAATTGCCGCATTGCAGAGATATTGTA
TTTAAGTGCCTAGCTCGATACATAAACGGGTCTCTCTGGTTAGACCAGATCTGAGCCTGGGAGCTCTCTGGC
TAACTAGGGAACCCACTGCTTAAGCCTCAATAAAGCTTGCCTTGAGTGCTTCAAGTAGTGTGTGCCCGTCTG
TTGTGTGACTCTGGTAACTAGAGATCCCTCAGACCCTTTTAGTCAGTGTGGAAAATCTCTAGCAGTGGCGCC
CGAACAGGGACTTGAAAGCGAAAGGGAAACCAGAGGAGCTCTCTCGACGCAGGACTCGGCTTGCTGAAG
CGCGCACGGCAAGAGGCGAGGGGCGGCGACTGGTGAGTACGCCAAAAATTTTGACTAGCGGAGGCTAGA
AGGAGAGAGATGGGTGCGAGAGCGTCAGTATTAAGCGGGGGAGAATTAGATCGCGATGGGAAAAAATTC
GGTTAAGGCCAGGGGGAAAGAAAAAATATAAATTAAAACATATAGTATGGGCAAGCAGGGAGCTAGAACG
ATTCGCAGTTAATCCTGGCCTGTTAGAAACATCAGAAGGCTGTAGACAAATACTGGGACAGCTACAACCATC
CCTTCAGACAGGATCAGAAGAACTTAGATCATTATATAATACAGTAGCAACCCTCTATTGTGTGCATCAAAGG
ATAGAGATAAAAGACACCAAGGAAGCTTTAGACAAGATAGAGGAAGAGCAAAACAAAAGTAAGACCACCG
CACAGCAAGCGGCCGCTGATCTTCAGACCTGGAGGAGGAGATATGAGGGACAATTGGAGAAGTGAATTAT
ATAAATATAAAGTAGTAAAAATTGAACCATTAGGAGTAGCACCCACCAAGGCAAAGAGAAGAGTGGTGCAG
AGAGAAAAAAGAGCAGTGGGAATAGGAGCTTTGTTCCTTGGGTTCTTGGGAGCAGCAGGAAGCACTATGG
GCGCAGCGTCAATGACGCTGACGGTACAGGCCAGACAATTATTGTCTGGTATAGTGCAGCAGCAGAACAAT
TTGCTGAGGGCTATTGAGGCGCAACAGCATCTGTTGCAACTCACAGTCTGGGGCATCAAGCAGCTCCAGGC
AAGAATCCTGGCTGTGGAAAGATACCTAAAGGATCAACAGCTCCTGGGGATTTGGGGTTGCTCTGGAAAAC
TCATTTGCACCACTGCTGTGCCTTGGAATGCTAGTTGGAGTAATAAATCTCTGGAACAGATTTGGAATCACAC
GACCTGGATGGAGTGGGACAGAGAAATTAACAATTACACAAGCTTAATACACTCCTTAATTGAAGAATCGCA
AAACCAGCAAGAAAAGAATGAACAAGAATTATTGGAATTAGATAAATGGGCAAGTTTGTGGAATTGGTTTA
ACATAACAAATTGGCTGTGGTATATAAAATTATTCATAATGATAGTAGGAGGCTTGGTAGGTTTAAGAATAGTT
TTTGCTGTACTTTCTATAGTGAATAGAGTTAGGCAGGGATATTCACCATTATCGTTTCAGACCCACCTCCCAAC
CCCGAGGGGACCCATGCATTGCATCTCAATTAGTCAGCAACCAGGTGTGGAAAGTCCCCAGGCTCCCCAGC
AGGCAGAAGTATGCAAAGCATGCGTCTCAATTAGTCAGCAACCATAGTCCCGCCCCTAACTCCGCCCATCCC
GCCCCTAACTCCGCCCAGTTCCGCCCATTCTCCGCCCCATGGCTGACTAATTTTTTTTATTTATGCAGAGGCCG
AGGCCGCCTCGGCCTCTGAGCTATTCCAGAAGTAGTGAGGAGGCTTTTTTGGAGGCCTAGGCTTTTGCAAA
AAGCTTTCTAGAGGTACCACCGGAGCTTACCATGACCGAGTACAAGCCCACGGTGCGCCTCGCCACCCGCG
ACGACGTCCCCAGGGCCGTACGCACCCTCGCCGCCGCGTTCGCCGACTACCCCGCCACGCGCCACACCGTC
GATCCGGACCGCCACATCGAGCGGGTCACCGAGCTGCAAGAACTCTTCCTCACGCGCGTCGGGCTCGACAT
CGGCAAGGTGTGGGTCGCGGACGACGGCGCCGCCGTGGCGGTCTGGACCACGCCGGAGAGCGTCGAAG
CGGGGGCGGTGTTCGCCGAGATCGGCCCGCGCATGGCCGAGTTGAGCGGTTCCCGGCTGGCCGCGCAGC
AACAGATGGAAGGCCTCCTGGCGCCGCACCGGCCCAAGGAGCCCGCGTGGTTCCTGGCCACCGTCGGCGT
CTCGCCCGACCACCAGGGCAAGGGTCTGGGCAGCGCCGTCGTGCTCCCCGGAGTGGAGGCGGCCGAGCG
CGCCGGGGTGCCCGCCTTCCTGGAGACCTCCGCGCCCCGCAACCTCCCCTTCTACGAGCGGCTCGGCTTCA
CCGTCACCGCCGACGTCGAGGTGCCCGAAGGACCGCGCACCTGGTGCATGACCCGCAAGCCCGGTGCCAG
TTAACGAATTCTAGATCTTGAGACAAATGGCAGTATTCATCCACAATTTTAAAAGAAAAGGGGGGATTGGGG

GGTACAGTGCAGGGGAAAGAATAGTAGACATAATAGCAACAGACATACAAACTAAAGAATTACAAAAACAA
ATTACAAAAATTCAAAATTTTCGGGTTTATTACAGGGACAGCAGAGATCCACTTTGGCGCCGG

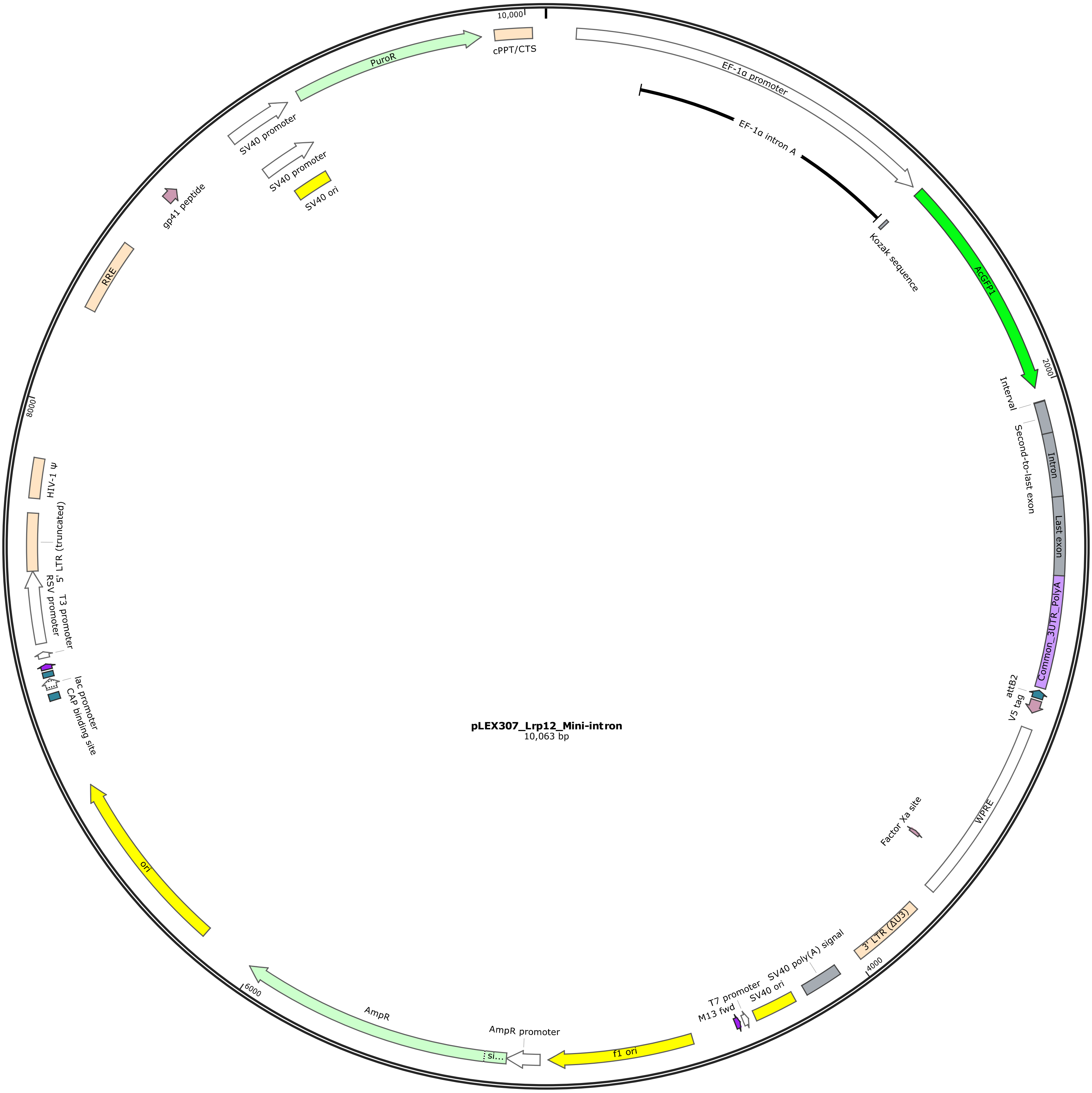
pLEX307_Lrp12_Mini-intron
10,063 bp
10,000
cPPT/CTS
EF-1α promoter
EF-1α intron A
Kozak sequence
AcGFP1
2000
Interval
Second-to-last exon
Intron
Last exon
Common_3UTR_PolyA
attB2
V5 tag
WPRE
Factor Xa site
3' LTR (ΔU3)
4000
SV40 poly(A) signal
SV40 ori
T7 promoter
M13 fwd
f1 ori
AmpR promoter
si...
AmpR
6000
ori
CAP binding site
lac promoter
T3 promoter
RSV promoter
5' LTR (truncated)
HIV-1 ψ
8000
RRE
gp41 peptide
SV40 ori
SV40 promoter
SV40 promoter
PuroR

CTCGAGGCCTGCAGGTGCAAAGATGGATAAAGTTTTAAACAGAGAGGAATCTTTGCAGCTAATGGACCTTC
TAGGTCTTGAAAGGAGTGGGAATTGGCTCCGGTGCCCGTCAGTGGGCAGAGCGCACATCGCCCACAGTCC
CCGAGAAGTTGGGGGGAGGGGTCGGCAATTGAACCGGTGCCTAGAGAAGGTGGCGCGGGGTAAACTGG
GAAAGTGATGTCGTGTACTGGCTCCGCCTTTTTCCCGAGGGTGGGGGAGAACCGTATATAAGTGCAGTAGT
CGCCGTGAACGTTCTTTTTCGCAACGGGTTTGCCGCCAGAACACAGGTAAGTGCCGTGTGTGGTTCCCGCG
GGCCTGGCCTCTTTACGGGTTATGGCCCTTGCGTGCCTTGAATTACTTCCACCTGGCTGCAGTACGTGATTCT
TGATCCCGAGCTTCGGGTTGGAAGTGGGTGGGAGAGTTCGAGGCCTTGCGCTTAAGGAGCCCCTTCGCCT
CGTGCTTGAGTTGAGGCCTGGCCTGGGCGCTGGGGCCGCCGCGTGCGAATCTGGTGGCACCTTCGCGCCT
GTCTCGCTGCTTTCGATAAGTCTCTAGCCATTTAAAATTTTTGATGACCTGCTGCGACGCTTTTTTTCTGGCAA
GATAGTCTTGTAAATGCGGGCCAAGATCTGCACACTGGTATTTCGGTTTTTGGGGCCGCGGGCGGCGACGG
GGCCCGTGCGTCCCAGCGCACATGTTCGGCGAGGCGGGGCCTGCGAGCGCGGCCACCGAGAATCGGACG
GGGGTAGTCTCAAGCTGGCCGGCCTGCTCTGGTGCCTGGCCTCGCGCCGCCGTGTATCGCCCCGCCCTGGG
CGGCAAGGCTGGCCCGGTCGGCACCAGTTGCGTGAGCGGAAAGATGGCCGCTTCCCGGCCCTGCTGCAG
GGAGCTCAAAATGGAGGACGCGGCGCTCGGGAGAGCGGGCGGGTGAGTCACCCACACAAAGGAAAAGG
GCCTTTCCGTCCTCAGCCGTCGCTTCATGTGACTCCACGGAGTACCGGGCGCCGTCCAGGCACCTCGATTAG
TTCTCGAGCTTTTGGAGTACGTCGTCTTTAGGTTGGGGGGAGGGGTTTTATGCGATGGAGTTTCCCCACACT
GAGTGGGTGGAGACTGAAGTTAGGCCAGCTTGGCACTTGATGTAATTCTCCTTGGAATTTGCCCTTTTTGAG
TTTGGATCTTGGTTCATTCTCAAGCCTCAGACAGTGGTTCAAAGTTTTTTTCTTCCATTTCAGGTGTCGTGAG
GCTAGCgctaccggtcgccaccatggtgagcaagggcgccgagctgttcaccggcatcgtgcccatcctgatcgagctgaatggcgatgt
gaatggccacaagttcagcgtgagcggcgagggcgagggcgatgccacctacggcaagcttaccctgaagttcatctgcaccaccggcaa
gctgcctgtgccctggcccaccctggttaccaccctgagctacggcgtgcagtgcttctcacgctaccccgatcacatgaagcagcacgatttc
ttcaagagcgccatgcctgagggctacatccaggagcgcaccatcttcttcgaggatgatggcaattacaagtcgcgcgccgaggtgaagtt
cgagggcgataccctggtgaatcgcatcgagcttaccggcaccgatttcaaggaggatggcaatatcctgggcaataagatggagtacaatt
acaatgcccacaatgtgtacatcatgacggataaggccaagaatggcatcaaggtgaatttcaagatccgccacaatatcgaggatggcag
cgtgcagctggccgatcactaccagcagaatacccccatcggcgatggccctgtgctgctgcccgataatcactacctgtccacccagagcg
ccctgtccaaggatcccaatgagaagcgcgatcacatgatctacttcggcttcgttaccgccgccgccatcacccacggcatggatgagctgt
acaagtccgggctcagatctcgagctcaagcttcgaattctgcagtcgatAAAGAACTTCTCCGAAGAGAGGCTCCTCCCTCA
TATGGACAGTTGATCGCTCAGGGACTAATCCCACCCGTAGAAGATTTTCCTGTCTGTTCACCTAATCAGGTAT
AATGAGAATTCTAATCTGTTTCATCCTCTTTTAAAGTCTATAAGTAGTAAAGCAGGGATTCTTAACTTTAGGAG
TTTTCACAATCATGCAAATATTTTAATTTAGCTCATCGCTGTTCCTAGTAACAGTGAGTCAAAGAATGTTTTTC
ATCTTAATACCTCATTATTCACTATTTTCTTTTTTCTTACTCTTGCAGGCTTCCGTTTTAGAAAACCTGAGGCTA
GCTGTCCGATCTCAGCTGGGATTTACTTCAATCAGGCTTCCTATGACAGGCAGATCTAGCAACATTTGGAATC
GTATTTTTAATTTTGCAAGATCCCGTCATTCAGGGTCATTGGCTTTGGTCTCGGGAGATGGAGATGAGGTTG
TCCCTAGCCAGAGCAGCAGCAGAGAAACTGAGAGGAGTCGCCCTCACAGAAGCTTATTCTCCGTGGAGTCC
GATgtaccgcgggcccgggatccaccggatctagatatctgatcataatcagccataccacatttgtagaggttttacttgctttaaaaatcct
cccacacctccccctgatcctgaatcataaaatgaatgcaattgttgttgttatcttgtttattgcagcttataatggttacaaataaagcaatag
catcacaaatttcacaaataaagcatttttttcactgcattctagttgtggtttgtccaatctcatcaatgtatcttatcgcgtaaattgtaagcgt
taatattttgttaaaattcgcgttaaatttttgttaaatcagctcattttttatccaataggccgaaatcgggcgcgccgacccagctttCTTGT
ACAAAGTGGTTGATATCGGTAAGCCTATCCCTAACCCTCTCCTCGGTCTCGATTCTACGTAGTAATGAACTAGT
ACCGGTTAAGTCGACAATCAACGCGTTAAGTCGACAATCAACCTCTGGATTACAAAATTTGTGAAAGATTGA
CTGGTATTCTTAACTATGTTGCTCCTTTTACGCTATGTGGATACGCTGCTTTAATGCCTTTGTATCATGCTATTGC
TTCCCGTATGGCTTTCATTTTCTCCTCCTTGTATAAATCCTGGTTGCTGTCTCTTTATGAGGAGTTGTGGCCCGT
TGTCAGGCAACGTGGCGTGGTGTGCACTGTGTTTGCTGACGCAACCCCCACTGGTTGGGGCATTGCCACCA
CCTGTCAGCTCCTTTCCGGGACTTTCGCTTTCCCCCTCCCTATTGCCACGGCGGAACTCATCGCCGCCTGCCT

TGCCCGCTGCTGGACAGGGGCTCGGCTGTTGGGCACTGACAATTCCGTGGTGTTGTCGGGGAAATCATCGT
CCTTTCCTTGGCTGCTCGCCTGTGTTGCCACCTGGATTCTGCGCGGGACGTCCTTCTGCTACGTCCCTTCGGC
CCTCAATCCAGCGGACCTTCCTTCCCGCGGCCTGCTGCCGGCTCTGCGGCCTCTTCCGCGTCTTCGCCTTCG
CCCTCAGACGAGTCGGATCTCCCTTTGGGCCGCCTCCCCGCGTCGACTTTAAGACCAATGACTTACAAGGCA
GCTGTAGATCTTAGCCACTTTTTAAAAGAAAAGGGGGGACTGGAAGGGCTAATTCACTCCCAACGAAGACA
AGATCTGCTTTTTGCTTGTACTGGGTCTCTCTGGTTAGACCAGATCTGAGCCTGGGAGCTCTCTGGCTAACTA
GGGAACCCACTGCTTAAGCCTCAATAAAGCTTGCCTTGAGTGCTTCAAGTAGTGTGTGCCCGTCTGTTGTGT
GACTCTGGTAACTAGAGATCCCTCAGACCCTTTTAGTCAGTGTGGAAAATCTCTAGCAGTACGTATAGTAGTT
CATGTCATCTTATTATTCAGTATTTATAACTTGCAAAGAAATGAATATCAGAGAGTGAGAGGAACTTGTTTATT
GCAGCTTATAATGGTTACAAATAAAGCAATAGCATCACAAATTTCACAAATAAAGCATTTTTTTCACTGCATTC
TAGTTGTGGTTTGTCCAAACTCATCAATGTATCTTATCATGTCTGGCTCTAGCTATCCCGCCCCTAACTCCGCCC
ATCCCGCCCCTAACTCCGCCCAGTTCCGCCCATTCTCCGCCCCATGGCTGACTAATTTTTTTTATTTATGCAGA
GGCCGAGGCCGCCTCGGCCTCTGAGCTATTCCAGAAGTAGTGAGGAGGCTTTTTTGGAGGCCTAGGGACG
TACCCAATTCGCCCTATAGTGAGTCGTATTACGCGCGCTCACTGGCCGTCGTTTTACAACGTCGTGACTGGGA
AAACCCTGGCGTTACCCAACTTAATCGCCTTGCAGCACATCCCCCTTTCGCCAGCTGGCGTAATAGCGAAGA
GGCCCGCACCGATCGCCCTTCCCAACAGTTGCGCAGCCTGAATGGCGAATGGGACGCGCCCTGTAGCGGC
GCATTAAGCGCGGCGGGTGTGGTGGTTACGCGCAGCGTGACCGCTACACTTGCCAGCGCCCTAGCGCCCGC
TCCTTTCGCTTTCTTCCCTTCCTTTCTCGCCACGTTCGCCGGCTTTCCCCGTCAAGCTCTAAATCGGGGGCTCC
CTTTAGGGTTCCGATTTAGTGCTTTACGGCACCTCGACCCCAAAAAACTTGATTAGGGTGATGGTTCACGTA
GTGGGCCATCGCCCTGATAGACGGTTTTTCGCCCTTTGACGTTGGAGTCCACGTTCTTTAATAGTGGACTCTT
GTTCCAAACTGGAACAACACTCAACCCTATCTCGGTCTATTCTTTTGATTTATAAGGGATTTTGCCGATTTCGG
CCTATTGGTTAAAAAATGAGCTGATTTAACAAAAATTTAACGCGAATTTTAACAAAATATTAACGCTTACAATT
TAGGTGGCACTTTTCGGGGAAATGTGCGCGGAACCCCTATTTGTTTATTTTTCTAAATACATTCAAATATGTAT
CCGCTCATGAGACAATAACCCTGATAAATGCTTCAATAATATTGAAAAAGGAAGAGTATGAGTATTCAACATT
TCCGTGTCGCCCTTATTCCCTTTTTTGCGGCATTTTGCCTTCCTGTTTTTGCTCACCCAGAAACGCTGGTGAA
AGTAAAAGATGCTGAAGATCAGTTGGGTGCACGAGTGGGTTACATCGAACTGGATCTCAACAGCGGTAAGA
TCCTTGAGAGTTTTCGCCCCGAAGAACGTTTTCCAATGATGAGCACTTTTAAAGTTCTGCTATGTGGCGCGG
TATTATCCCGTATTGACGCCGGGCAAGAGCAACTCGGTCGCCGCATACACTATTCTCAGAATGACTTGGTTGA
GTACTCACCAGTCACAGAAAAGCATCTTACGGATGGCATGACAGTAAGAGAATTATGCAGTGCTGCCATAAC
CATGAGTGATAACACTGCGGCCAACTTACTTCTGACAACGATCGGAGGACCGAAGGAGCTAACCGCTTTTTT
GCACAACATGGGGGATCATGTAACTCGCCTTGATCGTTGGGAACCGGAGCTGAATGAAGCCATACCAAACG
ACGAGCGTGACACCACGATGCCTGTAGCAATGGCAACAACGTTGCGCAAACTATTAACTGGCGAACTACTTA
CTCTAGCTTCCCGGCAACAATTAATAGACTGGATGGAGGCGGATAAAGTTGCAGGACCACTTCTGCGCTCG
GCCCTTCCGGCTGGCTGGTTTATTGCTGATAAATCTGGAGCCGGTGAGCGTGGGTCTCGCGGTATCATTGCA
GCACTGGGGCCAGATGGTAAGCCCTCCCGTATCGTAGTTATCTACACGACGGGGAGTCAGGCAACTATGGAT
GAACGAAATAGACAGATCGCTGAGATAGGTGCCTCACTGATTAAGCATTGGTAACTGTCAGACCAAGTTTAC
TCATATATACTTTAGATTGATTTAAAACTTCATTTTTAATTTAAAAGGATCTAGGTGAAGATCCTTTTTGATAATC
TCATGACCAAAATCCCTTAACGTGAGTTTTCGTTCCACTGAGCGTCAGACCCCGTAGAAAAGATCAAAGGAT
CTTCTTGAGATCCTTTTTTTCTGCGCGTAATCTGCTGCTTGCAAACAAAAAAACCACCGCTACCAGCGGTGG
TTTGTTTGCCGGATCAAGAGCTACCAACTCTTTTTCCGAAGGTAACTGGCTTCAGCAGAGCGCAGATACCAA
ATACTGTTCTTCTAGTGTAGCCGTAGTTAGGCCACCACTTCAAGAACTCTGTAGCACCGCCTACATACCTCGCT
CTGCTAATCCTGTTACCAGTGGCTGCTGCCAGTGGCGATAAGTCGTGTCTTACCGGGTTGGACTCAAGACGA
TAGTTACCGGATAAGGCGCAGCGGTCGGGCTGAACGGGGGGTTCGTGCACACAGCCCAGCTTGGAGCGA
ACGACCTACACCGAACTGAGATACCTACAGCGTGAGCTATGAGAAAGCGCCACGCTTCCCGAAGGGAGAA

AGGCGGACAGGTATCCGGTAAGCGGCAGGGTCGGAACAGGAGAGCGCACGAGGGAGCTTCCAGGGGGA
AACGCCTGGTATCTTTATAGTCCTGTCGGGTTTCGCCACCTCTGACTTGAGCGTCGATTTTTGTGATGCTCGTC
AGGGGGGCGGAGCCTATGGAAAAACGCCAGCAACGCGGCCTTTTTACGGTTCCTGGCCTTTTGCTGGCCT
TTTGCTCACATGTTCTTTCCTGCGTTATCCCCTGATTCTGTGGATAACCGTATTACCGCCTTTGAGTGAGCTGA
TACCGCTCGCCGCAGCCGAACGACCGAGCGCAGCGAGTCAGTGAGCGAGGAAGCGGAAGAGCGCCCAAT
ACGCAAACCGCCTCTCCCCGCGCGTTGGCCGATTCATTAATGCAGCTGGCACGACAGGTTTCCCGACTGGA
AAGCGGGCAGTGAGCGCAACGCAATTAATGTGAGTTAGCTCACTCATTAGGCACCCCAGGCTTTACACTTTA
TGCTTCCGGCTCGTATGTTGTGTGGAATTGTGAGCGGATAACAATTTCACACAGGAAACAGCTATGACCATG
ATTACGCCAAGCGCGCAATTAACCCTCACTAAAGGGAACAAAAGCTGGAGCTGCAAGCTTAATGTAGTCTTA
TGCAATACTCTTGTAGTCTTGCAACATGGTAACGATGAGTTAGCAACATGCCTTACAAGGAGAGAAAAAGCA
CCGTGCATGCCGATTGGTGGAAGTAAGGTGGTACGATCGTGCCTTATTAGGAAGGCAACAGACGGGTCTGA
CATGGATTGGACGAACCACTGAATTGCCGCATTGCAGAGATATTGTATTTAAGTGCCTAGCTCGATACATAAA
CGGGTCTCTCTGGTTAGACCAGATCTGAGCCTGGGAGCTCTCTGGCTAACTAGGGAACCCACTGCTTAAGCC
TCAATAAAGCTTGCCTTGAGTGCTTCAAGTAGTGTGTGCCCGTCTGTTGTGTGACTCTGGTAACTAGAGATCC
CTCAGACCCTTTTAGTCAGTGTGGAAAATCTCTAGCAGTGGCGCCCGAACAGGGACTTGAAAGCGAAAGG
GAAACCAGAGGAGCTCTCTCGACGCAGGACTCGGCTTGCTGAAGCGCGCACGGCAAGAGGCGAGGGGC
GGCGACTGGTGAGTACGCCAAAAATTTTGACTAGCGGAGGCTAGAAGGAGAGAGATGGGTGCGAGAGCG
TCAGTATTAAGCGGGGGAGAATTAGATCGCGATGGGAAAAAATTCGGTTAAGGCCAGGGGGAAAGAAAAA
ATATAAATTAAAACATATAGTATGGGCAAGCAGGGAGCTAGAACGATTCGCAGTTAATCCTGGCCTGTTAGAA
ACATCAGAAGGCTGTAGACAAATACTGGGACAGCTACAACCATCCCTTCAGACAGGATCAGAAGAACTTAG
ATCATTATATAATACAGTAGCAACCCTCTATTGTGTGCATCAAAGGATAGAGATAAAAGACACCAAGGAAGCT
TTAGACAAGATAGAGGAAGAGCAAAACAAAAGTAAGACCACCGCACAGCAAGCGGCCGCTGATCTTCAGA
CCTGGAGGAGGAGATATGAGGGACAATTGGAGAAGTGAATTATATAAATATAAAGTAGTAAAAATTGAACCA
TTAGGAGTAGCACCCACCAAGGCAAAGAGAAGAGTGGTGCAGAGAGAAAAAAGAGCAGTGGGAATAGG
AGCTTTGTTCCTTGGGTTCTTGGGAGCAGCAGGAAGCACTATGGGCGCAGCGTCAATGACGCTGACGGTAC
AGGCCAGACAATTATTGTCTGGTATAGTGCAGCAGCAGAACAATTTGCTGAGGGCTATTGAGGCGCAACAG
CATCTGTTGCAACTCACAGTCTGGGGCATCAAGCAGCTCCAGGCAAGAATCCTGGCTGTGGAAAGATACCT
AAAGGATCAACAGCTCCTGGGGATTTGGGGTTGCTCTGGAAAACTCATTTGCACCACTGCTGTGCCTTGGA
ATGCTAGTTGGAGTAATAAATCTCTGGAACAGATTTGGAATCACACGACCTGGATGGAGTGGGACAGAGAA
ATTAACAATTACACAAGCTTAATACACTCCTTAATTGAAGAATCGCAAAACCAGCAAGAAAAGAATGAACAA
GAATTATTGGAATTAGATAAATGGGCAAGTTTGTGGAATTGGTTTAACATAACAAATTGGCTGTGGTATATAA
AATTATTCATAATGATAGTAGGAGGCTTGGTAGGTTTAAGAATAGTTTTTGCTGTACTTTCTATAGTGAATAGA
GTTAGGCAGGGATATTCACCATTATCGTTTCAGACCCACCTCCCAACCCCGAGGGGACCCATGCATTGCATCT
CAATTAGTCAGCAACCAGGTGTGGAAAGTCCCCAGGCTCCCCAGCAGGCAGAAGTATGCAAAGCATGCGTC
TCAATTAGTCAGCAACCATAGTCCCGCCCCTAACTCCGCCCATCCCGCCCCTAACTCCGCCCAGTTCCGCCCAT
TCTCCGCCCCATGGCTGACTAATTTTTTTTATTTATGCAGAGGCCGAGGCCGCCTCGGCCTCTGAGCTATTCC
AGAAGTAGTGAGGAGGCTTTTTTGGAGGCCTAGGCTTTTGCAAAAAGCTTTCTAGAGGTACCACCGGAGC
TTACCATGACCGAGTACAAGCCCACGGTGCGCCTCGCCACCCGCGACGACGTCCCCAGGGCCGTACGCACC
CTCGCCGCCGCGTTCGCCGACTACCCCGCCACGCGCCACACCGTCGATCCGGACCGCCACATCGAGCGGGT
CACCGAGCTGCAAGAACTCTTCCTCACGCGCGTCGGGCTCGACATCGGCAAGGTGTGGGTCGCGGACGAC
GGCGCCGCCGTGGCGGTCTGGACCACGCCGGAGAGCGTCGAAGCGGGGGCGGTGTTCGCCGAGATCGGC
CCGCGCATGGCCGAGTTGAGCGGTTCCCGGCTGGCCGCGCAGCAACAGATGGAAGGCCTCCTGGCGCCGC
ACCGGCCCAAGGAGCCCGCGTGGTTCCTGGCCACCGTCGGCGTCTCGCCCGACCACCAGGGCAAGGGTCT
GGGCAGCGCCGTCGTGCTCCCCGGAGTGGAGGCGGCCGAGCGCGCCGGGGTGCCCGCCTTCCTGGAGAC

CTCCGCGCCCCGCAACCTCCCCTTCTACGAGCGGCTCGGCTTCACCGTCACCGCCGACGTCGAGGTGCCCG
AAGGACCGCGCACCTGGTGCATGACCCGCAAGCCCGGTGCCAGTTAACGAATTCTAGATCTTGAGACAAAT
GGCAGTATTCATCCACAATTTTAAAAGAAAAGGGGGGATTGGGGGGTACAGTGCAGGGGAAAGAATAGTA
GACATAATAGCAACAGACATACAAACTAAAGAATTACAAAAACAAATTACAAAAATTCAAAATTTTCGGGTTT
ATTACAGGGACAGCAGAGATCCACTTTGGCGCCGG

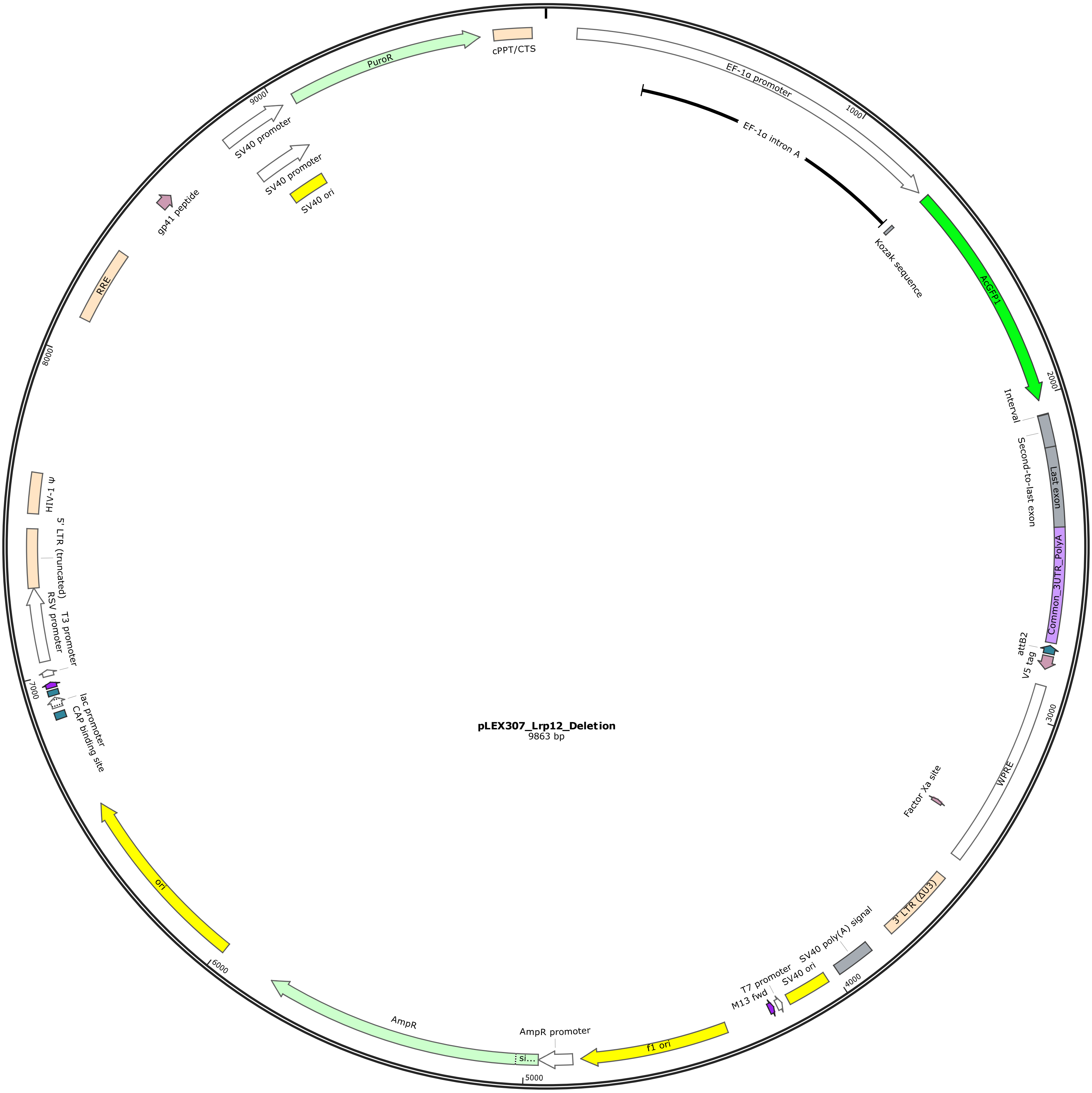
pLEX307_Lrp12_Deletion
9863 bp
cPPT/CTS
PuroR
EF-1α promoter
EF-1α intron A
Kozak sequence
AcGFP1
Interval
Second-to-last exon
Last exon
Common_3UTR_PolyA
attB2
V5 tag
WPRE
Factor Xa site
3' LTR (ΔU3)
SV40 poly(A) signal
SV40 ori
T7 promoter
M13 fwd
f1 ori
AmpR promoter
AmpR
ori
lac promoter
CAP binding site
T3 promoter
RSV promoter
5' LTR (truncated)
HIV-1 Ψ
RRE
gp41 peptide
SV40 promoter
SV40 promoter
SV40 ori
1000
2000
3000
4000
5000
6000
7000
8000
9000

CTCGAGGCCTGCAGGTGCAAAGATGGATAAAGTTTTAAACAGAGAGGAATCTTTGCAGCTAATGGACCTTC
TAGGTCTTGAAAGGAGTGGGAATTGGCTCCGGTGCCCGTCAGTGGGCAGAGCGCACATCGCCCACAGTCC
CCGAGAAGTTGGGGGGAGGGGTCGGCAATTGAACCGGTGCCTAGAGAAGGTGGCGCGGGGTAAACTGG
GAAAGTGATGTCGTGTACTGGCTCCGCCTTTTTCCCGAGGGTGGGGGAGAACCGTATATAAGTGCAGTAGT
CGCCGTGAACGTTCTTTTTCGCAACGGGTTTGCCGCCAGAACACAGGTAAGTGCCGTGTGTGGTTCCCGCG
GGCCTGGCCTCTTTACGGGTTATGGCCCTTGCGTGCCTTGAATTACTTCCACCTGGCTGCAGTACGTGATTCT
TGATCCCGAGCTTCGGGTTGGAAGTGGGTGGGAGAGTTCGAGGCCTTGCGCTTAAGGAGCCCCTTCGCCT
CGTGCTTGAGTTGAGGCCTGGCCTGGGCGCTGGGGCCGCCGCGTGCGAATCTGGTGGCACCTTCGCGCCT
GTCTCGCTGCTTTCGATAAGTCTCTAGCCATTTAAAATTTTTGATGACCTGCTGCGACGCTTTTTTTCTGGCAA
GATAGTCTTGTAAATGCGGGCCAAGATCTGCACACTGGTATTTCGGTTTTTGGGGCCGCGGGCGGCGACGG
GGCCCGTGCGTCCCAGCGCACATGTTCGGCGAGGCGGGGCCTGCGAGCGCGGCCACCGAGAATCGGACG
GGGGTAGTCTCAAGCTGGCCGGCCTGCTCTGGTGCCTGGCCTCGCGCCGCCGTGTATCGCCCCGCCCTGGG
CGGCAAGGCTGGCCCGGTCGGCACCAGTTGCGTGAGCGGAAAGATGGCCGCTTCCCGGCCCTGCTGCAG
GGAGCTCAAAATGGAGGACGCGGCGCTCGGGAGAGCGGGCGGGTGAGTCACCCACACAAAGGAAAAGG
GCCTTTCCGTCCTCAGCCGTCGCTTCATGTGACTCCACGGAGTACCGGGCGCCGTCCAGGCACCTCGATTAG
TTCTCGAGCTTTTGGAGTACGTCGTCTTTAGGTTGGGGGGAGGGGTTTTATGCGATGGAGTTTCCCCACACT
GAGTGGGTGGAGACTGAAGTTAGGCCAGCTTGGCACTTGATGTAATTCTCCTTGGAATTTGCCCTTTTTGAG
TTTGGATCTTGGTTCATTCTCAAGCCTCAGACAGTGGTTCAAAGTTTTTTTCTTCCATTTCAGGTGTCGTGAG
GCTAGCgctaccggtcgccaccatggtgagcaagggcgccgagctgttcaccggcatcgtgcccatcctgatcgagctgaatggcgatgt
gaatggccacaagttcagcgtgagcggcgagggcgagggcgatgccacctacggcaagcttaccctgaagttcatctgcaccaccggcaa
gctgcctgtgccctggcccaccctggttaccaccctgagctacggcgtgcagtgcttctcacgctaccccgatcacatgaagcagcacgatttc
ttcaagagcgccatgcctgagggctacatccaggagcgcaccatcttcttcgaggatgatggcaattacaagtcgcgcgccgaggtgaagtt
cgagggcgataccctggtgaatcgcatcgagcttaccggcaccgatttcaaggaggatggcaatatcctgggcaataagatggagtacaatt
acaatgcccacaatgtgtacatcatgacggataaggccaagaatggcatcaaggtgaatttcaagatccgccacaatatcgaggatggcag
cgtgcagctggccgatcactaccagcagaatacccccatcggcgatggccctgtgctgctgcccgataatcactacctgtccacccagagcg
ccctgtccaaggatcccaatgagaagcgcgatcacatgatctacttcggcttcgttaccgccgccgccatcacccacggcatggatgagctgt
acaagtccgggctcagatctcgagctcaagcttcgaattctgcagtcgatAAAGAACTTCTCCGAAGAGAGGCTCCTCCCTCA
TATGGACAGTTGATCGCTCAGGGACTAATCCCACCCGTAGAAGATTTTCCTGTCTGTTCACCTAATCAGGCTT
CCGTTTTAGAAAACCTGAGGCTAGCTGTCCGATCTCAGCTGGGATTTACTTCAATCAGGCTTCCTATGACAG
GCAGATCTAGCAACATTTGGAATCGTATTTTTAATTTTGCAAGATCCCGTCATTCAGGGTCATTGGCTTTGGTC
TCGGGAGATGGAGATGAGGTTGTCCCTAGCCAGAGCAGCAGCAGAGAAACTGAGAGGAGTCGCCCTCAC
AGAAGCTTATTCTCCGTGGAGTCCGATgtaccgcgggcccgggatccaccggatctagatatctgatcataatcagccataccac
atttgtagaggttttacttgctttaaaaaacctcccacacctccccctgatcctgaatcataaaatgaatgcaattgttgttgttatcttgtttattg
cagcttataatggttacaaataaagcaatagcatcacaaatttcacaaataaagcatttttttcactgcattctagttgtggtttgtccaatctca
tcaatgtatcttatcgcgtaaattgtaagcgttaatattttgttaaaattcgcgttaaatttttgttaaatcagctcattttttatccaataggccga
aatcgggcgcgccgacccagctttCTTGTACAAAGTGGTTGATATCGGTAAGCCTATCCCTAACCCTCTCCTCGGTCTC
GATTCTACGTAGTAATGAACTAGTACCGGTTAAGTCGACAATCAACGCGTTAAGTCGACAATCAACCTCTGGA
TTACAAAATTTGTGAAAGATTGACTGGTATTCTTAACTATGTTGCTCCTTTTACGCTATGTGGATACGCTGCTT
TAATGCCTTTGTATCATGCTATTGCTTCCCGTATGGCTTTCATTTTCTCCTCCTTGTATAAATCCTGGTTGCTGTC
TCTTTATGAGGAGTTGTGGCCCGTTGTCAGGCAACGTGGCGTGGTGTGCACTGTGTTTGCTGACGCAACCC
CCACTGGTTGGGGCATTGCCACCACCTGTCAGCTCCTTTCCGGGACTTTCGCTTTCCCCCTCCCTATTGCCAC
GGCGGAACTCATCGCCGCCTGCCTTGCCCGCTGCTGGACAGGGGCTCGGCTGTTGGGCACTGACAATTCCG
TGGTGTTGTCGGGGAAATCATCGTCCTTTCCTTGGCTGCTCGCCTGTGTTGCCACCTGGATTCTGCGCGGGA
CGTCCTTCTGCTACGTCCCTTCGGCCCTCAATCCAGCGGACCTTCCTTCCCGCGGCCTGCTGCCGGCTCTGCG

GCCTCTTCCGCGTCTTCGCCTTCGCCCTCAGACGAGTCGGATCTCCCTTTGGGCCGCCTCCCCGCGTCGACT
TTAAGACCAATGACTTACAAGGCAGCTGTAGATCTTAGCCACTTTTTAAAAGAAAAGGGGGGACTGGAAGG
GCTAATTCACTCCCAACGAAGACAAGATCTGCTTTTTGCTTGTACTGGGTCTCTCTGGTTAGACCAGATCTGA
GCCTGGGAGCTCTCTGGCTAACTAGGGAACCCACTGCTTAAGCCTCAATAAAGCTTGCCTTGAGTGCTTCAA
GTAGTGTGTGCCCGTCTGTTGTGTGACTCTGGTAACTAGAGATCCCTCAGACCCTTTTAGTCAGTGTGGAAA
ATCTCTAGCAGTACGTATAGTAGTTCATGTCATCTTATTATTCAGTATTTATAACTTGCAAAGAAATGAATATCA
GAGAGTGAGAGGAACTTGTTTATTGCAGCTTATAATGGTTACAAATAAAGCAATAGCATCACAAATTTCACAA
ATAAAGCATTTTTTTCACTGCATTCTAGTTGTGGTTTGTCCAAACTCATCAATGTATCTTATCATGTCTGGCTCT
AGCTATCCCGCCCCTAACTCCGCCCATCCCGCCCCTAACTCCGCCCAGTTCCGCCCATTCTCCGCCCCATGGCT
GACTAATTTTTTTTATTTATGCAGAGGCCGAGGCCGCCTCGGCCTCTGAGCTATTCCAGAAGTAGTGAGGAG
GCTTTTTTGGAGGCCTAGGGACGTACCCAATTCGCCCTATAGTGAGTCGTATTACGCGCGCTCACTGGCCGT
CGTTTTACAACGTCGTGACTGGGAAAACCCTGGCGTTACCCAACTTAATCGCCTTGCAGCACATCCCCCTTTC
GCCAGCTGGCGTAATAGCGAAGAGGCCCGCACCGATCGCCCTTCCCAACAGTTGCGCAGCCTGAATGGCGA
ATGGGACGCGCCCTGTAGCGGCGCATTAAGCGCGGCGGGTGTGGTGGTTACGCGCAGCGTGACCGCTACA
CTTGCCAGCGCCCTAGCGCCCGCTCCTTTCGCTTTCTTCCCTTCCTTTCTCGCCACGTTCGCCGGCTTTCCCC
GTCAAGCTCTAAATCGGGGGCTCCCTTTAGGGTTCCGATTTAGTGCTTTACGGCACCTCGACCCCAAAAAAC
TTGATTAGGGTGATGGTTCACGTAGTGGGCCATCGCCCTGATAGACGGTTTTTCGCCCTTTGACGTTGGAGT
CCACGTTCTTTAATAGTGGACTCTTGTTCCAAACTGGAACAACACTCAACCCTATCTCGGTCTATTCTTTTGAT
TTATAAGGGATTTTGCCGATTTCGGCCTATTGGTTAAAAAATGAGCTGATTTAACAAAAATTTAACGCGAATT
TTAACAAAATATTAACGCTTACAATTTAGGTGGCACTTTTCGGGGAAATGTGCGCGGAACCCCTATTTGTTTA
TTTTTCTAAATACATTCAAATATGTATCCGCTCATGAGACAATAACCCTGATAAATGCTTCAATAATATTGAAAA
AGGAAGAGTATGAGTATTCAACATTTCCGTGTCGCCCTTATTCCCTTTTTTGCGGCATTTTGCCTTCCTGTTTT
TGCTCACCCAGAAACGCTGGTGAAAGTAAAAGATGCTGAAGATCAGTTGGGTGCACGAGTGGGTTACATCG
AACTGGATCTCAACAGCGGTAAGATCCTTGAGAGTTTTCGCCCCGAAGAACGTTTTCCAATGATGAGCACTT
TTAAAGTTCTGCTATGTGGCGCGGTATTATCCCGTATTGACGCCGGGCAAGAGCAACTCGGTCGCCGCATAC
ACTATTCTCAGAATGACTTGGTTGAGTACTCACCAGTCACAGAAAAGCATCTTACGGATGGCATGACAGTAA
GAGAATTATGCAGTGCTGCCATAACCATGAGTGATAACACTGCGGCCAACTTACTTCTGACAACGATCGGAG
GACCGAAGGAGCTAACCGCTTTTTTGCACAACATGGGGGATCATGTAACTCGCCTTGATCGTTGGGAACCG
GAGCTGAATGAAGCCATACCAAACGACGAGCGTGACACCACGATGCCTGTAGCAATGGCAACAACGTTGCG
CAAACTATTAACTGGCGAACTACTTACTCTAGCTTCCCGGCAACAATTAATAGACTGGATGGAGGCGGATAA
AGTTGCAGGACCACTTCTGCGCTCGGCCCTTCCGGCTGGCTGGTTTATTGCTGATAAATCTGGAGCCGGTGA
GCGTGGGTCTCGCGGTATCATTGCAGCACTGGGGCCAGATGGTAAGCCCTCCCGTATCGTAGTTATCTACAC
GACGGGGAGTCAGGCAACTATGGATGAACGAAATAGACAGATCGCTGAGATAGGTGCCTCACTGATTAAGC
ATTGGTAACTGTCAGACCAAGTTTACTCATATATACTTTAGATTGATTTAAAACTTCATTTTTAATTTAAAAGGA
TCTAGGTGAAGATCCTTTTTGATAATCTCATGACCAAAATCCCTTAACGTGAGTTTTCGTTCCACTGAGCGTC
AGACCCCGTAGAAAAGATCAAAGGATCTTCTTGAGATCCTTTTTTTCTGCGCGTAATCTGCTGCTTGCAAACA
AAAAAACCACCGCTACCAGCGGTGGTTTGTTTGCCGGATCAAGAGCTACCAACTCTTTTTCCGAAGGTAACT
GGCTTCAGCAGAGCGCAGATACCAAATACTGTTCTTCTAGTGTAGCCGTAGTTAGGCCACCACTTCAAGAAC
TCTGTAGCACCGCCTACATACCTCGCTCTGCTAATCCTGTTACCAGTGGCTGCTGCCAGTGGCGATAAGTCGT
GTCTTACCGGGTTGGACTCAAGACGATAGTTACCGGATAAGGCGCAGCGGTCGGGCTGAACGGGGGGTTC
GTGCACACAGCCCAGCTTGGAGCGAACGACCTACACCGAACTGAGATACCTACAGCGTGAGCTATGAGAAA
GCGCCACGCTTCCCGAAGGGAGAAAGGCGGACAGGTATCCGGTAAGCGGCAGGGTCGGAACAGGAGAG
CGCACGAGGGAGCTTCCAGGGGGAAACGCCTGGTATCTTTATAGTCCTGTCGGGTTTCGCCACCTCTGACTT
GAGCGTCGATTTTTGTGATGCTCGTCAGGGGGGCGGAGCCTATGGAAAAACGCCAGCAACGCGGCCTTTT

TACGGTTCCTGGCCTTTTGCTGGCCTTTTGCTCACATGTTCTTTCCTGCGTTATCCCCTGATTCTGTGGATAAC
CGTATTACCGCCTTTGAGTGAGCTGATACCGCTCGCCGCAGCCGAACGACCGAGCGCAGCGAGTCAGTGAG
CGAGGAAGCGGAAGAGCGCCCAATACGCAAACCGCCTCTCCCCGCGCGTTGGCCGATTCATTAATGCAGCT
GGCACGACAGGTTTCCCGACTGGAAAGCGGGCAGTGAGCGCAACGCAATTAATGTGAGTTAGCTCACTCAT
TAGGCACCCCAGGCTTTACACTTTATGCTTCCGGCTCGTATGTTGTGTGGAATTGTGAGCGGATAACAATTTC
ACACAGGAAACAGCTATGACCATGATTACGCCAAGCGCGCAATTAACCCTCACTAAAGGGAACAAAAGCTG
GAGCTGCAAGCTTAATGTAGTCTTATGCAATACTCTTGTAGTCTTGCAACATGGTAACGATGAGTTAGCAACA
TGCCTTACAAGGAGAGAAAAAGCACCGTGCATGCCGATTGGTGGAAGTAAGGTGGTACGATCGTGCCTTAT
TAGGAAGGCAACAGACGGGTCTGACATGGATTGGACGAACCACTGAATTGCCGCATTGCAGAGATATTGTA
TTTAAGTGCCTAGCTCGATACATAAACGGGTCTCTCTGGTTAGACCAGATCTGAGCCTGGGAGCTCTCTGGC
TAACTAGGGAACCCACTGCTTAAGCCTCAATAAAGCTTGCCTTGAGTGCTTCAAGTAGTGTGTGCCCGTCTG
TTGTGTGACTCTGGTAACTAGAGATCCCTCAGACCCTTTTAGTCAGTGTGGAAAATCTCTAGCAGTGGCGCC
CGAACAGGGACTTGAAAGCGAAAGGGAAACCAGAGGAGCTCTCTCGACGCAGGACTCGGCTTGCTGAAG
CGCGCACGGCAAGAGGCGAGGGGCGGCGACTGGTGAGTACGCCAAAAATTTTGACTAGCGGAGGCTAGA
AGGAGAGAGATGGGTGCGAGAGCGTCAGTATTAAGCGGGGGAGAATTAGATCGCGATGGGAAAAAATTC
GGTTAAGGCCAGGGGGAAAGAAAAAATATAAATTAAAACATATAGTATGGGCAAGCAGGGAGCTAGAACG
ATTCGCAGTTAATCCTGGCCTGTTAGAAACATCAGAAGGCTGTAGACAAATACTGGGACAGCTACAACCATC
CCTTCAGACAGGATCAGAAGAACTTAGATCATTATATAATACAGTAGCAACCCTCTATTGTGTGCATCAAAGG
ATAGAGATAAAAGACACCAAGGAAGCTTTAGACAAGATAGAGGAAGAGCAAAACAAAAGTAAGACCACCG
CACAGCAAGCGGCCGCTGATCTTCAGACCTGGAGGAGGAGATATGAGGGACAATTGGAGAAGTGAATTAT
ATAAATATAAAGTAGTAAAAATTGAACCATTAGGAGTAGCACCCACCAAGGCAAAGAGAAGAGTGGTGCAG
AGAGAAAAAAGAGCAGTGGGAATAGGAGCTTTGTTCCTTGGGTTCTTGGGAGCAGCAGGAAGCACTATGG
GCGCAGCGTCAATGACGCTGACGGTACAGGCCAGACAATTATTGTCTGGTATAGTGCAGCAGCAGAACAAT
TTGCTGAGGGCTATTGAGGCGCAACAGCATCTGTTGCAACTCACAGTCTGGGGCATCAAGCAGCTCCAGGC
AAGAATCCTGGCTGTGGAAAGATACCTAAAGGATCAACAGCTCCTGGGGATTTGGGGTTGCTCTGGAAAAC
TCATTTGCACCACTGCTGTGCCTTGGAATGCTAGTTGGAGTAATAAATCTCTGGAACAGATTTGGAATCACAC
GACCTGGATGGAGTGGGACAGAGAAATTAACAATTACACAAGCTTAATACACTCCTTAATTGAAGAATCGCA
AAACCAGCAAGAAAAGAATGAACAAGAATTATTGGAATTAGATAAATGGGCAAGTTTGTGGAATTGGTTTA
ACATAACAAATTGGCTGTGGTATATAAAATTATTCATAATGATAGTAGGAGGCTTGGTAGGTTTAAGAATAGTT
TTTGCTGTACTTTCTATAGTGAATAGAGTTAGGCAGGGATATTCACCATTATCGTTTCAGACCCACCTCCCAAC
CCCGAGGGGACCCATGCATTGCATCTCAATTAGTCAGCAACCAGGTGTGGAAAGTCCCCAGGCTCCCCAGC
AGGCAGAAGTATGCAAAGCATGCGTCTCAATTAGTCAGCAACCATAGTCCCGCCCCTAACTCCGCCCATCCC
GCCCCTAACTCCGCCCAGTTCCGCCCATTCTCCGCCCCATGGCTGACTAATTTTTTTTATTTATGCAGAGGCCG
AGGCCGCCTCGGCCTCTGAGCTATTCCAGAAGTAGTGAGGAGGCTTTTTTGGAGGCCTAGGCTTTTGCAAA
AAGCTTTCTAGAGGTACCACCGGAGCTTACCATGACCGAGTACAAGCCCACGGTGCGCCTCGCCACCCGCG
ACGACGTCCCCAGGGCCGTACGCACCCTCGCCGCCGCGTTCGCCGACTACCCCGCCACGCGCCACACCGTC
GATCCGGACCGCCACATCGAGCGGGTCACCGAGCTGCAAGAACTCTTCCTCACGCGCGTCGGGCTCGACAT
CGGCAAGGTGTGGGTCGCGGACGACGGCGCCGCCGTGGCGGTCTGGACCACGCCGGAGAGCGTCGAAG
CGGGGGCGGTGTTCGCCGAGATCGGCCCGCGCATGGCCGAGTTGAGCGGTTCCCGGCTGGCCGCGCAGC
AACAGATGGAAGGCCTCCTGGCGCCGCACCGGCCCAAGGAGCCCGCGTGGTTCCTGGCCACCGTCGGCGT
CTCGCCCGACCACCAGGGCAAGGGTCTGGGCAGCGCCGTCGTGCTCCCCGGAGTGGAGGCGGCCGAGCG
CGCCGGGGTGCCCGCCTTCCTGGAGACCTCCGCGCCCCGCAACCTCCCCTTCTACGAGCGGCTCGGCTTCA
CCGTCACCGCCGACGTCGAGGTGCCCGAAGGACCGCGCACCTGGTGCATGACCCGCAAGCCCGGTGCCAG
TTAACGAATTCTAGATCTTGAGACAAATGGCAGTATTCATCCACAATTTTAAAAGAAAAGGGGGGATTGGGG

GGTACAGTGCAGGGGAAAGAATAGTAGACATAATAGCAACAGACATACAAACTAAAGAATTACAAAAACAA
ATTACAAAAATTCAAAATTTTCGGGTTTATTACAGGGACAGCAGAGATCCACTTTGGCGCCGG

Supplementary Table1: Four constructs of minigene
