# Supplementary material for: Exon-intron boundary inhibits m6A deposition, enabling m6A distribution hallmark, longer mRNA half-life and flexible protein coding
Source: Nat Commun. 2023 Jul 13;14:4172. doi: 10.1038/s41467-023-39897-1 (PMC10345190; doi:10.1038/s41467-023-39897-1)
Supplement: Supplementary file 3 — Description of Additional Supplementary Files [file 41467_2023_39897_MOESM3_ESM.pdf]

Description of Additional Supplementary Files

File Name: Supplementary Data 1
Description: **List of qPCR primer pairs.** Here we provide all the 2 qPCR primer pairs and their sequence details, and these primer pairs were used in the 3 experimental validation described in Figure 3.
